# Bottom-up synthesis of molecular nanodiamond from nanographene

**DOI:** 10.1038/s41586-026-10669-3

**Published:** 2026-05-26

**Authors:** Jiaxu Liang, Christopher P. Ender, Nancy C. Forero-Martinez, Ilyes Batatia, Jingyi Liu, Xin Yang, Raul Gonzalez Brouwer, Lev Kazak, Rémi Blinder, Leonardo Cancellara, Nadezda V. Tarakina, Yizhi Liu, Tobias Eklund, Mangalika Sinha, Sarah Köster, Shrikant Bhat, Fabian Rohmann, Andreas Tangemann, Kilian Lee Gallo, Rüdiger Berger, Robert Farla, Alexander Kubanek, Katrin Amann-Winkel, Manfred Wagner, Fedor Jelezko, Klaus Müllen, Gábor Csányi, Robinson Cortes-Huerto, Yingke Wu, Tanja Weil

**Affiliations:** 1 Max Planck Institute for Polymer Research, Mainz, Germany; 2 Institute of Geosciences, Goethe University Frankfurt, Frankfurt, Germany; 3 Institut für Physik, Johannes Gutenberg-Universität Mainz, Mainz, Germany; 4 Engineering Laboratory, University of Cambridge, Cambridge, UK; 5 Institute for Quantum Optics and Center for Integrated Quantum Science and Technology (IQST), Ulm University, Ulm, Germany; 6 Department of Colloid Chemistry, Max Planck Institute of Colloids and Interfaces, Potsdam, Germany; 7 INM-Leibniz Institute for New Materials, Saarbrücken, Germany; 8 Department of Materials Science and Engineering, Saarland University, Saarbrücken, Germany; 9 Institute for X-Ray Physics, University of Göttingen, Göttingen, Germany; 10 Deutsches Elektronen Synchrotron (DESY), Hamburg, Germany

**Keywords:** Nanoparticle synthesis, Optical properties of diamond, Quantum optics

## Abstract

Nanodiamonds hosting colour centres are promising building blocks for quantum technologies, enabling advances in quantum computation^1,2^, nanoscale NMR spectroscopy^3–6^, single-spin magnetometry^7,8^, wide-field quantum imaging^9^ and single-photon sources^10,11^. However, the controlled bottom-up synthesis of ultrasmall and structurally uniform nanodiamonds has remained a challenge, with existing methods producing heterogeneous materials that vary in size, morphology, impurity content and defect quality. Here we show that well-defined, hydrogen-terminated molecular nanographenes serve as chemically confined precursors for high-pressure, high-temperature synthesis of ultrasmall (3–4 nm), monodisperse and highly crystalline molecular nanodiamonds with only a single *sp*^2^ surface reconstruction and produced on a milligram scale. The same bottom-up platform also enables a two-component strategy for incorporating silicon- and germanium-based colour centres during synthesis, yielding SiV^−^ and GeV^−^ emitters without ion implantation, irradiation or post-treatment. Because the nanographene precursor defines both the confined carbon framework and the hydrogen content, this approach provides intrinsic, precursor-level control over nanodiamond size and composition, particularly in the low-nanometre regime relevant for biological and quantum sensing. Molecular nanographenes, ultralarge polycyclic aromatic hydrocarbons, therefore, establish a scalable and modular route to high-quality molecular and fluorescent nanodiamonds and offer a general design principle for tailored quantum materials and nanoscale devices.

## Abstract (web summary)

Bottom-up synthesis from molecular nanographenes produces ultrasmall, uniform nanodiamonds for quantum colour centre incorporation, enabling scalable fabrication of high-quality quantum sensing materials.

## Main

The bottom-up synthesis of atomically defined *sp*^2^-carbon nanostructures, including nanographenes, fullerenes and nanoribbons, has transformed organic electronics and nanoscale sensing^12,13^. By contrast, achieving comparable structural precision for three-dimensional *sp*^3^-carbon nanostructures remains a longstanding obstacle. Nanodiamonds (NDs) provide key features of bulk diamond, including characteristic X-ray diffraction patterns, Raman signatures and also robust atomic colour centres, yet the controlled synthesis of ultrasmall, monodisperse and structurally pure NDs, here referred to as molecular nanodiamonds (m-NDs), has so far remained unknown.

Current ND synthesis routes face inherent limitations. High-pressure, high-temperature (HPHT) methods^14–16^, chemical vapour deposition (CVD)^17,18^ and detonation synthesis^19^ generate heterogeneous materials that vary widely in size, morphology and impurity content. Detonation NDs typically measure 3–5 nm but possess polycrystalline cores and substantial graphitic and nitrogen-related impurities^20,21^. CVD produces polycrystalline particles larger than 30 nm (refs. ^17,18^), whereas top-down milling generates >10 nm particles with irregular shapes and broad size distributions^22^. HPHT processing of metal carbides yields narrower size distributions, while introducing metal contamination^14^. Even the bottom-up HPHT conversion of *sp*^3^-carbon precursors such as adamantanes produces particles spanning 1.5–46 nm in size, demonstrating the absence of reliable control over ND size, morphology and purity^15,23,24^.

Embedding atomic colour centres into NDs enables unique optical and magneto-optical functionalities, but scalable access to high-quality fluorescent NDs (fNDs) remains limited^21,25^. Existing protocols rely on multistep sequences and ion implantation to introduce heteroatom defects and vacancies, vacuum annealing to create colour centres using vacancy diffusion and high-energy ball milling for size reduction^26^. These processes produce particles with broad variations in defect concentration, size, morphology and surface chemistry, which limit performance in quantum sensing and imaging^27^. Furthermore, synthesizing NDs containing silicon-vacancy (SiV^−^) or germanium-vacancy (GeV^−^) centres typically requires complex, multi-component reaction schemes and yields large, heterogeneous and often μm-scale particles^28–30^. A simple and scalable route to ultrasmall fNDs with controlled defect incorporation is therefore highly desirable.

Here, we report the milligram-scale, one-step HPHT synthesis of high-quality m-NDs from an *sp*^2^-hybridized molecular nanographene (C_96_H_30_, m-NG). Using a catalyst-free transformation, we obtain ultrasmall (3–4 nm), monodisperse m-NDs with high structural purity and a single *sp*^2^ surface reconstruction. This bottom-up strategy also enables a straightforward, two-component approach for incorporating SiV^−^ and GeV^−^ colour centres directly during synthesis, without irradiation, annealing or post-processing. Together, these results establish molecular NGs as a versatile and chemically programmable precursor platform for the scalable synthesis of both m-NDs and fNDs with controlled size, composition and optical functionality.

## m-NG-to-m-ND conversion under HPHT

**Fig. 1:**
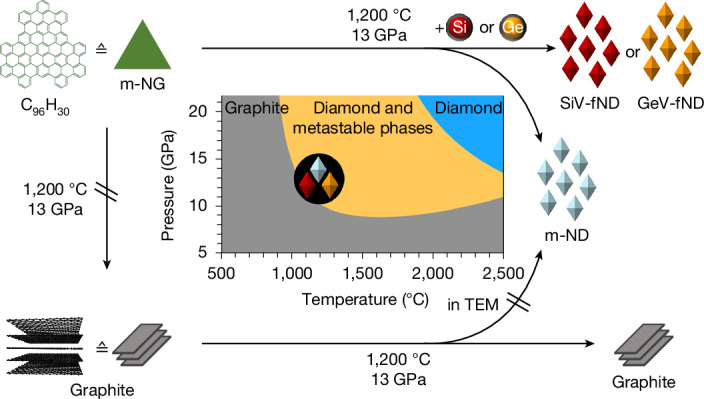
Different behaviours of graphite and nanographene precursors under HPHT conditions. Graphite sheets (grey) and m-NG (green triangle) subjected to 13 GPa and 1,200 °C for 2 h exhibit markedly different outcomes. m-NG converts quantitatively into ultrasmall m-NDs (blue), whereas a two-component mixture of m-NG with tetrakis(trimethylsilyl)silane (Si-) or tetraphenylgermane (Ge-) dopant precursors yields fNDs (red and orange). Under the same conditions, graphite remains largely unchanged.

Bulk graphite converts into diamond under HPHT conditions^31^, but only at extremely high temperatures and pressures (Fig. 1, blue region). At lower pressure and temperature, the kinetic phase diagram of graphite predicts the formation of metastable carbon phases, in which several hybrid structures coexist (Fig. 1, orange region)^31^. To examine how molecular confinement, defined size and hydrogen termination influence this multiphase regime, we subjected several *sp*^2^-hybridized precursors, graphite, coronene (C_24_H_12_), hexabenzocoronene (HBC, C_42_H_18_) and m-NG (C_96_H_30_), to HPHT treatment at 13 GPa and 1,200 °C.

**Fig. 2:**
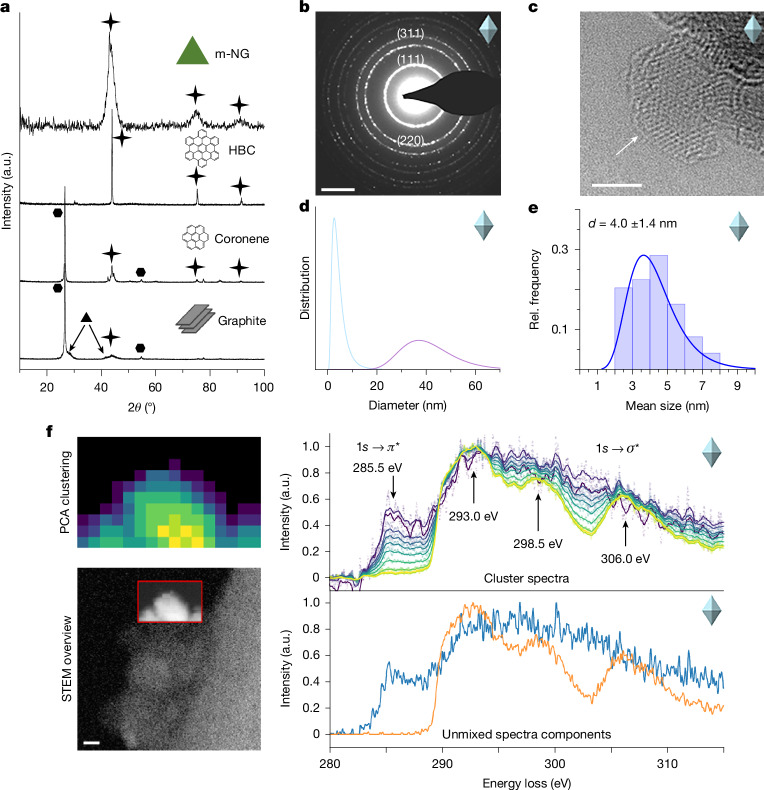
NG-to-ND transformation under HPHT conditions. **a**, XRD patterns of HPHT-treated precursors: graphite yields metastable carbon (triangle) and unreacted graphite (hexagon); coronene converts partially into diamond (star); HBC and m-NG convert fully into diamond. **b**, Selected-area electron diffraction pattern of m-NDs. **c**, HRTEM micrograph of m-ND, evidencing high crystallinity and a single *sp*^2^ surface reconstruction, indicated by the arrow. **d**, SAXS analysis of m-NDs (blue) compared with a commercial 30 nm milled HPHT ND sample (purple). **e**, Size distribution according to HRTEM micrographs (*d* = 4.0 ± 1.4 nm, *N* = 49). **f**, EELS analysis of m-ND using a spectral image acquired with a 0.5 nm step size. Pixels were clustered into 10 regions (plus vacuum) by PCA and *k*-means analysis of the EELS spectra. The normalized average cluster spectra were used to reconstruct the surface (blue) and bulk (orange) components. The presence of the bulk component in the outermost region indicates that the *sp*^2^ surface reconstruction does not exceed 0.5 nm. The blue diamond symbols indicate ND measurements. a.u., arbitrary units. Scale bars, 5 nm^−1^ (**b**); 2 nm (**c**,**f**).

**Extended Data Fig. 1:**
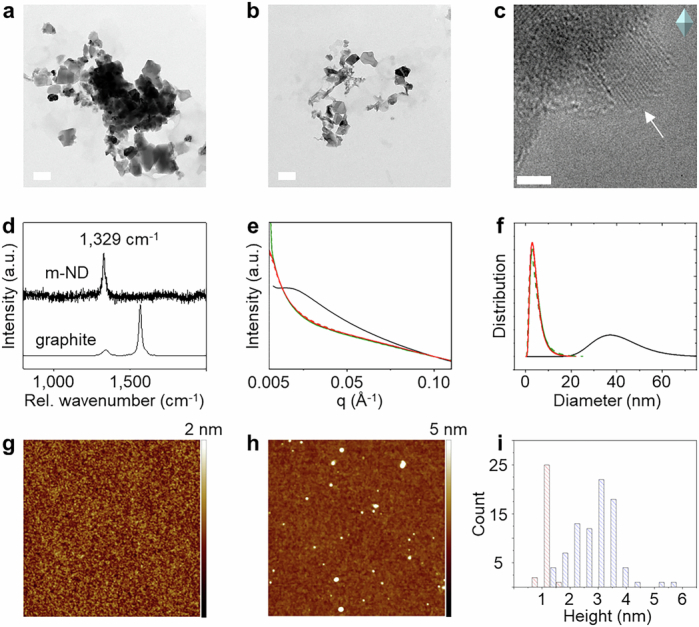
Comparison of HPHT treated hexabenzocoronene and m-NG. **a**, **b**, TEM image of nanodiamonds with broad size distribution from HBC after treatment at 13 GPa and 1200 °C. Scale bars are 200 nm. **c**, HRTEM micrograph of m-ND_ox_, evidencing high crystallinity and a single sp^2^ surface reconstruction, indicated by the arrow. Scale bar, 2 nm. **d**, Raman spectra of untreated m-ND and graphite after HPHT (graphite). **e**, **f**, SAXS analyses of two different m-ND batches (ND1 solid line, ND2 dashed line) using different setups (setup A green, setup B red) compared to commercial 30 nm NDs (black, setup A). **g-i**, AFM images of a blank Si wafer **g** and of the same substrate after m-ND addition **h**. Scan area: 1 × 1 µm^2^. **i**, Distribution of m-ND heights according to height analyses including images **g**, **h**. The mean size is 3.4 ± 1.9 nm, N = 86 particles.

X-ray diffraction (XRD) diffractogram of HPHT-treated graphite shows retained graphite reflections at 3.35 Å, 3.10 Å and 2.17 Å (Fig. 2a), consistent with mixtures of metastable carbon phases and unreacted graphite. Coronene behaves similarly, yielding a combination of diamond and graphitic carbon, in agreement with its thermal instability and known graphitization above 800 °C at high pressure^32,33^. HBC and m-NG, by contrast, convert fully into diamond with no detectable graphitic signatures (Fig. 2a). However, HBC decomposes under these conditions, yielding large, polydisperse NDs (20–350 nm) (Extended Data Fig. 1a,b).

**Extended Data Fig. 2:**
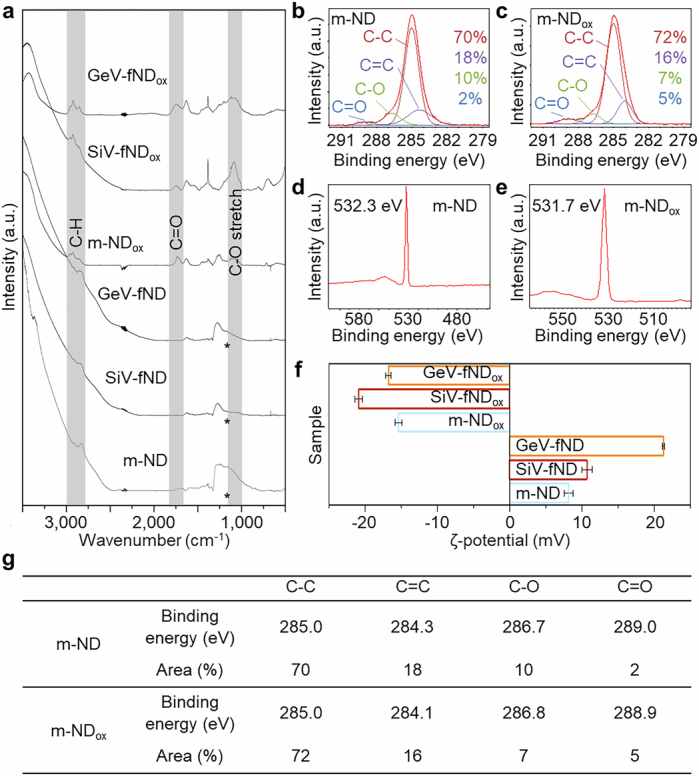
Surface characterisation of Nanodiamonds. **a**, FTIR spectra of untreated nanodiamonds (m-ND, SiV-fND and GeV-fND) and treated nanodiamonds (m-ND_ox_, SiV-fND_ox_ and GeV-fND_ox_). The dip at around 1331 cm^−1^ (*) is associated with Fano-type interference in hydrogenated nanodiamonds^35^. **b**, XPS spectrum of untreated m-ND in the C 1 s region, showing the deconvoluted peak components and their relative areas (%). **c**, XPS spectrum of m-ND_ox_ in the C 1 s region, showing the deconvoluted peak components and their relative areas (%). **d**, XPS spectra of untreated m-ND and **e** m-ND_ox_, focusing on the O-region. **f**, Zeta potential of the untreated (m-ND, SiV-fND, and GeV-fND) and the acid treated samples (m-ND_ox_, SiV-fND_ox_ and GeV-fND_ox_). The positive zeta potentials are indicative of partially hydrogenated ND surfaces, while the negative zeta potentials reflect surface oxidation. Error bars represent s.d. (N = 3). **g**, Table summarizing the binding energies (eV) and relative areas (%) of the deconvoluted C1s components obtained from XPS analysis of m-ND and m-ND_ox_.

By contrast, m-NG, a thermally stable triangular nanographene containing 34 fused rings, 96 carbon atoms and 30 peripheral hydrogens, yields exclusively ultrasmall, monodisperse m-NDs (Fig. 2c–e). A high-resolution Raman spectrum (800–2,000 cm^−1^) shows the characteristic diamond peak at 1,329 cm^−1^ and no detectable G-band (about 1,580 cm^−1^), indicating the absence of graphitic carbon, unlike typical detonation NDs^34^ (Extended Data Fig. 1d). Fourier transform infrared (FTIR) spectra (Extended Data Fig. 2a) show C–H stretching modes centred around 2,830 cm^−1^ and, together with the positive zeta potential of +8 ± 1 mV (Extended Data Fig. 2f), suggest a partial coverage by C–H bonds on the surface of m-NDs^35^. Acid treatment produces oxidized m-NDs (m-ND_ox_), with characteristic C=O features in XPS (Extended Data Fig. 2b–e,g), FTIR signatures and a negative zeta potential of −15 ± 1 mV (Extended Data Fig. 2f).

**Extended Data Fig. 3:**
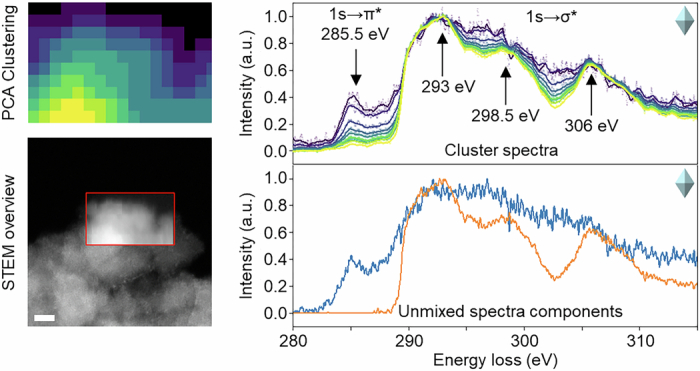
EELS surface characterization of m-ND_ox_ particles. EELS analysis of m-ND_ox_ using a 0.5 nm steps Spectral Image (SI). The pixels in the SI were clustered in 10 regions (and an additional vacuum one) using PCA and k-means clustering on the EELS spectra. The average spectra of the clusters, shown normalised, were then used to reconstruct the EELS spectra of the surface (blue) and bulk (orange) components. The presence of the bulk component in the outermost region indicates that sp^2^ surface reconstruction thickness does not exceed 0.5 nm. The same analysis was performed on m-ND (Fig. 2f), showing qualitatively the same results. Scale bar, 2 nm.

Small-angle X-ray scattering (SAXS), atomic force microscopy (AFM) and high-resolution transmission electron microscopy (HRTEM) consistently show 3–4 nm, monodisperse m-NDs (Fig. 2d,e and Extended Data Fig. 1e–i). HRTEM images of both crude (Fig. 2c) and acid-treated (Extended Data Fig. 1c) m-NDs reveal highly crystalline, faceted and approximately equiaxed nanoparticles. A single *sp*^2^ surface reconstruction is visible, appearing partially detached in some regions and giving rise to slightly wavy edges (Fig. 2c). Electron energy-loss spectroscopy (EELS) spectral imaging (Fig. 2f and Extended Data Fig. 3) confirms that this reconstruction is *sp*^2^-rich, and the presence of such a thin reconstruction (<0.5 nm) is notable compared with the higher *sp*^2^ fractions typically reported for NDs of similar size^34^. Deconvolution of the C1s XPS spectra confirms the presence of C=C species in both m-ND and m-ND_ox_ (Extended Data Fig. 2b–e,g). These combined results indicate that m-NDs possess minimal graphitic carbon and high structural purity.

As the *sp*^2^ network collapses under HPHT conditions, the structure relaxes towards a morphology with minimal surface free energy. Given the fixed hydrogen content and the low diffusivity of hydrogen in diamond, the available hydrogen reservoir dictates surface passivation and therefore the final ND size, yielding uniform nanoparticles.

To quantify size dispersity and morphology, we calculated the standard-deviation-to-mean (s.d./mean) ratio for transmission electron microscopy (TEM), AFM and SAXS. m-NDs exhibit s.d./mean ratios of about 0.35, 0.56 and 0.45, respectively, and commercial fNDs have s.d./mean ratios of approximately 0.55, 0.26 and 0.28, respectively. Despite not undergoing post-synthesis fractionation, m-NDs shows a narrow size distribution. Geometry analysis further shows that milled NDs are disc-like (diameter/height ≈ 4.9), whereas m-NDs are approximately equiaxed (diameter/height ≈ 1.2) based on TEM and AFM, further confirming a more uniform morphology of m-NDs. The diamond Raman line at 1,329 cm^−1^ with full width at half-maximum = 31 cm^−1^ corroborates high crystallinity and size uniformity^36^. Together, these results demonstrate that m-NG undergoes quantitative conversion into structurally pure, ultrasmall NDs, whereas graphitic carbon precursors generate heterogeneous mixtures of metastable phases under identical conditions.

## In situ m-NG-to-m-ND transition

**Extended Data Fig. 4:**
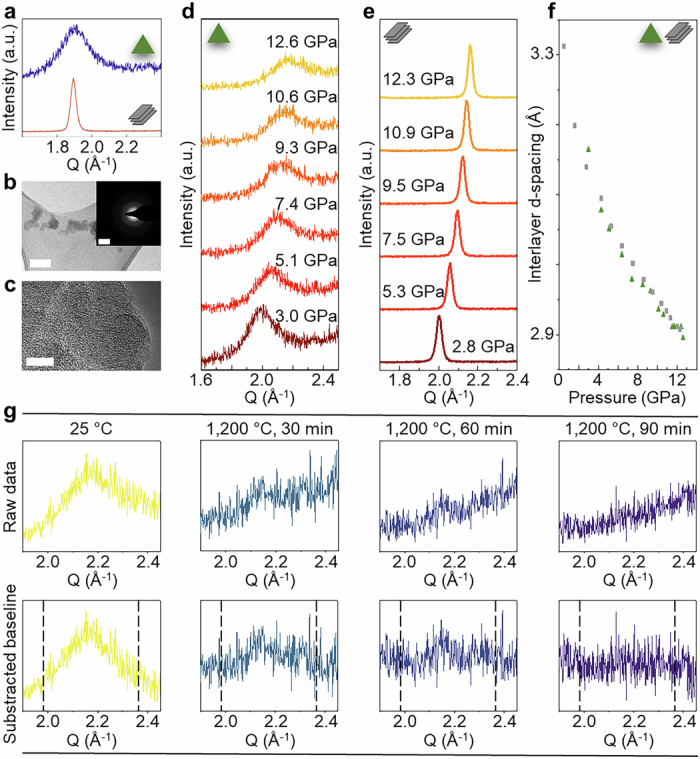
In situ ED-XRD of m-NG and graphite. **a**, ED-XRD of m-NG and graphite under ambient conditions. **b**, **c** TEM images of the untreated m-NG precursor. Scale bars are 100 nm (b) and 10 nm (c). Inset shows SAED with a scale bar of 5 nm^−1^. **d**, ED-XRD of m-NG during compression. **e**, ED-XRD of graphite during compression. **f**, Pressure-dependent interlayer distances of graphite (grey) and m-NG (green) according to in situ ED-XRD (d), (e). **g**, Detailed view of the temperature- and time-dependent in situ ED-XRD measurements of m-NG. The raw and processed data demonstrate the m-NG peak becomes undetectable after maintaining 1,200 °C for 90 min. The dashed lines in the subtracted baseline plots depict the integral borders.

To follow the structural evolution of m-NG during HPHT conversion and to benchmark it against graphite, we performed in situ energy-dispersive X-ray diffraction (ED-XRD) at beamline P61B (DESY, Hamburg)^37^, using a six-ram Hall-type large-volume press. The broadband synchrotron source (30–160 keV) enables real-time tracking of structural changes inside the reaction chamber at 13 GPa and temperatures up to 1,200 °C. Under ambient conditions, the ED-XRD pattern of m-NG resembles that of graphite but shows a broader, less-ordered, (002)-related feature (Extended Data Fig. 4a), consistent with its finite molecular dimensions.

**Fig. 3:**
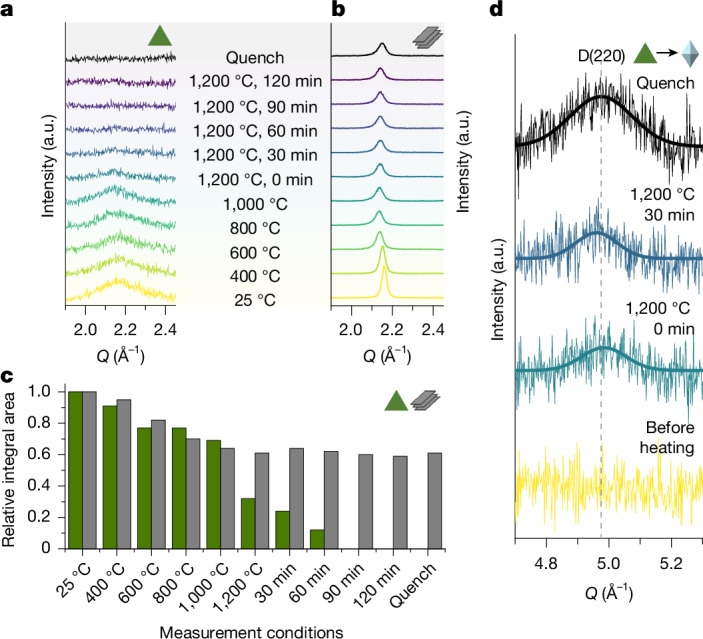
In situ ED-XRD shows a rapid complete transition of m-NG to ND in contrast to graphite. **a**, Temperature-dependent in situ ED-XRD patterns of m-NG at 13 GPa during heating to 1,200 °C and subsequent annealing for up to 120 min, followed by quenching to room temperature. **b**, Corresponding patterns for graphite. For m-NG, the (002)-related peak decreases sharply between 1,000 °C and 1,200 °C and becomes undetectable after 90 min at 1,200 °C (integration boundaries in Extended Data Fig. 4g). Graphite shows only a gradual intensity loss and retains a substantial fraction of its (002) signal after prolonged annealing. **c**, Relative integrated intensities of the (002) reflections of m-NG and graphite as a function of temperature (25–1,200 °C), annealing time (30–120 min) and after quenching. **d**, Evolution of the diamond (220) reflection of m-NG at 13 GPa, showing the onset of ND formation below 1,200 °C, growth during annealing and persistence after quenching. a.u., arbitrary units.

Both precursors were first compressed to 13 GPa before heating. During compression, m-NG and graphite exhibit similar reductions in interlayer spacing, characteristic of graphite-like stacking. The (002) reflection shifts from 3.35 Å at ambient pressure to 2.88 Å for m-NG and 2.92 Å for graphite at 12–13 GPa (Extended Data Fig. 4d–f), confirming the expected pressure-induced contraction of their layered structures. On heating, however, the two precursors follow markedly different pathways. For graphite, the (002) intensity decreases gradually to about 64% of its initial value by 1,000 °C and reaches around 61% after prolonged annealing at 1,200 °C (Fig. 3b,c). This behaviour indicates partial, defect-mediated *sp*^3^ bond formation above about 400 °C, consistent with reports of stacking-fault accumulation^38^, but further conversion is kinetically hindered at 13 GPa. As a result, graphite remains partly unreacted and forms multiple metastable carbon phases under these conditions (Figs. 2a and 3b).

m-NG shows a markedly different evolution. Its (002) intensity decreases to about 69% by 1,000 °C, similar to graphite, but then drops sharply to around 32% between 1,000 °C and 1,200 °C. After annealing at 1,200 °C for 90 min, the m-NG peak disappears entirely (Fig. 3a,c and Extended Data Fig. 4g), demonstrating complete consumption of the molecular precursor. The appearance of the diamond (220) reflection (D(220)) in the m-NG sample at 13 GPa (Fig. 3d) shows that ND formation begins below 1,200 °C, preceding the disappearance of the m-NG (002) feature. The classical graphite–diamond phase diagram does not account for hydrogen-assisted *sp*^2^ → *sp*^3^ transitions relevant to hydrogen-terminated nanographenes. Previous work shows that hydrogen at molecular edges stabilizes *sp*^3^ intermediates and lowers the activation barrier for diamond nucleation under HPHT conditions^39–41^. The high density of edges, curvature and domain boundaries in m-NG provides numerous energetically favourable sites for interlayer bonding and tetrahedral rehybridization. These pathways could rationalize why molecular nanographenes transform rapidly at pressures below those required for bulk graphite.

According to classical nucleation theory^42^, a greater density of nucleation sites leads to smaller and more uniform final crystal sizes. This model aligns with the observed monodisperse 3–4 nm m-NDs and the absence of larger crystallites, which commonly arise from graphitic precursors. By contrast, graphite lacks molecular confinement and exhibits lower local reactivity, yielding fewer nucleation sites and resulting in residual graphite, metastable phases and poorly defined diamond domains. Together, the in situ ED-XRD data show that m-NG undergoes a rapid, quantitative transformation into diamond, whereas graphite remains kinetically trapped, providing direct experimental support for the confinement- and chemistry-assisted mechanism proposed here.

## Size control and rehybridization origin

**Fig. 4:**
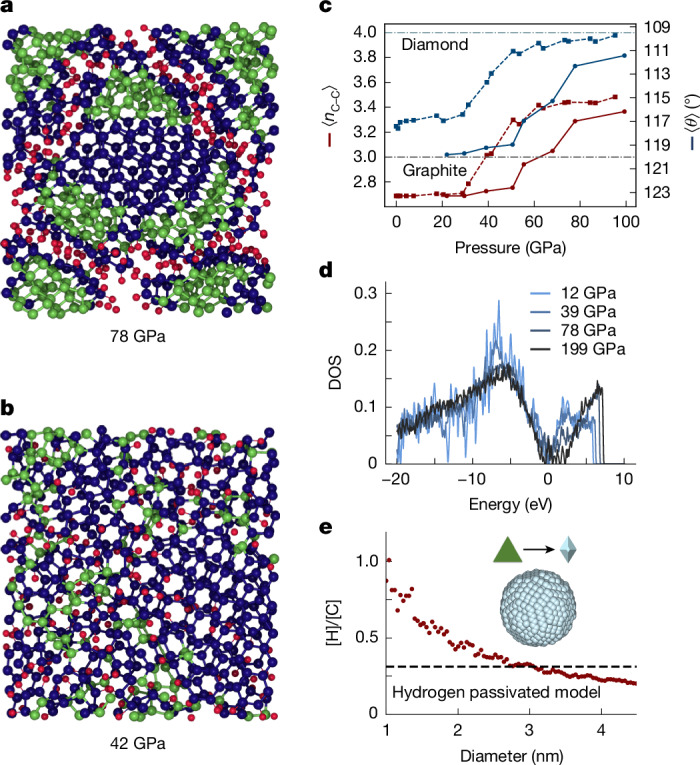
Pressure-induced transformation of NG into diamond-like structures. **a**, Ab initio simulations at 0 K showing the structural evolution of the m-NG supercell as the simulation volume is reduced, corresponding to a pressure of 78 GPa. Increasing pressure induces layer subduction, molecular bending and out-of-plane distortions, signalling the onset of *sp*^2^ → *sp*^3^ rehybridization. **b**, MLIP simulations of the m-NG supercell at 1,500 K and 42 GPa, showing that diamond nucleation occurs at lower pressures with increasing temperature. In **a** and **b**, blue denotes *sp*^2^-hybridized carbon, green denotes *sp*^3^-hybridized carbon and red denotes hydrogen. **c**, Average carbon–carbon coordination number ⟨*n*_C–C_⟩ (red) and average C–C–C bond angle ⟨*θ*⟩ (blue) of the m-NG supercell at 0 K (solid line, ab initio) and 1,500 K (dashed line, MLIP) as a function of pressure from 0 GPa to 100 GPa. Dashed horizontal lines mark the limiting values for ideal graphite (⟨*n*⟩ = 3, *θ* = 120°) and diamond (⟨*n*⟩ = 4, *θ* = 109.5°). The monotonic evolution towards diamond-like coordination reflects the progressive formation of tetrahedral carbon, which occurs at lower pressures as temperature increases. **d**, Electronic density of states (DOS) for different values of pressure in our ab initio simulations. A gap of approximately 4 eV fully develops at 199 GPa, consistent with the gap reported for diamond within the GGA approximation (5.45 eV). **e**, Calculated ratio *r* = [H]/[C] required to passivate spherical hydrogen-terminated ND clusters of diameter *d*. The dashed horizontal line marks *r* = 30/96 = 0.3125 for m-NG, intersecting the model at *d* ≈ 3 nm, consistent with the experimentally observed m-ND diameter.

Building on our experimental findings, we developed a model focusing on the molecular structure of m-NG. The exceptional uniformity of m-NDs can be rationalized by the fixed [H]/[C] ratio of the precursor. m-NG (C_96_H_30_) has *r* = [H]/[C] = 30/96 = 0.3125. Under 13 GPa and 1,200 °C, diamond is thermodynamically favoured, and hydrogen passivation of undercoordinated surface atoms minimizes surface energy at the early stage of m-ND formation. Thus, the most probable ND size is the one for which the required number of surface-passivating hydrogens matches the hydrogen available from the precursor. Calculations of assumed spherical diamond clusters give an intersection at *d* ≈ 3 nm with *r* = 0.3125, in agreement with the measured diameter of 4.0 ± 1.4 nm (Figs. 2e and 4e). Predicted clusters expose mixed (111), (100) and (311) facets (Fig. 4e, inset), consistent with XRD and selected-area electron diffraction; FTIR confirms partial coverage by C–H bonds on the m-ND surface.

**Extended Data Fig. 5:**
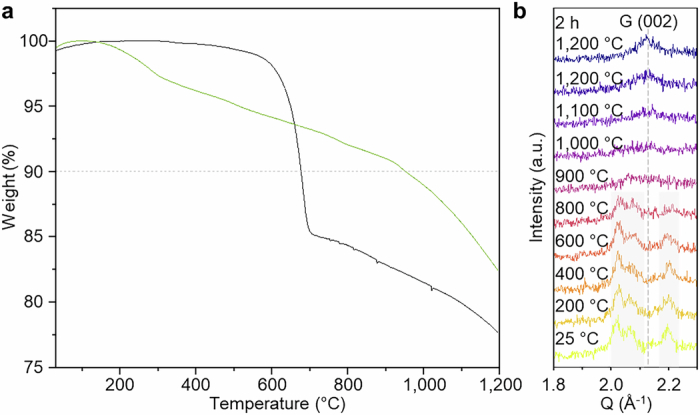
Stability analyses of ND precursors HBC, m-NG and coronene. **a**, TGA curve of HBC (black) and m-NG (green). A weight loss of 10% (dashed line) was measured at 680 °C (HBC) and 950 °C (m-NG), respectively. **b**, In situ ED-XRD of coronene at 13 GPa. Increasing the temperature from 25 °C up to 1200 °C results in the disappearance of the characteristic peaks of coronene (grey box) above 800 °C. Graphite formation starts between 800 and 900 °C depicted by the appearance of the (002) peak of graphite (grey dashed line). The peak becomes more evident after maintaining the temperature at 1200 °C for 2 h.

The [H]/[C] model applies specifically to the intact C_96_H_30_ precursor under the HPHT conditions used here. m-NG is stable up to the onset of diamond formation, ensuring a well-defined hydrogen reservoir for surface passivation. Smaller or less stable precursors, such as coronene or HBC, decompose before diamond formation, altering hydrogen availability and generating amorphous carbon that promotes uncontrolled growth (Extended Data Fig. 5). Consequently, these precursors do not follow stoichiometric size predictions and yield larger, more heterogeneous NDs. Likewise, in two-component dopant systems, decomposition of the dopant precursor and modified nucleation kinetics decouple the final ND size from the initial [H]/[C] ratio, clarifying the scope and limits of the H/C model.

**Extended Data Fig. 6:**
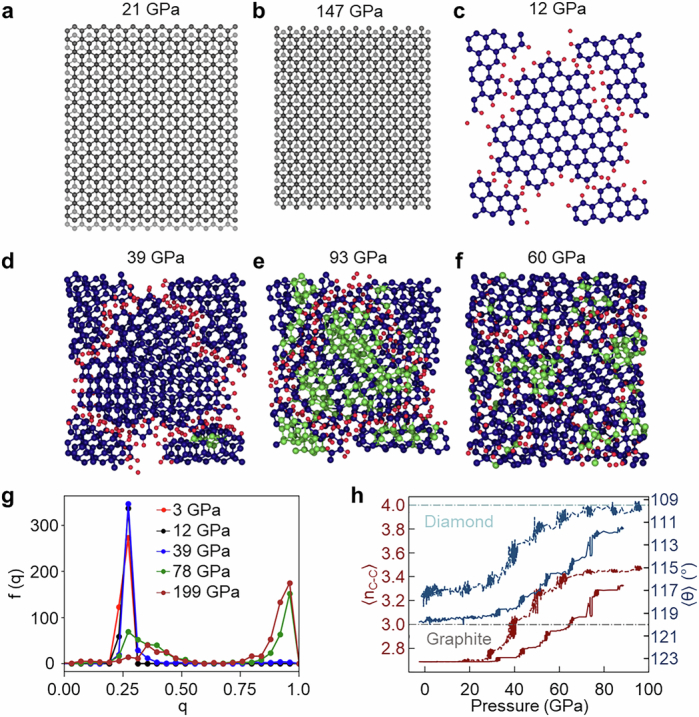
Computational analyses of diamond formation. **a**, **b** Evolution of the graphite supercell upon increasing pressure in the interval 21 <p < 147 GPa obtained by ab initio simulations. **c**, **d** Evolution of the m-NG supercell upon increasing pressure in the interval 12 <p < 39 GPa obtained by ab initio simulations at 0 K. Blue denotes sp^2^-hybridized carbon, green denotes sp^3^-hybridized carbon, and red denotes hydrogen. **e**, **f** MLIP simulations of the m-NG supercell at 0 K and 93 GPa and 200 K and 60 GPa. Blue denotes sp^2^-hybridized carbon, green denotes sp^3^-hybridized carbon, and red denotes hydrogen. **g**, Distribution f(q) of the orientational order parameter q. Following the literature on liquid water^70^, we computed $q=1-(3/8)\,{\Sigma }_{j=1}^{3}\,{\Sigma }_{k=j+1}^{4}$ (cos(*ψ*^*jk*^ + 1/3))^2^, the orientational tetrahedral order parameter where for a given C atom, i, the angles *ψ*^*jk*^ with vertex i and segments ij and ik are calculated for the four nearest neighbours of i. The limits q->0 and q->1 (high pressure) correspond to a fully disordered and a perfect tetrahedral arrangement, respectively. **h**, MLIP-derived average carbon–carbon coordination number ⟨n_C-C_⟩ (red) and average C-C-C bond angle ⟨θ⟩ (blue) of the m-NG supercell at 200 K (solid line) and 1,500 K (dashed line) as a function of pressure from 0 to 100 GPa. Dashed horizontal lines mark the limiting values for ideal graphite (⟨n⟩ = 3, θ = 120°) and diamond (⟨n⟩ = 4, θ = 109.5°). Compared with 200 K (60 GPa), increasing the temperature to 1,500 K shifts the transition to lower pressure (42 GPa).

To explain the atomistic pathway by which m-NG transforms into m-ND under HPHT conditions, we performed ab initio simulations using the Quantum ESPRESSO package^43–45^. The initial configuration consisted of two stacks of three m-NG molecules in a 28.0 × 30.0 × 9.6 Å^3^ periodic supercell (Extended Data Fig. 6c). The system volume was reduced in 8% increments to sample the carbon phase diagram along the zero-temperature isotherm (Fig. 4a and Extended Data Fig. 6d).

As a benchmark, a graphite supercell of comparable size remained structurally stable up to 147 GPa (Extended Data Fig. 6a,b), confirming that the modelling differentiates reactive from non-reactive precursors. The intrinsic time-scale limitations of ab initio molecular dynamics prevent direct simulation of finite-temperature processes. Nonetheless, the computed structural and electronic trends reproduce key experimental signatures of the *sp*^2^ → *sp*^3^ transition and provide mechanistic insight into the high reactivity of m-NG.

We analysed the simulations by tracking coordination numbers, bond-angle distributions and tetrahedral order parameters. Across the 20–100 GPa window, the m-NG supercell evolves from a graphite-like to a diamond-like configuration. The average C–C coordination number (cutoff 1.8 Å) increases from about 2.7 at 20 GPa to about 3.4 at 100 GPa, whereas the mean C–C–C bond angle shifts from about 120° (*sp*^2^-like) to about 111° (close to the 109.5° of *sp*^3^ carbon) (Fig. 4c, solid lines). The *sp*^2^ →* sp*^3^ transition occurs at approximately 78 GPa, which is also confirmed by the orientational tetrahedral order parameter (Extended Data Fig. 6g), which indicates the formation of a diamond-like structure at this pressure. These trends mirror experimental XRD, Raman and HRTEM observations. The electronic density of states (Fig. 4d) shows a gradual disappearance of *π*-states and the emergence of a *σ*-bandgap of about 4 eV. Simulated lattice spacings match experimental values, reinforcing the mechanism.

The lower transition barrier to diamond formation in the case of m-NG in comparison with graphite arises from its confined molecular geometry, hydrogen-terminated edges and progressive layer subduction (Fig. 4a, Extended Data Fig. 6c,d and Supplementary Video 4). Peripheral hydrogen disrupts *π*-conjugation, promotes out-of-plane distortions and stabilizes transient *sp*^3^-rich intermediates, in line with reports that hydrogen reduces the barrier for basal-plane rehybridization and facilitates diamond nucleation under HPHT conditions^39–41^. Our ab initio simulations at 0 K were converged to stringent force and energy criteria and show a consistent evolution towards diamond-like structures in both geometry and electronic configuration.

To assess the role of temperature, we used a machine-learning interatomic potential (MLIP). At 0 K, the simulations reproduce the *sp*^2^ → *sp*^3^ transition at around 93 GPa, consistent with the ab initio results (Extended Data Fig. 6e and Supplementary Video 3). Increasing the temperature markedly lowers the transition pressure to 60 GPa at 200 K (Extended Data Fig. 6f and Supplementary Video 2) and to 42 GPa at 1,500 K (Fig. 4b; Supplementary Video 1). This trend is reflected in the systematic shift to lower pressures observed in the C–C coordination numbers and C–C–C bond-angle distributions at 200 K (Extended Data Fig. 5h, dashed line) and 1,500 K (Fig. 4c, dashed line) compared with the ab initio results at 0 K (Fig. 4c, solid line, and Extended Data Fig. 6h).

At elevated temperatures, the MLIP simulations exhibit enhanced atomic motion at the edges of the m-NG and pronounced molecular bending (Supplementary Video 1). Analysis of bending and *sp*^2^ → *sp*^3^ transition at 1,500 K shows that strong bending develops within the m-NG and precedes the onset of the *sp*^2^ → *sp*^3^ transition (Fig. 4b and Supplementary Video 1). Compared with the 0 K ab initio simulations, finite temperature substantially alters the transformation pathway by introducing reactive regions within the highly bent m-NG. We note that the transition pressures obtained from the rapid compression simulations (100 ps) probably overestimate those under experimental conditions.

The convergence of experimental and theoretical results, including ab initio and MLIP simulations, indicates a confinement- and hydrogen-assisted transformation pathway in which molecular geometry, edge-rich structures, molecular mobility and hydrogen availability jointly promote diamond nucleation by lowering the activation barrier.

## One-step access to SiV^−^ and GeV^−^ NDs

m-NG enables a direct bottom-up route to fNDs containing atomically defined colour centres in a single reaction step. Only two molecular precursors, m-NG and a dopant-bearing compound, are required. Using tetrakis(trimethylsilyl)silane as the silicon source, we obtain SiV-fNDs under the same HPHT conditions as for m-NDs (13 GPa, 1,200 °C). The synthesis produces crystalline fNDs without irradiation, implantation, annealing or post-synthetic activation, and without extensive purification steps.

**Fig. 5:**
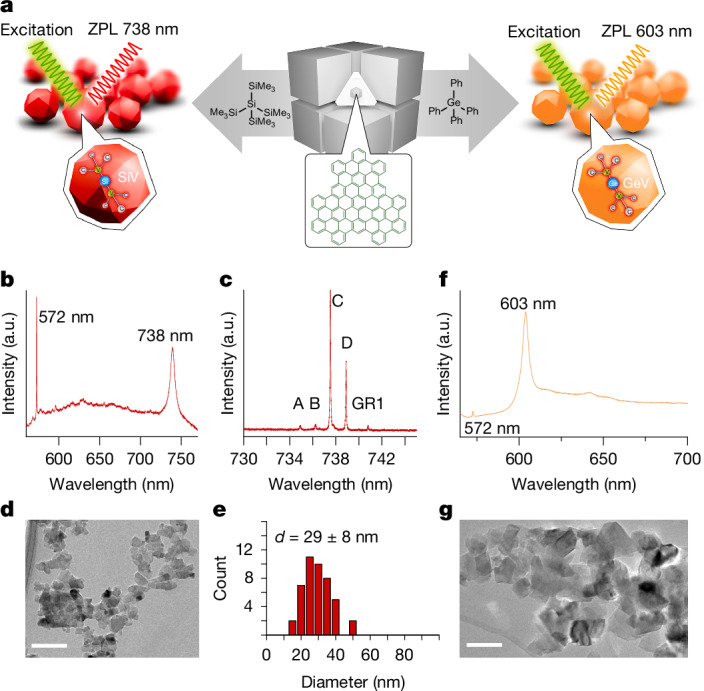
One-step HPHT synthesis of fNDs containing SiV^−^ and GeV^−^ centres. **a**, Schematic of the two-component HPHT synthesis using m-NG and dopant precursors to form SiV-fNDs and GeV-fNDs. **b**, Room-temperature PL spectrum of SiV-fNDs showing the diamond Raman line at 572 nm and the ZPL at 738 nm. **c**, Low-temperature (4 K) PL spectrum resolving the four optical transitions (A–D) and a weak GR1 signature. The splitting of the lines exceeds the spin–orbit coupling characteristic of a strained diamond lattice^51^. **d**, TEM image of SiV-fNDs. **e**, Size distribution of SiV-fNDs obtained from TEM analysis (mean diameter = 29 ± 8 nm; *N* = 45). **f**, Room-temperature PL spectrum of GeV-fNDs showing the diamond Raman peak and the ZPL of the GeV^−^ centre at 603 nm. **g**, TEM image of GeV-fNDs. a.u., arbitrary units. Scale bars, 100 nm (**d**,**g**).

TEM images of crude SiV-fNDs show diamond particles with an average diameter of 29 ± 8 nm and a narrow size distribution (Fig. 5d,e), contrasting with the broad heterogeneity commonly observed in earlier HPHT or CVD routes^29,30^. FTIR indicates C–H termination before acid treatment and increased C=O rich surfaces afterwards (Extended Data Fig. 2a), with zeta potential changing from +11 ± 1 mV to −21 ± 1 mV (Extended Data Fig. 2f), confirming the expected surface chemistry of crude and oxidized fNDs.

The room-temperature photoluminescence (PL) spectrum (Fig. 5b) shows the SiV^−^ zero-phonon line (ZPL) at 738 nm (refs. ^11,46^), confirming incorporation of the defect. Low-temperature PL (4 K, Fig. 5c) resolves the four canonical SiV^−^ transitions (A–D) under 532 nm excitation. A weak 741 nm GR1 feature, arising from neutral vacancies, indicates vacancy formation during HPHT synthesis even in the absence of irradiation.

**Extended Data Fig. 7:**
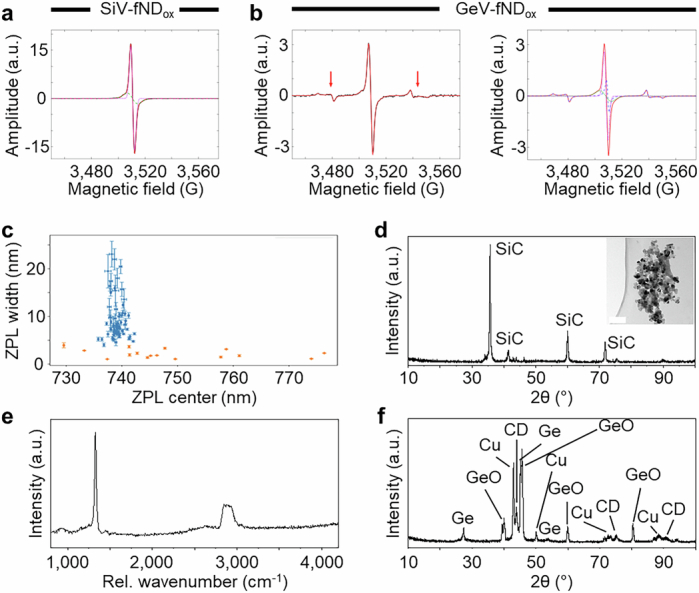
Spectral and optical properties of fluorescent nanodiamonds and control experiments. **a** and **b**, X-band continuous-wave EPR spectra and fits, for SiV- and GeV-doped sample. **a**, SiV-doped sample: experimental spectrum (black line) and fit (red line). The fit is performed with two components, labelled according to their relative linewidth as ‘broad’ (green, dashed) and ‘narrow’ (purple, dotted). The fit yields *g* = 2.0031 and *g* = 2.0030 for the broad and the narrow component, and corresponding peak-to-peak linewidths of 0.80 mT and 0.28 mT. **b**, Spectrum and fit for the GeV-doped sample. Left subfigure: Experimental spectrum (black line) and fit with three contributions (red line): *g* ~ 2 components (broad and narrow), and substitutional nitrogen (P1 centers). Arrows indicate the groups of P1 hyperfine lines corresponding to the nuclear spin state of the ^14^N, *m*_I_ = −1, + 1. Right subfigure: Representation of the individual fit contributions. A microwave frequency of 9.84 GHz and a microwave power of 4.73 μW were used in the measurements.**c**, Measurement of ZPL centre position and ZPL width of 88 particles at room temperature. The data reveals two different groups of emitters. While Group V (blue) has a fixed central ZPL at around 738 nm but a large spread in ZPL width between about 5 and 25 nm, Group H (orange) has a constantly low ZPL width below 5 nm but a large spread of ZPL positions between 730 nm and 780 nm. **d**, XRD of Tetrakis(trimethylsilyl)silane treated for 2 h at 13 GPa and 1,200 °C. Inset: TEM image of the product from HPHT treated Tetrakis(trimethylsilyl)silane. The scale bar represents 200 nm. **e**, Raman spectrum of Tetraphenylgermane treated for 2 h at 13 GPa and 1,200 °C. **f**, XRD of Tetraphenylgermane treated for 2 h at 13 GPa and 1,200 °C.

EPR measurements on SiV-fND_ox_ show a *g* ≈ 2 resonance (Extended Data Fig. 7a), attributable to near-surface defects and unpaired spins^47^. No resolved SiV-related EPR signatures are detected, probably because of spectral overlap and the absence of zero-field splitting or hyperfine structure. The KUL8 centre (*S* = 1/2), previously proposed to originate from SiV^−^ (ref. ^48^), cannot be identified unambiguously; SiV^0^ (*S* = 1) is not detected (<500 ppb). Analysis of 88 individual ZPLs at room temperature shows two emitter groups (horizontal and vertical) (Extended Data Fig. 7c), matching the results in ref. ^49^ and indicating distinct, but well-defined, local environments. Linewidths lie within ranges reported for high-quality CVD-grown SiV^−^ fNDs. Notably, no nitrogen-vacancy centres are detected, even without fluorine-containing suppressors used previously^29,30^.

Replacing the silicon precursor with tetraphenylgermane under the same HPHT conditions yields GeV-fNDs. Room-temperature PL shows a strong GeV^−^ ZPL at 603 nm (ref. ^50^) (Fig. 5f), and TEM confirms crystalline ND morphology (Fig. 5g). FTIR and zeta potential trends mirror those of SiV-fNDs (Extended Data Fig. 2a,f). GeV^0^ (*S* = 1) is not detected (<500 ppb) by EPR; signals from g ≈ 2 centres and substitutional nitrogen (P1) are observed (Extended Data Fig. 7b). Charge-state identification is supported by the presence of donor species: EPR spectra of GeV-fNDs show P1 centres, whereas the absence of NV^−^ emission reflects the deeper charge-transition levels of SiV^−^ and GeV^−^ in weakly compensated material.

Together, these results identify m-NG as a versatile molecular scaffold for bottom-up HPHT synthesis of fNDs containing SiV^−^ and GeV^−^ centres, with controlled defect incorporation, well-defined optical signatures, and narrower size distributions than in previous reports. The final fND size is decoupled from the [H]/[C] ratio, reflecting the decomposition of dopant precursors and modified nucleation kinetics in two-component systems, thereby clarifying the scope and limitations of precursor-encoded size control.

## Discussion

Our results establish a molecularly defined bottom-up route for synthesizing ultrasmall and structurally uniform NDs under HPHT conditions. In contrast to graphite, which remains kinetically trapped and yields multiphase mixtures at 13 GPa and 1,200 °C, hydrogen-terminated nanographenes undergo rapid and complete *sp*^2^ → *sp*^3^ rehybridization, producing 3–4 nm monodisperse ND crystals with a single *sp*^2^ surface reconstruction. In situ ED-XRD, atomically resolved HRTEM and EELS together show that the diamond lattice extends nearly to the surface, whereas ab initio simulations reproduce the loss of *π*-states, the emergence of a *σ*-bandgap and the evolution towards tetrahedral coordination. Additional MLIP simulations reveal a temperature-dependent introduction of reactive centres into the interior of the m-NG, apart from the reactive edges. This behaviour can be attributed to extensive molecular bending within the m-NG and enhanced atomic mobility, particularly at the m-NG edges. The close agreement between experiment and theory, including ab initio and MLIP, suggests a confinement- and hydrogen-assisted transformation pathway, in which molecular geometry, abundant edge sites, molecular mobility and hydrogen availability act cooperatively to reduce the activation barrier for diamond nucleation.

By fixing both the carbon framework and the hydrogen reservoir at the molecular level, this approach intrinsically constrains crystal growth, enabling predictive size selection in the low-nanometre regime, an ability not accessible to existing HPHT, CVD or detonation methods. Extending this design to two-component precursor mixtures yields SiV^−^ and GeV^−^ fNDs in a single step, without implantation, annealing or mechanical processing, and with markedly narrower size and defect distributions than previous methods.

Together, these findings define a general molecular blueprint for bottom-up ND synthesis, providing direct control over lattice structure, surface chemistry and embedded defects. This platform establishes a scalable foundation for quantum sensors, integrated photonic emitters and chemically programmable diamond nanomaterials and highlights molecular nanographenes as a versatile class of precursors for precision-engineered *sp*^3^-carbon architectures.

## Methods

### Precursor preparation

m-NG was synthesized through a Diels–Alder reaction of 1,3,5-triethynylbenzene with excess cyclopentadienone, followed by oxidative cyclodehydrogenation. A detailed procedure was previously reported^52^. Graphite (flakes, 99% carbon basis, −325 mesh particle size (≥99%), natural), perchloric acid (60%) and sulfuric acid (95–97%) were purchased from Sigma-Aldrich, coronene (>98%), tetrakis(trimethylsilyl)silane (>98%) and Tetraphenylgermane (>95%) were purchased from TCI, hexabenzocoronene (97%) was purchased from BLDpharm, commercial 30 nm ND (MSY 0-0.03 μm) was purchased from Pureon and used without further treatment.

### Sample synthesis

The HPHT experiments were conducted using a Hall-type LVP Aster-15 at beamline P61B at PETRA III in Hamburg, Germany. The Aster-15 LVP compresses a cubic space with six independent rams controlled by a programmable logic controller, each providing a maximum load of 5 MN, with a total maximum load of 15 MN. For the experiments at 13 GPa, the precursor was loaded into a Ti capsule, which was then placed in an MgO sleeve with an inner diameter of 2.0 mm and a length of 2.4 mm, using a 14/8 cell assembly with graphite as the heater material. Pressure was estimated based on previously obtained calibration curves when the experiments were performed without in situ ED-XRD measurements.

On reaching the target pressure, increased at a rate of approximately 3 GPa per hour, the samples were heated at a rate of 20 °C min^−1^ to the target temperature using a 3.6 kW a.c. heating system. The temperature was then maintained for 2 h before the samples were quenched by turning off the electric power supply. The temperature was measured or calibrated with type-C W–Re thermocouples. Finally, the pressure was slowly released over a period of 9–12 h.

The sample synthesis procedures were also replicated at MPIP using a Voggenreiter Mavo Press LP 1000-540/50 equipped with a multi-anvil module. Temperature was pre-calibrated using a type-D thermocouple (W97Re3–W75Re25) from OMEGA Engineering. The HPHT experiments used the same cell assemblies as those at the P61B beamline, following similar heating and compression–decompression protocols.

HPHT control experiments using the dopant molecules tetrakis(trimethylsilyl)silane (Extended Data Fig. 7d) and tetraphenylgermane (Extended Data Fig. 7e,f) in the absence of the m-NG precursor support the necessity of m-NG for the synthesis of fNDs. TEM analysis of the m-NG precursor before HPHT treatment (Extended Data Fig. 4b,c) further confirms the absence of nanoparticles, in particular NDs, in the starting material.

### In situ ED-XRD measurement

The P61B LVP is mounted on five independent alignment stages that allow for translation and rotation of the press to align with the incident synchrotron X-ray beam. The detection system for synchrotron X-rays at the P61B beamline features two high-purity germanium solid-state detectors by Mirion (Canberra) for ED-XRD. These detectors are positioned at 2*θ* angles of 5° and 7°, which are used to investigate the *q* ranges of 1.5–2.5 Å^−1^ and 2.5–6.5 Å^−1^, respectively, in the ED-XRD patterns. Before each run, the channel-energy relations of the detectors were confirmed using Pb fluorescence, caused by a small number of scattered X-rays from the imperfect shielding of the Ge element of the detector. Pb fluorescence is characterized by Kα_1_, Kα_2_ and Kβ_1_ (74.97 keV, 72.80 keV and 84.94 keV, respectively). The goniometer angles of both Ge-SSDs were set to the desired diffraction angles and calibrated by taking XRD of LaB_6_ under ambient conditions. During the compression process, pressure was determined using ED-XRD and the equation of state of MgO, which is located near the sample in the cell assembly, under HPHT conditions. During the heating process, ED-XRD patterns of the sample were acquired in 100 °C increments, with an exposure time of 50 s per frame and a total of 6 frames.

The analysis of peak intensity during heating at 13 GPa was performed by (1) baseline subtraction using a B-spline function and (2) calculation of the integrated area within the range of 1.992 Å^−1^ to 2.365 Å^−1^ (Extended Data Fig. 4g). The peak of m-NG became undetectable after holding the temperature at 1,200 °C for 90 min, and no integrated area was calculated. The evolution of the D(220) peak was analysed by (1) baseline subtraction using a B-spline function and (2) peak fitting with a Gaussian function.

### Acid treatment

The ND samples were oxidized using a mixture of H_2_SO_4_, HNO_3_ and HClO_4_ (volumetric ratio 1:1:1) and heated to 90 °C under sonication. Afterwards, the samples were washed with MilliQ-H_2_O and centrifuged for 20 min at 4,500 rpm. These steps were repeated for three times.

### XRD measurement

Powder XRD measurements were performed on a Rigaku SmartLab diffractometer using a Cu Kα anode (*λ* = 1.5406 Å). The powders were placed on a zero reflection Si substrate.

### Raman spectroscopy

Raman spectroscopy was performed using an Oxford Instruments WITec Raman microscope (alpha 300 R) equipped with an Olympus SLMPan N 50× objective. The measurements were performed with a 532 nm excitation laser with a spot size of around 1.0 µm, typically operating at an output power of 0.1–0.5 mW. The Raman spectra were acquired with the 1,800 grooves mm^−1^ grating of the WITec UHTS 300S (VIS-NIR) spectrograph in combination with an Andor DR316B-LDC-DD CCD detector. Raman spectra of m-NDs were recorded using a laser power density of <150 µW µm^−^^2^ to avoid laser-induced modifications of the NDs.

### X-ray photoelectron spectroscopy

Aqueous solutions (10 µl) were deposited on a silicon substrate and dried at room temperature. XPS was conducted using a Kratos Axis UltraDLD spectrometer 3 (Kratos) using an Al Kα excitation source with a photon energy of 1,487 eV. The data were acquired in the hybrid mode using a 0° take-off angle, defined as the angle between the surface normal and the axis of the analyser lens. Detailed region X-ray photoelectron spectra were collected with the analyser pass energy set at 80 eV, and a linear background was subtracted for all peak quantifications. The peak areas were normalized by the manufacturer-supplied sensitivity factors and surface concentrations were calculated using CasaXPS software. The background was subtracted by a Shirley correction. To extract the components of the C 1*s* a curve fitting procedure was carried out using Voigt functions with a Gaussian to Lorentzian ratio of 30%. C 1*s* high-resolution spectra were collected with analyser pass energy of 20 eV. Neutralizer was always used during spectra collection.

### Thermogravimetric analysis

Thermogravimetric analysis was carried out on a Mettler Toledo ThermoSTAR TGA/DSC 3^+^ in a nitrogen atmosphere. The samples (about 10 mg) were heated from room temperature to 1,200 °C, with the N_2_ purging rate set as 30 ml min^−1^, and a heating rate of 10 K min^−1^.

### FTIR spectroscopy

The FTIR measurements were performed using a Bruker VERTEX 70 in transmission geometry. The sample material was prepared in pellet form within an infrared-transparent matrix. For the analysis, 250 mg of KBr powder (previously stored in an oven at 70 °C) was mixed with 0.5 mg of the ND sample. The mixture was shaken for 30 s in a Perkin Elmer Vibrating Mill. Afterwards, the mixture was transferred into an evacuable die and placed in a Perkin Elmer hydraulic press. The die was evacuated for at least 30 min. Following this, a pressure of 10 tonnes was applied and held for 60 min. The resulting pellets were then measured with a resolution of 1 cm^−1^ and a scan time of 32 scans, covering a range from 400 cm^−1^ to 4,500 cm^−1^. Before each measurement, the chamber containing the pellet was purged with nitrogen for 30 min.

### Zeta potential measurement

The zeta potential measurements were conducted at 25 °C using a Zetasizer Nano Z (Malvern Panalytical). The samples were diluted with MilliQ water, and 100 µl sample was transferred into a High Concentration Zeta Potential Cell (ZEN1010 cell) for measurement. Three measurements were performed on each sample, with automatically determined runs for each measurement.

### PL measurement at room temperature

The NDs were dispersed in purified water, placed in an ultrasonic bath (EMAG, Emmi-40HC) for around 5 min, and then drop-cast onto a fused silica substrate. Previously, the substrate was cleaned using first-contact, acetone and isopropanol. For the PL measurements, a custom-built confocal microscope was used with an air objective (Olympus, MPlanApo50×, 0.95 numerical aperture (NA)) and an oil objective (Olympus, UPlanSApo60×, 1.35 NA). For the excitation, a 532 nm laser (Laser Quantum gem 532) was used with an excitation power of 0.8 mW. The fluorescence was detected with an avalanche photodiode (Excelitas, AQRH-14-FC) and a spectrometer (Princeton Instruments, SpectraPro HRS-500). The laser light was blocked by a 580 0.nm long-pass filter. The width and centre wavelength of the ZPLs were determined by fitting a Lorentzian to the ZPL peak for each acquired spectrum in a wavelength range of 724–780 nm.

### PL measurement at 4 K

The NDs were dissolved in purified water, sonicated for 3 min, and subsequently subjected to spin-coating onto a sapphire substrate that had been previously cleaned using soft O_2_ plasma. Spectral measurements were performed in a continuous-flow cryostat (CryoVac) operating at 4 K, with temperature monitored by a CryoVac TIC500 controller. The sample was mounted on the cold head, which was attached to a piezo positioner. The objective lens (Olympus UMPlanFI, 100×, NA 0.95) was positioned on a piezo stage connected to the room-temperature section of the cryostat. Excitation was performed using a continuous-wave 532 nm laser (Laser Quantum GEM) with an output power of 0.48 mW. The emitted light was detected using an avalanche photodiode (APD, Excelitas Technology SPCM-AQRH-16) with a 735/28 bandpass filter. A flip mirror in the detection path allowed the emission light to be directed towards an optical spectrometer. The spectrometer consisted of a Czerny–Turner monochromator (Princeton Instruments, Acton SP2500) and a CCD camera (PIXIS 100BR). A diffraction grating with 1,200 lines mm^−1^ (blaze 750 nm) was used during the experiments. To block the excitation light, a 550 nm long-pass filter was placed at the spectrometer entrance.

### AFM measurements

AFM measurements were performed using a Bruker Dimension ICON microscope (Nanoscope 5 controller, Software 9.7r1sr8) operated in tapping mode. For imaging, we used cantilevers with a nominal resonance frequency of 300 kHz and a nominal spring constant of 26 N m^−1^ (OTESPA, OPUS made by µ mash). Images are recorded at a scan speed of around 1 Hz with 512 pixels per line and 512 lines. All images were plane corrected by an offset and a tilt (first-order polynomial). Feature heights were extracted by analysing the maximum height difference inside a small box around the feature.

### TEM measurements

The TEM images and selected-area electron diffraction were obtained with a field-emission transmission electron microscopy (FE-TEM, Tecnai G2 F20 U-TWIN). The samples were dispersed in isopropyl alcohol and were sonicated (10 W, 30 s, 10 cycles) using a Hielscher Sonicator (UP200St-G) equipped with S26d11×10 Vial-Tweeter-Sonotrode. A total of 4 µl of the solution was dropped on a carbon-filmed grid. The images were analysed with DigitalMicrograph software. The scanning transmission electron microscope (STEM) study was performed using a double Cs-corrected JEOL JEM-ARM200F STEM operated at 80 kV and equipped with a cold-field emission gun (all data were recorded at an emission current of 10 µA) and a Quantum GIF system. High-resolution TEM images were recorded using a Gatan OneView camera. Bright field and annular dark field STEM images were collected at a probe convergence semi-angle of 25 mrad. EELS spectral images (SIs) were collected at different positions in the samples using 0.025 eV per channel dispersion and 1 s exposure time per spectrum. Drift correction was applied after each 2 pixels of SI. The data from SIs was processed using open-source Python libraries (Hyperspy^53^, Scipy^54^ and Scikit^55,56^). Signal denoising was performed using pixelwise spike removal and a global non-negative matrix factorization, keeping five components out of six. For region segmentation, the data were decomposed into six orthogonal principal components, the pixel scores of which were used for *k*-means clustering. At first, 50 clusters were used to isolate the vacuum region, then 10 clusters on the sample itself. The average spectra from the 10 regions were finally used to unmix the surface and bulk signals by a non-negative least squares optimization.

### EPR spectroscopy

The continuous-wave EPR data were recorded with an X-band EPR system (Bruker ELEXSYS II E580) with an ER4122 SHQE cavity, using the software xEPR for acquisition. The samples were inserted inside EPR quartz tubes, Wilmad 707-SQ-100M, of 4 mm outer diameter. The resonance quality factor was *Q* ≈ 8,000. The spectra were fitted using the Matlab package Easyspin^57^.

### SAXS measurements

SAXS measurements were performed using two different setups. In both setups, the ND powder samples were prepared between two pieces of magic scotch tape. The first measurement (setup A) was performed using a Xeuss 2.0 (Xenocs) setup. A Genix 3D source (Xenocs) was used at a wavelength of 1.54 Å (Cu Kα radiation), 50 kV and 600 μA. The scattered signal was recorded at a distance of 2,500 mm using a pixel detector (Pilatus3R 1M, 981 × 1,043 pixels, pixel size 172 × 172 μm Dectris). A beamstop with a size of 5 mm was placed directly in front of the detector to block the primary beam. The second SAXS measurement (setup B) was also performed using CuKα radiation (RigakuMicroMax 007 X-ray generator, Osmic Confocal Max–Flux curved multilayer optics). Two-dimensional (2D) diffraction patterns of these samples were recorded using a Mar345 image plate detector at a sample-detector distance of 2,120 mm. Intensity profiles were recorded by radial averaging of the two-dimensional (2D) datasets as a function of the scattering vector, **q** = (4π/*λ*)sin(2*θ*/2), where 2*θ* is the scattering angle. The azimuthal integration was performed using PyFAI. Calibration was performed using a reference sample (AgBH). In setup A, diffractograms were collected for 10 min at room temperature. In setup B, they were collected for 2 h at room temperature. Moreover, in the second setup, commercial diamond samples attached between two pieces of magic scotch tape were measured for comparison.

### SAXS analysis

X-ray scattering occurs when a sample contains regions of differing electron densities, Δ*ρ*(**r**). The scattering intensity *I*(**q**) from the 2D images was calculated by performing azimuthal integration. This intensity can be expressed in terms of the form factor *F*(**q**) and the structure factor *S*(**q**):

$$I({\bf{q}})={|F({\bf{q}})|}^{2}S({\bf{q}})$$

First, the structure factor *S*(**q**) is calculated from the particle density function *n*(*r*) and the average density *n*_0_, describing the correlation between particles:

$$S({\bf{q}})=1+\int (n(r)-{n}_{0}){{\rm{e}}}^{({\rm{i}}{\bf{q}}\cdot {\bf{r}})}{\rm{d}}r$$

The NDs are investigated as a powder sample with an unknown packing fraction; we assume the particles are randomly distributed and oriented. The *S*(**q**) was obtained by fitting the correlation length and packing fraction. Second, the form factor *F*(**q**) depends on the shape and size of each particle:

$$F({\bf{q}})=\int \Delta \rho ({\bf{r}}){{\rm{e}}}^{({\rm{i}}{\bf{q}}\cdot {\bf{r}})}{\rm{d}}r$$

The particle size fitting was conducted using SmartLab Studio II software (https://rigaku.com/) as well as SasView (http://www.sasview.org/). By fitting the parameters to *I*(**q**), the average particle size *R*_0_ and the shape parameter *N* were determined using a non-linear minimum squares method. We used spherical fits for the two ND samples and an ellipsoidal fit for the commercial diamond. The size distributions (fitted with SmartLab Studio II software) are shown in Extended Data Fig. 1f.

Spherical particles with a radius *R* are described by the following equation:

$${F}^{{\rm{s}}{\rm{p}}{\rm{h}}{\rm{e}}{\rm{r}}{\rm{e}}}(q)=\Delta \rho \times (4\pi /{q}^{3})[\sin (qR)-qR\,\cos (qR)]$$

For ellipsoidal particles, the form factor is expressed as

$$\begin{array}{c}{F}^{{\rm{e}}{\rm{l}}{\rm{l}}{\rm{i}}{\rm{p}}{\rm{s}}{\rm{o}}{\rm{i}}{\rm{d}}{\rm{a}}{\rm{l}}}(q,{\varphi },R,a)=\Delta \rho \times 4\pi a{R}^{3}[\sin (qRf(a,{\varphi }))\\ \,-\,qRf(a,{\varphi })\cos (qRf(a,{\varphi }))]/{(qRf(a,{\varphi }))}^{3}\end{array}$$

where

$$f(a,\varphi )=\mathrm{sqrt}({\sin }^{2}(\varphi )+{a}^{2}{\cos }^{2}(\varphi ))$$

where *R*_0_ is the average radius for spherical particles and the maximum cross-sectional radius for ellipsoidal particles. *M* is the size distribution parameter.

### Ab initio simulations

To investigate structural transformations and high-strain resulting from high-pressure conditions, ab initio calculations using the plane wave approach implemented in the Quantum ESPRESSO package^43–45^ have been performed. These calculations have used the generalized gradient approximation of Perdew–Burke–Ernzehof^58^, norm-conserving pseudopotentials of the Troullier–Martins type^59^ for carbon and hydrogen and a kinetic energy cutoff of 54 Ry for expanding the Kohn–Sham orbitals. The periodicity of the system was enforced in all spatial directions. Dispersion interactions were included in the calculation using the approach outlined in ref. ^60^.

The graphite supercell, with initial dimensions 14.8 × 17.1 × 25.6 Å^3^, consisted of 768 C atoms. The m-NG supercell was prepared by joining two C_96_H_30_ molecules on a plane and intercalating two adjacent long edges. This dimer structure was replicated perpendicular to the plane to produce a stack of three layers with 576 C atoms and 180 H atoms. The initial box has dimensions 28.0 × 30.0 × 9.6 Å^3^.

Structure optimization to find the closest local minimum, relying on force and energy minimization, has been performed using the quasi-Newton algorithm of Broyden–Fletcher–Goldfarb–Shanno^61–64^. The volume has been reduced isotropically in steps corresponding to an 8% decrease to increase the pressure of the samples. Local structural optimization was carried out for every step. This procedure corresponds to investigating the phase diagram of the systems along the zero-temperature isotherm. In the case of graphite, all steps converge with a total force smaller than 0.01 Ry bohr^−1^, all components of all forces are smaller than 0.001 Ry bohr^−1^, and the energy is smaller than 10^−6^ Ry. The m-NG structures were converged such that the maximum atomic force component was below 0.001 Ry bohr^−1^. This ensures that the intermediate and final structures are close to their local minima and that all structural descriptors (coordination numbers, bond-angle distributions and tetrahedral order) are physically meaningful.

### Machine learning interatomic potential

The machine learning interatomic potential was trained with the MACE^65^ software v.0.3.15. The model is fine-tuned from the MACE-MH-1 foundation model^66^ using the ‘omat_pbe’ head on a combination of two sources of data. The density functional theory (DFT) data obtained from the quasi-static compression described in the section ‘Ab initio simulations’ containing 511 frames; and a set of temperature-driven compression computed using the MatterSim model^67^ (MatterSim-v1.0.0-1 M.pth) that has been trained on various HPHT DFT configurations, representing 6,416 configurations. We found that the inclusion of the latter source of data greatly stabilizes the fine-tuned model for high-temperature simulations. The energies of the isolated atomswere re-estimated for each data source (DFT and MatterSim) using the process outlined in appendix A in ref. ^66^.

## Online content

Any methods, additional references, Nature Portfolio reporting summaries, source data, extended data, supplementary information, acknowledgements, peer review information; details of author contributions and competing interests; and statements of data and code availability are available at 10.1038/s41586-026-10669-3.

## Supplementary information

Supplementary Video 1 MLIP simulation showing the evolution of the m-NG precursor at 1,500 K under increasing pressure. The *sp*^2^–*sp*^3^ rehybridization starts at 42 GPa. Colours: blue, *sp*^2^ carbon; green, *sp*^3^ carbon; red, hydrogen.

Supplementary Video 2 MLIP simulation showing the evolution of the m-NG precursor at 200 K under increasing pressure. The *sp*^2^–*sp*^3^ rehybridization starts at 60 GPa. Colours: blue, *sp*^2^ carbon; green, *sp*^3^ carbon; red, hydrogen.

Supplementary Video 3 MLIP simulation showing the evolution of the m-NG precursor at 0 K under increasing pressure. The *sp*^2^–*sp*^3^ rehybridization starts at 93 GPa. Colours: blue, *sp*^2^ carbon; green, *sp*^3^ carbon; red, hydrogen.

Supplementary Video 4 Ab initio simulation showing the evolution of the m-NG precursor at 0 K under increasing pressure. The *sp*^2^–*sp*^3^ rehybridization starts at 78 GPa. Colours: blue, *sp*^2^ carbon; green, *sp*^3^ carbon; red, hydrogen.

Peer Review File

## Data Availability

The data supporting the findings of this study are available in the paper and its Extended Data files, and also at Zenodo (10.5281/zenodo.20638474)^68^.

## Code availability

Ab initio simulations were performed with the Quantum ESPRESSO package (PWSCF v.6.7MaX 2024). Molecular dynamics simulations were performed with the LAMMPS code^69^. The PyMOL Molecular Graphics System, v.3.0.2, Schrödinger, was used to render the simulation snapshots. The custom Fortran codes used for nanoparticle construction and data analysis were developed for this specific study and are not packaged for general use. They are available from R.C.-H. upon reasonable request. The MACE code is available as open source at GitHub (https://github.com/ACEsuit/mace). The model is available at Zenodo (10.5281/zenodo.20638474)^68^.
