# Supplementary material for: Bottom-up synthesis of molecular nanodiamond from nanographene
Source: Nature. 2026 May 26;655(8121):102–8. doi: 10.1038/s41586-026-10669-3 (PMC13323094; doi:10.1038/s41586-026-10669-3)
Supplement: Supplementary file 5 — Peer Review File [file 41586_2026_10669_MOESM5_ESM.pdf]

nature portfolio

Peer Review File

Bottom-Up Synthesis of Molecular Nanodiamond from Nanographene

Corresponding Author: Professor Tanja Weil

This file contains all reviewer reports in order by version, followed by all author rebuttals in order by version.

Attachments originally included by the reviewers as part of their assessment can be found at the end of this file.

Version 0:

Reviewer comments:

Referee #1

(Remarks to the Author)
Liang et al. reported on an innovative bottom up synthesis of high crystalline monodisperse nanodiamonds from nanographene precursors. The reported results demonstrated the synthesis of monodispersed ND that could host SiV or GeV color centers. This is a very important achievement for the ND scientific community. The present manuscript is suitable for publication in Nature after the following revisions.

Introduction

« To date, NDs are produced by high-pressure high-temperature (HPHT) protocols or in detonation chambers" CVD approaches were also reported for the bottom up of ND:
Tallaire et al., ACS Appl. Nano Mater. 2019, 2, 5952–5962
De Feudis et al., Adv. Mater. Interfaces 2020, 7, 1901408

"vacuum annealing to activate the colour centres…" modify to: to create colour centres via vacancies diffusion

"Although NDs with nitrogen vacancy colour centres are commercially available, the broad variations in shape, size and amount and location of the colour centre greatly limit quantum sensing applications" Authors could add surface chemistry in this list of uncontrolled parameters.

Results

Fig 1, caption "doping molecules" This is not discussed in the text at this stage, please provide details.

"X-ray diffraction (XRD) spectrum…" XRD diffractogram (not spectrum)

Raman: the power density should be added to the experimental conditions. Indeed, nanodiamonds can be modified under laser illumination: Mermoux et al., J. Phys. Chem. C 2014, 118, 23415–23425
The provided Raman spectrum (extended data figure 1) is not so well resolved. Could the authors provide one focusing in the region of interest (800-2000 cm-1)? This will allow to better analyze the possible presence of a D band.

"Further, a broad peak at around 2900 cm−1, attributed to C-H bonds, suggests the existence of a significant amount of hydrogen atoms originating from the m-NG precursor."
Raman is not the most sensitive to C-H bonds. The hydrogen termination of ND could be well confirmed using FTIR with specific signatures (Saoudi et al., Carbon 202 (2023) 438–449).
What is the zeta potential of these ND dispersed in water? It should be positive in case of hydrogenated ND (same ref Saoudi et al.).

Authors should analyze more HRTEM pictures (Figure 2) to discuss the possible presence of sp2 carbon at the ND surface.

What means ED-XRD measurements ?

Notation for planes: 111 plane: (111) plane, idem (002); (111), (100) and higher-indexed facets, (311)

D220: to be introduced in the text

“This geometry is justified because high-temperature conditions favour the formation of spherical nanoparticles. “ In the proposed model, a spherical shape of ND was assumed. What about real m-ND? Did they exhibit facets? Such consideration should be included in the discussion.

What is the surface chemistry of SiV and GeV fNDs? It is an essential parameter that govern the optical properties of negatively charged SiV and GeV. This characterization (FTIR or XPS) should be included in the manuscript.

Typing errors:
Figure 5, caption diamond Raman peak

Referee #2

(Remarks to the Author)
The manuscript presents a bottom-up synthesis method for producing molecular nanodiamonds (m-NDs) by applying a HPHT process to mNG ($C_{96}H_{30}$). The authors demonstrate the ND formation with control over particle size and morphology, and report the successful incorporation of color defect centers such as SiV and GeV by simply adding dopant precursors to the reaction. The process is supported by a combination of
experimental techniques including SAXS, AFM, TEM, in situ ED-XRD, and complemented by ab initio calculations that provide insights into the growth mechanism and structure of the resulting NDs. The manuscript is well written. I believe the proposed synthesis method is interesting and has the potential to
impact the development of high-quality ultrasmall NDs for quantum applications. However, I believe several aspects of the manuscript require further clarification and more rigorous experimental validation. The main claims related to size control, material purity, and surface chemistry are not sufficiently supported by the
current data. Therefore, I cannot recommend the manuscript for publication in its current form.
1. The authors claim that the bottom-up HPHT process allows for improved control over ND size compared to conventional top-down techniques such as milling or detonation. However, the experimental data does not convincingly demonstrate this improvement. Fig 1c and Ext. Data Fig. 1f,i show particle size distributions measured by different techniques such as AFM, SAXS, and TEM. However the values of mean and standard deviation differ across these methods, and the manuscript does not discuss the reasons for this variation. A quantitative comparison of size dispersion, for instance by calculating and comparing the standard deviation-to-mean (std/mean) ratio for each method and sample, can help support the claim. It would also be helpful to compare these parameters to those of conventional NDs. In addition, the use of Raman spectroscopy to correlate particle size with peak position and linewidth, particularly the diamond band, could strengthen the discussion. Previous studies [Mermoux et al.
Diamond Rel. Mater. 87, 248–260, (2018)] have reported such dependencies, and including an alaysis like that reinforces the manuscript’s arguments about size control and crystallinity.
2. Although size is discussed extensively, the morphology of the NDs is not discussed. This is a significant omission, as shape can strongly influence properties such as fluorescence brightness and optical homogeneity. Conventional top-down NDs often exhibit rod- or plate-like shapes (e.g., Eldemrdash et al., Carbon 206, 268 (2023); Oshimi et al., ACS Nano 18, 35202 (2024); Haotian et al., ACS Nano 17, 16491 (2023)). If the present synthesis method truly enables morphological uniformity, this would be an important advantage, but the manuscript does not present data to demonstrate this. In addition, the TEM images provided have relatively low magnification (e.g., 20 nm scale bars), which is not sufficient for assessing particle shape or observing lattice patterns. Given that the manuscript refers to HR-TEM, higher-resolution images should be provided to show lattice structures extending to the surface of particles. Such data could also reveal the presence of sp^2-rich surface layers or graphitic contamination. Additionally, performing statistical shape analysis on a representative number of particles is valuable to support the claim of morphological control.
3. The presence of GR1 centers shown in Fig 5c may indicate lattice defects or imperfections. A more discussion of GR1 centers, and ideally, further data should be included to assess the defect situations in these NDs more comprehensively. Further, EPR measurements is useful in this context, as they can quantify vacancy and impurity defects. Since the manuscript emphasizes the scalability of the synthesis method, it is reasonable to expect that sufficient sample quantities can be produced for EPR analysis.
4. For ultrasmall NDs, bulk-based techniques such as Raman and XRD are not sufficient to fully characterize the NDs. Electronic structure and bonding should be studied using more site-specific or surface-sensitive methods such as EELS or XAS. As many properties of ultrasmall NDs deviated from the bulk diamond
crystals, the site-specific observations on the electronic structure will unambiguously address exact

material situations of the present ND samples [Chang et al., ACS Nano 16, 8513 (2022). Ref. 23 in the present manuscript]. The authors need to perform EELS or XAS for their samples to fully confirm the acquisition of high quality NDs.
5. The NDs containing SiV and GeV centers have significantly larger sizes (~30 nm) than the undoped m- NDs (~3 nm). This raises an important question about whether doping inherently leads to larger crystal growth, or whether it reflects an intentional or uncontrollable change in synthesis conditions. Since size and defect properties are central to the paper, a discussion on the cause of this difference is needed.
Also, the above thorough study on the size dependence in combination with this defect-included NDs can strengthen the

paper. Further, the applicability of the proposed [H]/[C] ratio model to doped NDs is not explained. Does this model still predict or control the size in the doped case? If not, why? The authors should clarify whether and how their theoretical framework can be extended to describe dopant-containing systems. If the size increase is unavoidable due to the requirements of incorporating SiV or GeV centers, this should be stated and justified clearly.
6. The strain in NDs can significantly affect the optical properties of color centers. For SiV centers, in particular, the width and distribution of ZPL can be used as an indicator of strain. The manuscript can be improved by analyzing the ZPL linewidths and comparing them with previous studies, such as Lindner et al. (New J. Phys. 20, 115002, 2018. Ref. 40 in the present manuscript), which relate optical line broadening to local strain fields through ab initio modeling. Providing this type of analysis would not only strengthen the interpretation of defect quality but also highlight the quantum optical properties.
7. The authors describe a hydrogenated ND surface in their theoretical model, but it is unclear whether the synthesized particles are actually hydrogen-terminated after HPHT processing. The manuscript does not describe the collection process or mention whether any surface cleaning or termination steps were applied after the synthesis. Since the surface strongly influences both optical properties and chemical stability, details on this point are essential. IR spectroscopy and XPS are necessary to characterize surface terminations and functional groups. These measurements would also help clarify how well the actual material matches the model assumptions.

Minor Comments

In the abstract, the term “superphenalene” is used, while the title refers to “nanographene.” It is better to clarify this terminology, since coronene, HBC, and m-NG are actually used in the experiments.

Line 221: “Fig. 4d” “Fig. 4e.”

In Ext. Data Fig. and captions, notations such as “(a+b)” are confusing and should be revised to specify each panel explicitly.

The use of labels like “Gottingen” and “MPIP” should be avoided or replaced with more meaningful ids with captions. And I can’t understand what these four NDs mean.

Additional note. I couldn't find a vide for extended data, unfortunately.

Referee #3

(Remarks to the Author)
The study “Bottom-up Synthesis of Molecular and Fluorescent Nanodiamonds from Nanographene” presents a novel synthesis method for hard-to-obtain nearly-monodisperse nanodiamonds, potential hosts for color centers. It is supported by impressive experimental data. The precursor is ‘molecular nano-graphene’, m-NG: C96H30 and C42H18. At conditions (1200 C, 13 GPa) close to those in making diamond phases, a complete conversion of m-NG into nanodiamonds m-ND is achieved, with very narrow size distribution, and negligible amount of sp^2-carbon (absence of G bands). Presented results are quite remarkable and deserve publication. However, the mechanistic explanation, theoretical models presently employed, are far inferior to the experimental findings claimed, significantly diminishing the work’s rigor, insufficient for publication in Nature in present form. The specific critique to be addressed in a major revision is below.
1) The phase diagram in Fig. 1, borrowed from bulk graphite studies, is not fully appropriate for hydrogenated molecular precursors. A discussion appearing perhaps more relevant to this work, with chemically induced phase transitions, e.g. [10.1021/jz5007912] see Fig 7, also [10.1002/smll.202004782] Figs. 2-3, is needed to contextualize the mechanism. Even if all available H originates only from those C_xH_y edges, it may be enough to promote the ‘basal plane’ chemical activation sp2-->sp3, and also explain why pressure needed in experiments appears tenfold lower than suggested here models predict.
1a) Indeed, hard to be convinced that the shown atomistic simulations adequately represent the formation process, being entirely focused on increased pressure 155 GPa ramped up 10 times above the experimental 12-13 GPa; in fact the lowest simulated value 13.8 GPa is near the topmost in experiments.
2) A technicality, but consequential: The authors state that molecular structures under pressure did not converge, terminating calculations at forces < 0.2 Ry/bohr, or 5 eV/A, obviously a very large force! This is either a typo or a serious flaw in describing/explaining the process. Forces of this magnitude (enough to break strong covalent bond) indicate unoptimized structures far from the energy minimum, rendering any parameters derived from such calculations (e.g., coordination numbers, bond angles) physically meaningless. Then the claim of a “direct transition” from m-NG to m-ND lacks theoretical, mechanistic support. Consider possibilities (1) above and (4) below?
3) Furthermore, the temperature clearly plays a significant role in the formation process but seems completely ignored in simulations, performed calculations being only at T = 0.
4) The ab initio model in Fig. 4a-c assumes infinite molecular stacks under not very realistic periodic conditions, ignoring T-effects and H-engagement. This poorly approximates the experimental system. If ab initio methods cannot even converge (not to mention limiting simulation size and time), semi-empirical potentials readily available for C-H systems (AIREBO, ReaxFF, or recent machine-learning like DeepMD) could be employed to model polycrystalline nucleation from molecular CxHy precursors. The current simulations mostly fail to bridge theory and experiment.
5) The proposed spherical morphology is at odds with the triangular geometry of m-NG. No mechanism is offered to explain how a m-NG layered precursor transforms into isotropic nanoparticles.
6) The correlation of the size distribution with the H/C ratio is logical and can be experimentally tested/proven by the use of H42H18 (already used in some parts of the manuscript) as its H/C ~ 0.42 should correspond to nanodiamond size of ~2 nm, distinct from 3 nm for C96H30, H/C ~ 0.31.
7) No explanation is provided on increased diamond size formed from smaller molecules. Furthermore, no comment was made on a significant increase in nanodiamond size with the addition of Si and Ge dopants. SiV-NDs (29±8 nm) are tenfold

(~103 in volume?) larger than m-NDs (3.1±0.6 nm), yet the text offers no explanation. Does doping alter nucleation kinetics? This oversight weakens the mechanistic narrative.
8) While the main achievement here is m-NDs synthesis, the main value is in m-ND functionality as the hosts-particles for SiV^- and GeV^- defects, with demonstrated photoluminescence (PL) of zero-phonon line (ZPL) emission near 738 nm and 603 nm. These wavelengths are consistent with previously reported values for the same defects in bulk diamond. However, while these ZPL positions may be familiar to researchers working specifically on diamond defects, they are not widely known outside this field. The authors should therefore cite relevant literature from bulk diamond studies to support this identification.
9) Additionally, the authors assert that the synthesized defects are in a negative charge state, which is not obvious or granted. They could comment on the origin of this charge, identify possible donor species responsible for providing the extra electron. Evaluating the corresponding CTL (charge transition level) and its position w.r.t. “Fermi level” may also be expected. This is particularly relevant given that their synthesis procedure does not appear to introduce obvious contaminants or impurities.
***

Version 1:

Reviewer comments:

Referee #1

(Remarks to the Author)
Authors have taken into account most of the previous comments and the overall quality of the manuscript was greatly improved. Nevertheless, several comments need to be considered before the publication of the manuscript.

1) Abstract, Introduction and Discussion
« Here we show that well-defined, hydrogen-terminated molecular nanographenes serve as chemically confined precursors for high-pressure, high-temperature synthesis of ultrasmall (3–4 nm), monodisperse and highly crystalline molecular nanodiamonds (m-NDs) containing a hydrogen-terminated surface with only a single sp^2 surface reconstruction”
“FTIR spectra (Extended Data Fig. 3a) reveal distinct C–H stretching modes centred around 2830 cm^{-1} and, together with the positive zeta potential of +8.2 ± 0.6 mV (Extended Data Fig. 3f), confirm hydrogen termination of m-ND”
According to the FTIR spectra, very weak C-H signature can be seen compared to spectra of hydrogenated nanodiamonds (Miliaieva et al. Nanoscale Advances 2023; Saoudi et al. Carbon 2023). Moreover, the related zeta potential exhibits a low positive value (less than 10 mV). Other surface termination of nanodiamonds can lead to positive zeta potential (sp2 terminated, OH- terminated,…). These FTIR / zeta results underline a partial coverage by C-H bonds, such bonds are not the major part of the functional groups. Authors cannot talk about a hydrogen-terminated surface. This statement should be modified in the abstract, the introduction and the discussion.

2) “A single sp2 surface reconstruction is visible, appearing partially detached in some regions and giving rise to slightly wavy edges (Fig. 2c)”
This sp2 reconstruction should lead to a component located at - 1eV from the sp3 one in the C1s XPS (see references on hydrogenated nanodiamonds in comment 1). It has not been considered by authors in their provided fits (Extended Data Fig. 3). Authors should consider this and provide the quantitative analysis of the C1s fit (% of area for the carbon binding states) for m-ND and m-NDOx.

3) Some values should be according to the resolution or the measurement accuracy:
“characteristic diamond peak at 1329.3 cm^{-1}” 1329 cm-1, according to the spectral resolution
“+8.2 ± 0.6 mV » 8 ± 1 mV according to the measurement accuracy, apply to other values:
“−15.4 ± 0.5 mV »
« +10.7 ± 0.7 mV to −20.9 ± 0.5 mV »

4) All references should be checked (all co-authors listed)

Referee #2

(Remarks to the Author)
I think the authors have beautifully answered all the questions raised, and now the data are very convincing that ultrasmall, highly size- and shape-uniform nanodiamonds were synthesized in a bottom-up approach. I therefore recommend publication of the manuscript. Other than the scientific issues, I have some further points that would improve the quality of the presentation.

1. The HR-TEM image can be magnified to this level as follows (attached) to show the surface sp^2 layer more clearly. Also, a pointing arrow or something similar could help readers understand it.

2. This diamond symbol in Fig. 2b-e perhaps indicates diamond data, but it is hard to catch the information. I think these marks are not necessary, or the meaning should be written in the captions.

3. The newly measured EELS data (Extended Data Fig. 4) are striking and very convincing for the formation of diamond and

the sp^2 surface structure. I think these data could be included in the main figures as part of Fig. 2. However, I leave this to the authors' decision.

Referee #3

(Remarks to the Author)
In this second view i do nnaturally ot follow all the A-H items recommended.
I think the authors have addressed most of concerns thoroughly, to my satisfaction at least.
1) Ref 41 is a good one, but I bet must also include the book title, as "…in 'Nucleation in Condensed Matter', pp 19-54…' Without it just a chapter title alone "The Classical Theory" reads hollow out of the context (theory of what?). Format this ref properly. Alos in ref 40 pages ## seem missing?
2) Understandably one of the central surprises here remains why the needed pressure appears so much lower than would be expected in "common" graphite diamond transformation, the authors may benefit from seeing the phase diagram Fig. 1 of Nano Lett. dx.doi.org/10.1021/nl403938g If space permits it could be valuable ref to add for the readers too.
Overall, the work main result is quite interesting and it can be published, with minor additions/improvements (1-2) above if paper goes for another round of quick revision.

Version 2:

Reviewer comments:

Referee #1

(Remarks to the Author)
Authors have very well answered to my last comments. This beautiful study can now be published in Nature.

Open Access This Peer Review File is licensed under a Creative Commons Attribution 4.0 International License, which permits use, sharing, adaptation, distribution and reproduction in any medium or format, as long as you give appropriate credit to the original author(s) and the source, provide a link to the Creative Commons license, and indicate if changes were made.
In cases where reviewers are anonymous, credit should be given to 'Anonymous Referee' and the source.
The images or other third party material in this Peer Review File are included in the article's Creative Commons license, unless indicated otherwise in a credit line to the material. If material is not included in the article's Creative Commons license and your intended use is not permitted by statutory regulation or exceeds the permitted use, you will need to obtain permission directly from the copyright holder.
To view a copy of this license, visit https://creativecommons.org/licenses/by/4.0/

Referee #1: Experiment

Liang et al. reported on an innovative bottom-up synthesis of high crystalline monodisperse nanodiamonds from nanographene precursors. The reported results demonstrated the synthesis of monodispersed ND that could host SiV or GeV color centers. This is a very important achievement for the ND scientific community. The present manuscript is suitable for publication in Nature after the following revisions.

We thank the reviewer for the careful and thoughtful evaluation of our manuscript and for recognising the significance of our bottom-up approach to synthesising monodisperse, highly crystalline nanodiamonds. We greatly appreciate the reviewer's constructive comments and address each point below.

Q1. "To date, NDs are produced by high-pressure high-temperature (HPHT) protocols or in detonation chambers" CVD approaches were also reported for the bottom up of ND: Tallaire et al., ACS Appl. Nano Mater. 2019, 2, 5952−5962; De Feudis et al., Adv. Mater. Interfaces 2020, 7, 1901408.

We thank the reviewer for highlighting these important CVD-based nanodiamond studies. We have now incorporated both suggested references (Tallaire et al.[1]; De Feudis et al.[2]) and revised the introduction to explicitly acknowledge CVD as an established bottom-up route.

Changes to the manuscript*:* *Page 3, Lines 59–61 (Introduction): "High-pressure, high-temperature (HPHT) methods[14–16], chemical vapour deposition[17,18] and detonation synthesis[19] generate heterogeneous materials that vary widely in size, morphology and impurity content."*

Q2: "vacuum annealing to activate the colour centres..." modify to: to create colour centres via vacancies diffusion

We appreciate the reviewer's suggestion, which indeed provides a more accurate mechanistic description. We have revised the wording accordingly.

Changes to the manuscript: *Page 3, Lines 73–74 (Introduction): "…vacuum annealing to create colour centres via vacancy diffusion, and high-energy ball milling…"*

Q3 "Although NDs with nitrogen vacancy colour centres are commercially available, the broad variations in shape, size and amount and location of the colour centre greatly limit quantum sensing applications" Authors could add surface chemistry in this list of uncontrolled parameters.

We thank the reviewer for highlighting this important aspect. Surface functionalisation is indeed a critical and often uncontrolled parameter, and we have now incorporated it explicitly.

Changes to the manuscript: *Page 3-4, Lines 74–76 (Introduction): "These processes produce particles with broad variations in defect concentration, size, morphology and surface chemistry, which limits performance in quantum sensing and imaging[27]."*

Q4: Fig 1, caption "doping molecules" This is not discussed in the text at this stage, please provide details.

We thank the reviewer for noting the lack of clarity regarding "doping molecules." We have expanded the figure caption to specify the dopant precursors used in the two-component reaction.

Changes to the manuscript: *Page 5, Lines 105–107 (Fig. 1 caption): "…while a two-component mixture of m-NG with tetrakis(trimethylsilyl)silane (Si-) or tetraphenylgermane (Ge-) yields fluorescent nanodiamonds…"*

Q5: "X-ray diffraction (XRD) spectrum..." XRD diffractogram (not spectrum)

We appreciate the reviewer's attention to correct terminology. All instances of "XRD spectrum" have been revised to "XRD diffractogram."

Changes to the manuscript: *All occurrences of "XRD spectrum" have been corrected to "XRD diffractogram." (Results sections)*

Q6: Raman: the power density should be added to the experimental conditions. Indeed, nanodiamonds can be modified under laser illumination: Mermoux et al., J. Phys. Chem. C 2014, 118, 23415−23425

We thank the reviewer for noting the important effect of laser power density on nanodiamonds. We re-measured the Raman spectra using different excitation power from 0.1 mW to 3 mW and found a significant downshift of the Raman peak with increasing power (see Supportive Figure), which is consistent with the laser-induced modification of nanodiamonds (Mermoux et al.[3],). Thus, we present the Raman data obtained with an excitation power of 0.1 mW, corresponding to a power density of around 130 $\mu W\ \mu m^{-2}$. The power density at sample is estimated to be below 10 $\mu W\ \mu m^{-2}$. We now state the power density used in all m-ND Raman measurements to avoid significant sample modification.

Changes to the manuscript: *Page 21, Lines 457–458 (Methods): "Raman spectra of m-NDs were recorded using a laser power density of <150 $\mu W\ \mu m^{-2}$ to avoid laser-induced modifications of the nanodiamonds."*

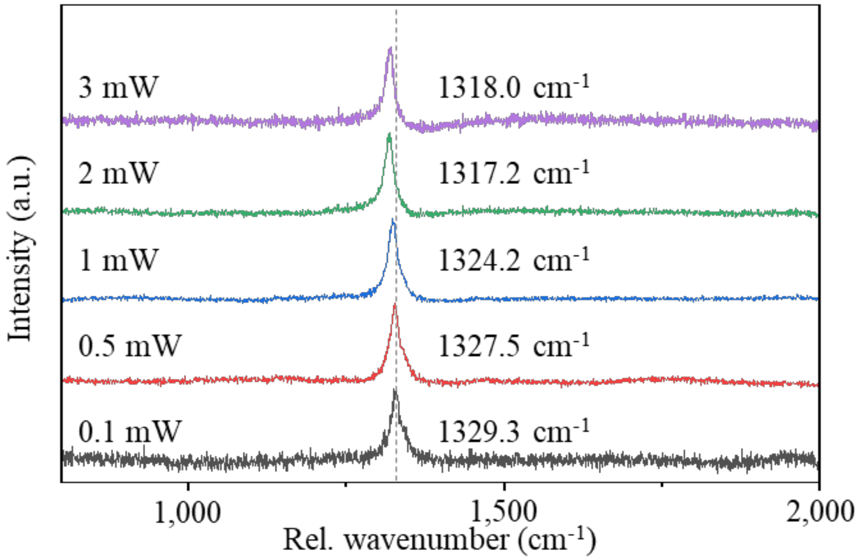

Supportive Figure: Raman analyses of m-ND excited at different laser power. The Raman peak of diamond downshifts with increasing laser power. The excitation wavelength is 532 nm.

Q7: The provided Raman spectrum (extended data figure 1) is not so well resolved. Could the authors provide one focusing in the region of interest (800-2000 cm-1)? This will allow to better analyze the possible presence of a D band.

We appreciate the reviewer's suggestion. We re-measured the Raman spectra and now provide high-resolution data (800–2000 cm^{-1}) for m-ND and HPHT-treated graphite. The m-ND spectrum shows a diamond peak at 1329.3 cm^{-1} and no detectable band between 1400 – 1800 cm^{-1} in any nanodiamond sample even though we used a green laser (532 nm). Extended Data Fig. 1d now presents the requested high-resolution spectra.

Changes to the manuscript: *Page 6, Lines 118-121 (Results): "A high-resolution Raman spectrum (800–2000 cm^{-1}) displays the characteristic diamond peak at 1329.3 cm^{-1} and no detectable G band (~1580 cm^{-1}), indicating the absence of graphitic carbon, unlike typical detonation nanodiamonds[34] (Extended Data Fig. 1d)."*

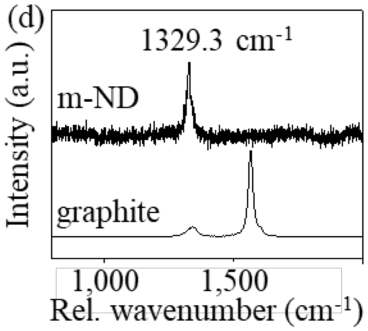

Extended Data Fig. 1 d: Raman spectra of untreated m-ND and graphite after HPHT (graphite).

Q8: "Further, a broad peak at around 2900 cm−1, attributed to C-H bonds, suggests the existence of a significant amount of hydrogen atoms originating from the m-NG precursor." Raman is not the most sensitive to C-H bonds. The hydrogen termination of ND could be well confirmed using FTIR with specific signatures (Saoudi et al., Carbon 202 (2023) 438–449).

We thank the reviewer for pointing out that Raman spectroscopy alone is not optimal for assessing C–H bonds. In response, we performed FTIR measurements on both untreated and acid-treated nanodiamonds. Untreated samples show clear C–H stretching modes and a characteristic dip at ~1331 cm^{-1} (*) associated with Fano-type interference in hydrogenated nanodiamonds (Saoudi et al.[4]; Kudryavtsev et al.[5]; Ekimov et al.[6]). After acid treatment, C=O signatures appear, consistent with surface oxidation. Zeta-potential measurements further support the transition from hydrogen-terminated (positive) to oxidised (negative) surfaces. Extended Data Fig. 3 now includes XPS, FTIR, and zeta-potential data for all samples.

Changes to the manuscript: *Page 6, Lines 121-125 (Results): "FTIR spectra (Extended Data Fig. 3a) reveal distinct C–H stretching modes centred around 2830 cm^{-1} and, together with the positive zeta potential of +8.2 ± 0.6 mV (Extended Data Fig. 3f), confirm hydrogen termination of*

m-NDs[35]. Acid treatment produces oxidised m-NDs (m-ND$_{ox}$), with characteristic C=O features in XPS (Extended Data Fig. 3b–e), FTIR signatures and a negative zeta potential of −15.4 ± 0.5 mV (Extended Data Fig. 3f)."

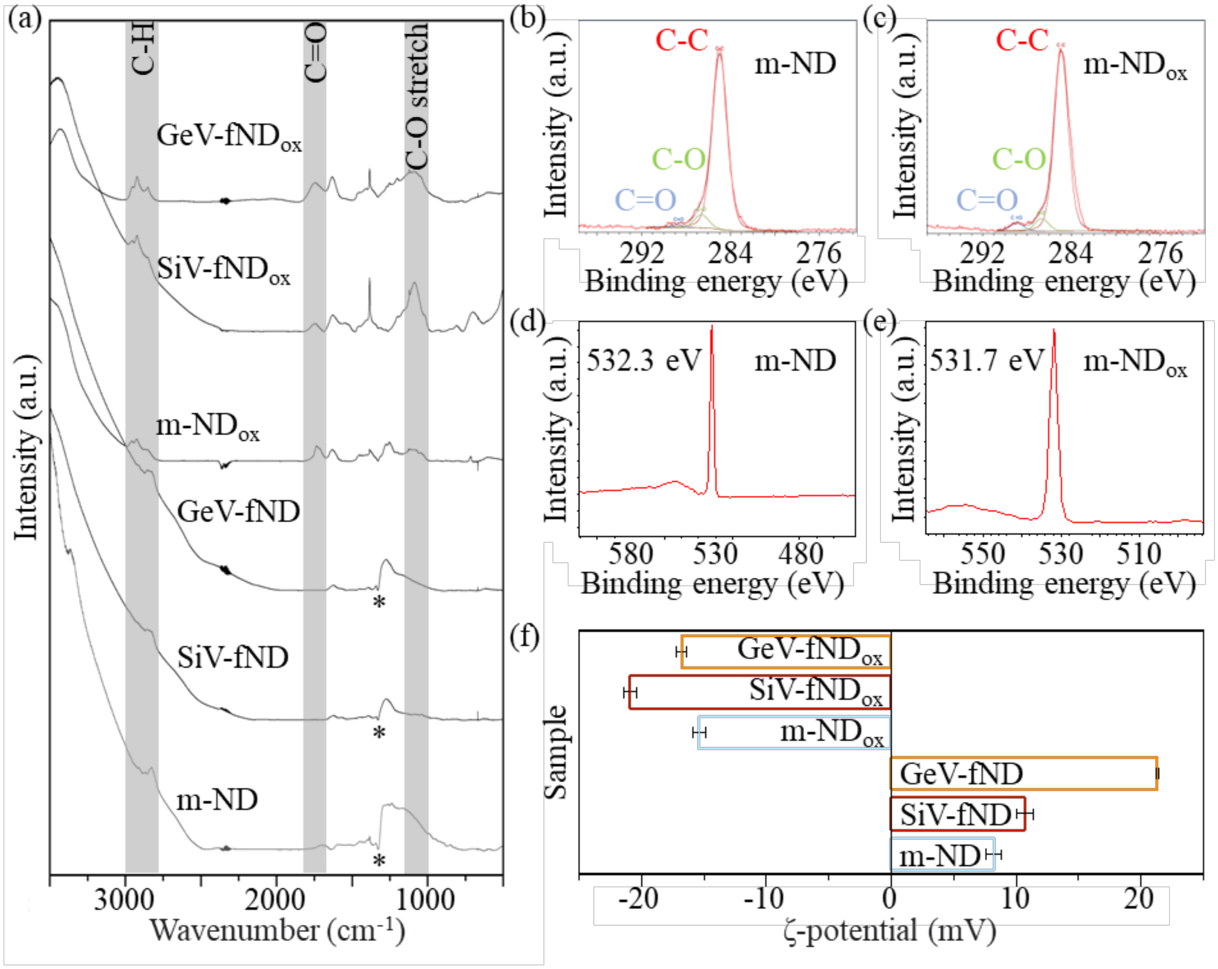

Extended Data Fig. 3: Surface characterisation of Nanodiamonds. a, FTIR spectra of untreated nanodiamonds (m-ND, SiV-fND and GeV-fND) and treated nanodiamonds (m-ND$_{ox}$, SiV-fND$_{ox}$ and GeV-fND$_{ox}$). The dip at ~1331 cm^{-1} (*) is associated with Fano-type interference in hydrogenated nanodiamonds[4]. **b**, XPS spectrum of untreated m-ND focusing on the C-region. **c**, XPS spectrum of m-ND$_{ox}$ focusing on the C-region showing increased C=O species. **d**, XPS spectra of untreated m-ND and m-ND$_{ox}$**e**, focusing on the O-region **f**, Zeta potential of the untreated (m-ND, SiV-fND, and GeV-fND) and the acid treated samples (m-ND$_{ox}$, SiV-fND$_{ox}$ and GeV-fND$_{ox}$). The positive zeta potentials are indicative of hydrogenated ND surfaces, while the negative zeta potentials reflect surface oxidation.

Q9: What is the zeta potential of these ND dispersed in water? It should be positive in case of hydrogenated ND (same ref Saoudi et al.).

We thank the reviewer for raising this important point. To directly assess the surface termination of our nanodiamonds in aqueous dispersion, we measured the zeta potential of all samples (m-ND, SiV-fND, GeV-fND) before and after acid treatment. As expected for hydrogen-terminated

diamond surfaces (Saoudi et al.[4]), all untreated samples exhibit positive zeta potentials, whereas oxidised samples show negative values. Combined analysis from FTIR, XPS, and zeta potential thus consistently supports hydrogen termination in the crude nanodiamonds and surface oxidation after acid treatment.

Supporting data included:

- FTIR: C–H stretching in untreated samples; C=O signals after oxidation
- XPS: transition from C–H dominant to C=O-rich surfaces
- Zeta potentials:
  - untreated: positive (hydrogen-terminated)
  - acid-treated: negative (oxidised)
- All data shown in response to Q8 (see Extended Data Fig. 3).

Changes to the manuscript: *Page 6, line 121-125 (Results): "FTIR spectra (Extended Data Fig. 3a) reveal distinct C–H stretching modes centred around 2830 cm $^{-1}$ and, together with the positive zeta potential of +8.2 ± 0.6 mV (Extended Data Fig. 3f), confirm hydrogen termination of m-NDs[35]. Acid treatment produces oxidised m-NDs (m-ND$_{ox}$), with characteristic C=O features in XPS (Extended Data Fig. 3b–e), FTIR signatures and a negative zeta potential of −15.4 ± 0.5 mV (Extended Data Fig. 3f).*"

Q10: Authors should analyze more HRTEM pictures (Figure 2) to discuss the possible presence of sp2 carbon at the ND surface.

We thank the reviewer for this valuable suggestion. In response, we performed an extended set of high-resolution TEM (HRTEM) analyses using a Cs-double-corrected (S)TEM on both crude and acid-treated m-NDs. The new micrographs, two of which are included in Fig. 2c and Extended Data Fig. 1c, confirm that the particles are highly crystalline and uniformly sized (4 ± 1.4 nm, updated Fig. 2 e). Importantly, all particles exhibit a single surface sp^2 reconstruction distinct from the diamond core. Similar surface features have been reported previously (Chang et al.[7]; Raty et al.[8]) and are attributed to surface relaxation of the (111) facets, causing the outermost layer to shift slightly outward and adopt a curved, fullerene-like arrangement. This interpretation is consistent with our intensity analysis, which reveals that the interplanar spacing at the surface is 36 ± 9% larger than in the diamond interior.

To determine the hybridisation state of this surface reconstruction, we performed spatially resolved EELS spectral imaging. EELS spectra display the well-defined σ* features expected for crystalline diamond at 293, 298.5, and 306 eV, corresponding to 1s → σ* transitions. A small π* peak at 285.5 eV is detected in all samples and becomes more pronounced at the surface. Principal component analysis (PCA) and k-means clustering allow separation of the sp^2 and sp^3 contributions; the sp^2 signal is confined to a single surface reconstruction, with no evidence for thicker graphitic shells. Identical behaviour is observed in both m-ND and m-ND$_{ox}$. This combined HRTEM and EELS analysis demonstrates that the nanodiamonds synthesized via our bottom-up approach possess exceptionally clean surfaces, with only a single sp^2 surface reconstruction, much lower than values typically reported for nanodiamonds of similar size (e.g., Duan et al.[9]). The structural purity achieved underscores the advantage of the molecular precursor strategy employed here.

- HRTEM micrographs of m-ND (Fig. 2 c) and m-ND$_{ox}$ (Extended Data Fig. 1c)

- Extended Data Fig. 4: full EELS spectral maps, PCA clustering, surface/bulk spectral reconstructions
- References: Chang 2022[7]; Raty 2003[8]; Barnard 2003[10]; Duan 2018[9]

Changes to the manuscript: *Page 6, Lines 128-135 (Results): "HRTEM images of both crude (Fig. 2c) and acid-treated (Extended Data Fig. 1 c) m-NDs reveal highly crystalline, faceted, approximately equiaxed nanoparticles. A single sp^2 surface reconstruction is visible, appearing partially detached in some regions and giving rise to slightly wavy edges (Fig. 2c). EELS spectral imaging (Extended Data Fig. 4) confirms that this reconstruction is sp^2-rich, and the presence of such a thin reconstruction (<0.5 nm) is notable compared with the higher sp^2 fractions typically reported for nanodiamonds of similar size[34]. These combined results indicate that m-NDs possess minimal graphitic carbon and high structural purity."*

(c)

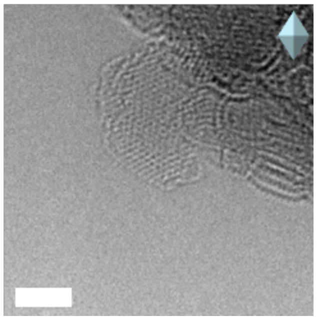

Fig. 2 c: HRTEM micrograph of m-ND, evidencing high crystallinity and a single sp^2 surface reconstruction. Scale bar, 2 nm.

(c)

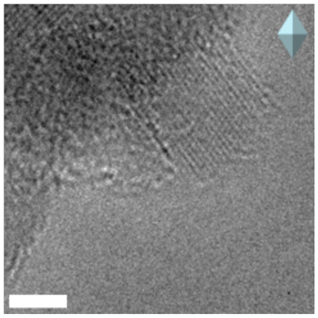

Extended Data Fig. 1 c: HRTEM micrograph of m-ND_{ox}, evidencing high crystallinity and a single sp^2 surface reconstruction. Scale bar, 2 nm.

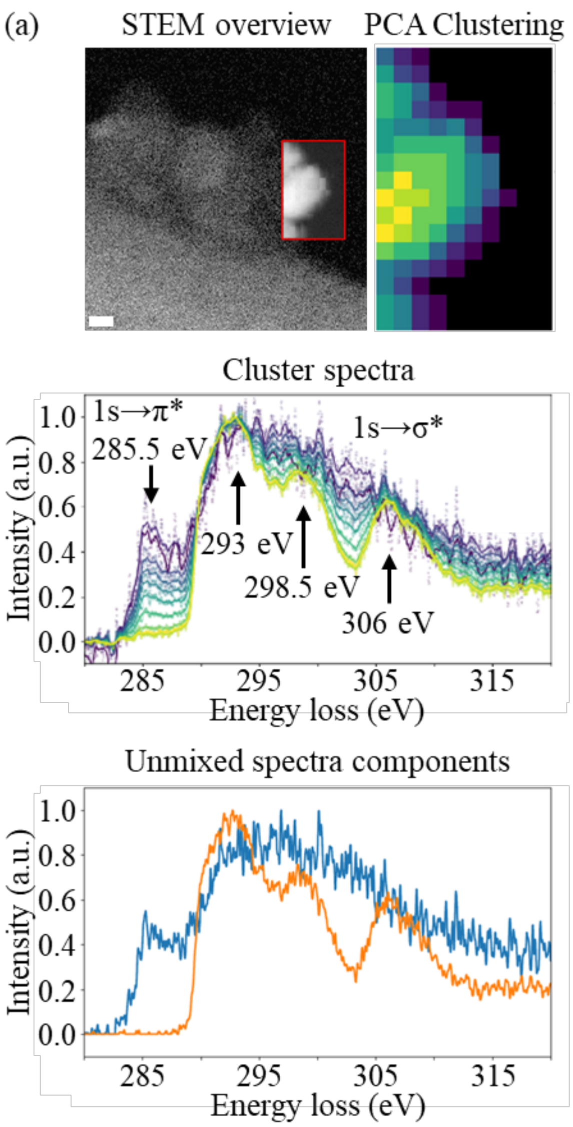

Extended Data Fig. 4: EELS surface characterization of m-ND particles. a, EELS analysis of m-ND using a 0.5 nm steps Spectral Image (SI). The pixels in the SI were clustered in 10 regions (and an additional vacuum one) using PCA and k-means clustering on the EELS spectra. The average spectra of the clusters, shown normalised, were then used to reconstruct the EELS spectra of the surface (blue) and bulk (orange) components. The presence of the bulk component in the outermost region indicates that sp^2-layer thickness does not exceed 0.5 nm. **b**, the same analysis was performed on m-ND_{ox}, showing qualitatively the same results. Scalebars are 2 nm.

Q11: What means ED-XRD measurements?

We thank the reviewer for highlighting this missing definition. We now introduce the full term in the manuscript.

Changes to the manuscript: *Page 8, Lines 162-163 (Results): "…we performed in situ energy-dispersive X-ray diffraction (ED-XRD) at beamline P61B (DESY, Hamburg, Germany)[37]…"*

Q12: Notation for planes: 111 plane: (111) plane, idem (002); (111), (100) and higher-indexed facets, (311)

We thank the reviewer for pointing out these inconsistencies. All crystallographic plane notations have been corrected throughout the text and figures.

Changes to the manuscript: *All plane indices are now consistently written as (111), (002), (220), (311), etc. (Figures and Results section updated)*

Q13: D220: to be introduced in the text

We thank the reviewer for noting this omission. The diamond (220) reflection is now explicitly introduced at its first appearance in the text.

Changes to the manuscript: *Page 9, Lines 183-186 (Results), "The appearance of the diamond (220) reflection (D(220)) in the m-NG sample at 13 GPa (Fig. 3d) shows that nanodiamond formation begins below 1,200 °C, preceding the disappearance of the m-NG (002) feature. "*

Q14: "This geometry is justified because high-temperature conditions favour the formation of spherical nanoparticles. " In the proposed model, a spherical shape of ND was assumed. What about real m-ND? Did they exhibit facets? Such consideration should be included in the discussion.

We thank the reviewer for this important point. Although spherical geometry is a reasonable approximation under high-temperature conditions, HRTEM shows that m-NDs are not perfectly spherical. The geometry analysis reveals that m-NDs display an approximately equiaxed aspect ratio (diameter/height = 1.2) with comparable lateral dimensions and heights derived from TEM and AFM (Fig. 2e; Extended Data Fig. 1g-i). They exhibit distinct facets and a single surface sp^2 reconstruction. We now clarify that the spherical assumption captures overall thermodynamic/kinetic trends but does not include the experimentally observed faceting.

Changes to the manuscript: *Page 6, Lines 128-131 (Results): "HRTEM images of both crude (Fig. 2c) and acid-treated (Extended Data Fig. 1 c) m-NDs reveal highly crystalline, faceted, approximately equiaxed nanoparticles. A single sp^2 surface reconstruction is visible, appearing partially detached in some regions and giving rise to slightly wavy edges (Fig. 2c)."*

Page 7, Lines 144-146 (Results): "Geometry analysis reveals that milled NDs are disk-like (diameter/height ≈ 4.9), while m-NDs are approximately equiaxed (diameter/height ≈ 1.2) based on TEM and AFM, further confirming more uniform morphology of m-NDs."

Page 11, Lines 221-223 "Calculations of assumed spherical diamond clusters give an intersection at d ≈ 3 nm with r = 0.3125, in agreement with the measured diameter of 4.0 ± 1.4 nm (Fig. 4f and Fig. 2e)."

Q15: What is the surface chemistry of SiV and GeV fNDs? It is an essential parameter that govern the optical properties of negatively charged SiV and GeV. This characterization (FTIR or XPS) should be included in the manuscript.

We thank the reviewer for this excellent suggestion. Because the charge state and optical stability of group-IV colour centres are strongly influenced by surface termination, we have now performed a full set of surface-analysis measurements, FTIR, and zeta potential, on both crude and acid-treated SiV-fNDs and GeV-fNDs.

Surface chemistry of SiV-fNDs:

- FTIR spectra show C–H stretching modes (~2830 cm^{-1}) in untreated samples, consistent with hydrogen termination.
- Upon acid treatment, FTIR reveals an increase in C=O vibrations, confirming oxidation.
- Zeta potential shifts from +10.7 ± 0.7 mV (SiV-fND) to −20.9 ± 0.5 mV (SiV-fND_{ox}) (Extended Data Fig. 3f).

Surface chemistry of GeV-fNDs:

- FTIR also shows predominant C–H termination in untreated samples and the emergence of C=O modes after oxidation.
- Zeta potential shifts from +21.3 ± 1.0 mV (GeV-fND) to −16.8 ± 0.4 mV (GeV-fND_{ox}).
- FTIR trends are fully consistent across the full sample set.

These data have been integrated into the manuscript and the corresponding spectra have been added to Extended Data Fig. 3. Extended Data Fig. 3a now includes FTIR spectra for all crude and acid-treated group-IV-doped nanodiamonds.

Changes to the manuscript: *Page 15, Lines 318-321 (Results): "FTIR indicates C–H termination before acid treatment and increased C=O rich surfaces afterwards (Extended Data Fig. 3a), with zeta potential changing from +10.7 ± 0.7 mV to −20.9 ± 0.5 mV (Extended Data Fig. 3f), confirming the expected surface chemistry of crude and oxidised fNDs."*

Page 16, Lines 339-340 (Results): "FTIR and zeta-potential trends mirror those of SiV-fNDs (Extended Data Fig. 3a, f)."

Q15: Typing errors: Figure 5, caption diamond Raman peak

We thank the reviewer for pointing out this typo, and have corrected the caption accordingly.

Changes to the manuscript: "…*showing the diamond Raman…" (Results, Page 17, Line 356 and Page 18, Line 361).*

References:

1. Tallaire, A. *et al.* Synthesis of Loose Nanodiamonds Containing Nitrogen-Vacancy Centers for Magnetic and Thermal Sensing. *ACS Appl. Nano Mater.* **2**, 5952–5962 (2019).
2. De Feudis, M. *et al.* Large-Scale Fabrication of Highly Emissive Nanodiamonds by Chemical Vapor Deposition with Controlled Doping by SiV and GeV Centers from a Solid Source. *Adv. Mater. Interfaces* **7**, (2020).
3. Mermoux, M., Crisci, A., Petit, T., Girard, H. A. & Arnault, J.-C. Surface Modifications of Detonation Nanodiamonds Probed by Multiwavelength Raman Spectroscopy. *J. Phys.*

Chem. C **118**, 23415–23425 (2014).

4. Saoudi, L. *et al.* Colloidal stability over months of highly crystalline high-pressure high-temperature hydrogenated nanodiamonds in water. *Carbon N. Y.* **202**, 438–449 (2023).

5. Kudryavtsev, O. S. *et al.* Fano-type Effect in Hydrogen-Terminated Pure Nanodiamond. *Nano Lett.* **22**, 2589–2594 (2022).

6. Ekimov, E. *et al.* Size-Dependent Thermal Stability and Optical Properties of Ultra-Small Nanodiamonds Synthesized under High Pressure. *Nanomaterials* **12**, 351 (2022).

7. Chang, S. L. Y., Reineck, P., Krueger, A. & Mochalin, V. N. Ultrasmall Nanodiamonds: Perspectives and Questions. *ACS Nano* **16**, 8513–8524 (2022).

8. Raty, J.-Y., Galli, G., Bostedt, C., van Buuren, T. & Terminello, L. Quantum Confinement and Fullerenelike Surface Reconstructions in Nanodiamonds. *Phys. Rev. Lett.* **90**, 037401 (2003).

9. Duan, X. *et al.* Nanodiamonds in sp2/sp3 configuration for radical to nonradical oxidation: Core-shell layer dependence. *Appl. Catal. B Environ.* **222**, 176–181 (2018).

10. Barnard, A. S., Russo, S. P. & Snook, I. K. Size dependent phase stability of carbon nanoparticles: Nanodiamond versus fullerenes. *J. Chem. Phys.* **118**, 5094–5097 (2003).

Referee #2: Experiment

The manuscript presents a bottom-up synthesis method for producing molecular nanodiamonds (m-NDs) by applying a HPHT process to mNG (C96H30). The authors demonstrate the ND formation with control over particle size and morphology, and report the successful incorporation of color defect centers such as SiV and GeV by simply adding dopant precursors to the reaction. The process is supported by a combination of experimental techniques including SAXS, AFM, TEM, in situ ED-XRD, and complemented by ab initio calculations that provide insights into the growth mechanism and structure of the resulting NDs. The manuscript is well written. I believe the proposed synthesis method is interesting and has the potential to impact the development of high-quality ultrasmall NDs for quantum applications. However, I believe several aspects of the manuscript require further clarification and more rigorous experimental validation. The main claims related to size control, material purity, and surface chemistry are not sufficiently supported by the current data. Therefore, I cannot recommend the manuscript for publication in its current form.

We sincerely thank **Reviewer 2** for the thorough and constructive evaluation of our work. The reviewer's insightful comments have greatly strengthened both the experimental foundation and mechanistic interpretation of our study. Below we address each point in detail.

Q1: The authors claim that the bottom-up HPHT process allows for improved control over ND size compared to conventional top-down techniques such as milling or detonation. However, the experimental data does not convincingly demonstrate this improvement. Fig 1c and Ext. Data Fig. 1f,i show particle size distributions measured by different techniques such as AFM, SAXS, and TEM. However the values of mean and standard deviation differ across these methods, and the manuscript does not discuss the reasons for this variation. A quantitative comparison of size dispersion, for instance by calculating and comparing the standard deviation-to-mean (std/mean) ratio for each method and sample, can help support the claim. It would also be helpful to compare these parameters to those of conventional NDs.In addition, the use of Raman spectroscopy to correlate particle size with peak position and linewidth, particularly the diamond band, could strengthen the discussion. Previous studies [Mermoux et al. Diamond Rel. Mater. 87, 248–260, (2018)] have reported such dependencies, and including an alaysis like that reinforces the manuscript's arguments about size control and crystallinity.

We thank the reviewer for this important point. The differences in mean particle size and standard deviation obtained from TEM, AFM, and SAXS arise from the fundamentally different measurement principles and statistical weightings of these techniques. TEM provides a number-weighted, two-dimensional projected size of a limited number of selected m-NDs, while AFM measures height on a substrate. SAXS probes a large ensemble of particles and yields a volume-weighted average, where larger particles or minor aggregation contribute disproportionately. Therefore, identical size values are not expected, and the techniques should be considered complementary.

Additionally, in the revised manuscript we now (i) quantify dispersity using the standard-deviation-to-mean ratio (std/mean) for each characterisation method (TEM, AFM, SAXS) and morphology ,

and (ii) relate Raman diamond-band position/linewidth to crystalline quality following Mermoux et al.[1].

- Quantitative dispersity across methods and morphology TEM: m-NDs: 4.0 ± 1.4 nm with std/mean = 0.35 vs commercial milled and fractionated NDs: ND-NV-10 = 8.35 ± 4.24 nm (std/mean = 0.51) and ND-NV-40 = 27.87 ± 15.23 nm (std/mean = 0.55) (Wu et al.[2]), AFM: m-NDs: height: 3.4 ± 1.9 nm (N = 86; Extended Data Fig. 1g–i), std/mean = 0.56 vs commercial milled and fractionated NDs: ND-NV-40 (5.65 ± 1.47 nm; std/mean = 0.26 Lu & Vosberg et al.[3])
- Diameter/height: m-NDs diameter/height ≈ 1.2 (approximately equiaxed), vs ND-NV-40 diameter/height ≈ 4.9 (disk-like)
- SAXS: using FWHM ≈ 2σ, we obtain std/mean = 0.45 (m-NDs), and 0.28 commercial fractionated nanodiamonds. Comparing the m-NDs (approximately equiaxed) with commercial NDs (disk-like shape) is tricky since the fitting SAXS with different model introduces bias. Detailed data see table 1.

Although a direct quantitative comparison between fractionated commercial NDs and non-fractionated m-NDs is not possible, as fractionation artificially alters the size distribution, m-NDs exhibit a relatively narrow size distribution despite the absence of post-synthesis fractionation and display more uniform morphology. Detonation NDs were not included in this benchmark because they typically contain ~20 % sp^2 carbon (Duan et al.[4]).

<u>Raman correlation to size/crystallinity.</u>

We conducted Raman spectroscopy of m-ND using different laser powers. m-ND shows a peak position of 1329.3 cm^{-1} and FWHM of 31.3 cm^{-1} with a power of 0.1 mW. With the increasing power, the peak position shifts down to around 1318 cm^{-1}. The strong dependence of Raman shift on laser power corresponds to the characteristics of ultrasmall NDs (Mermoux et al.[5]). More specifically, we employ the model proposed by S. Osswald et al.[6] to calculate the coherence length of m-ND. A FWHM of 31.3 cm^{-1} corresponds to a coherence length of 4.9 nm. Note that coherence length is normally smaller than the actual particle size of NDs when there are defects (e.g. dislocations, vacancies, impurities) within the crystal lattice. For example, DNDs usually have a FWHM as high as 40 cm^{-1} (corresponding to a coherence length of 4.1 nm) with particle size of 4-8 nm as shown in Mermoux et al.[1]. In contrast, our m-NGs exhibits a coherence length of 4.9 nm comparable with particle sizes of 3-4 nm. Although the model does not provide a conclusive quantitative relationship between FWHM and coherence length, comparison with DNDs suggests that m-NDs contain significantly fewer defects. The high crystallinity of m-ND is also confirmed by the HRTEM images.

<u>Summary</u>

In the revised version, we now report the above std/mean values for TEM/AFM/SAXS, provide a methodology note explaining cross-technique differences, benchmark against commercial NDs, and include the Raman analysis that supports high crystallinity and nanoparticle uniformity. These results confirm that our nanographene-based HPHT fabrication method provides improved control over both size distribution and particle geometry. We note that small nanodiamonds produced by detonation typically contain ~20% sp^2 carbon (Duan et al.[4]) and were therefore not included in this comparison.

Changes to the manuscript: *Page 7, Lines 140-146 (Results): "To quantify size dispersity and morphology, we calculated the standard-deviation-to-mean (std/mean) ratio for TEM, AFM, and SAXS. m-NDs exhibit std/mean ratios of ~0.35, 0.56, and 0.45, respectively, and commercial fractionated nanodiamonds show std/mean ratios of ~0.55, 0.26, 0.28. Despite not undergoing post-synthesis fractionation, m-NDs shows a narrow size distribution. Geometry analysis further reveals that milled NDs are disk-like (diameter/height ≈ 4.9), while m-NDs are approximately equiaxed (diameter/height ≈ 1.2) based on TEM and AFM, further confirming more uniform morphology of m-NDs. The diamond Raman line at 1329.3 cm^{-1} with FWHM = 31.3 cm^{-1} corroborates high crystallinity and size uniformity[36]."*

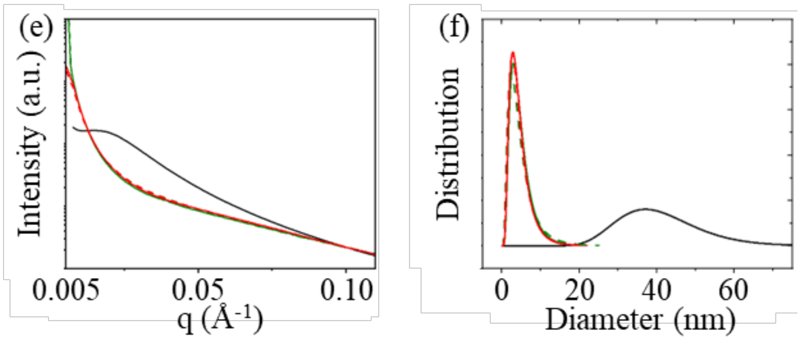

Extended Data Fig. 1: Comparison of HPHT treated hexabenzocoronene and m-NG. **e**, **f**, SAXS analyses of two different m-ND batches (ND1 solid line, ND2 dashed line) using different setups (setup A green, setup B red) compared to commercial 30 nm NDs (black, setup A).

Name	$\langle d \rangle$	D16	D50	D84	GSD	FWHM
CD-setupA	40.9	28.5	39.6	55.1	1.29	22.9
ND1-setupA	4.8	2.3	4.1	7.2	1.76	4.3
ND1-setupB	4.5	2.2	3.9	6.8	1.75	4.0
ND2-setupA	4.8	2.0	3.9	7.6	1.96	4.4
ND2-setupB	4.5	2.1	3.8	6.9	1.82	4.1

Table 1: Size-related values from SAXS analysis. $\langle d \rangle$ represents the mean particle diameter. D16, D50, and D84 are percentile diameters, meaning that 16%, 50%, and 84% of the particles are smaller than these values, respectively. The geometric standard deviation (GSD = √(D84/D16)) describes how broad the distribution is, while the full width at half maximum (FWHM) measures the width of the main peak and provides a length-scale estimate of the core population.

(c)

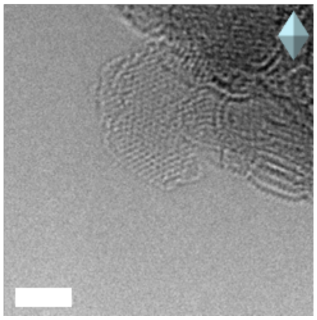

Fig. 2 c: HRTEM micrograph of m-ND, evidencing high crystallinity and a single sp² surface reconstruction. Scale bar, 2 nm.

(c)

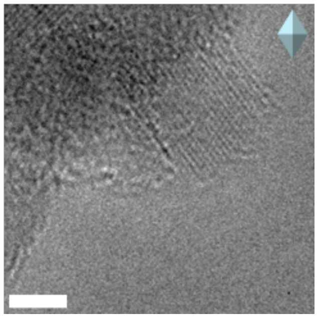

Extended Data Fig. 1 c: HRTEM micrograph of m-ND_{ox}, evidencing high crystallinity and a single sp² surface reconstruction. Scale bar, 2 nm.

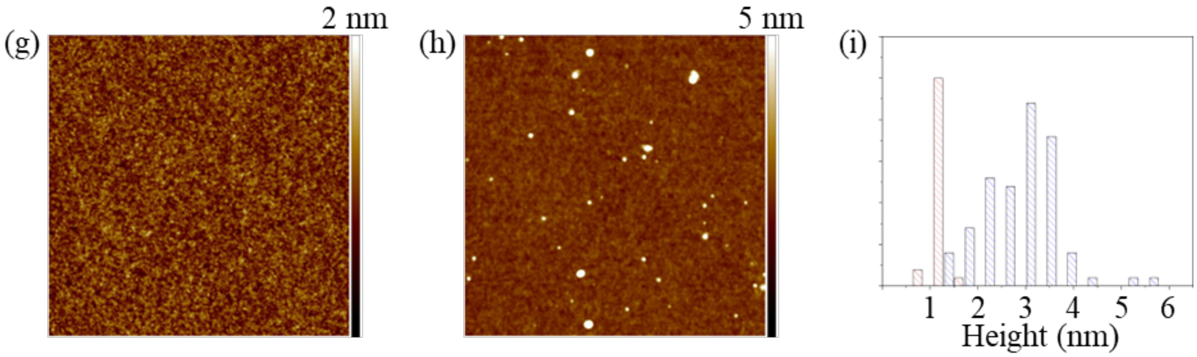

Extended Data Fig. 1: **Comparison of HPHT treated hexabenzocoronene and m-NG. g-i**, AFM images and analysis of a blank Si wafer **g** and of the same substrate after m-ND addition **h**. Lateral scan area: 1 × 1 µm². **i**, Distribution of diamond heights according to height analyses including images **g**, **h**. The mean size is 3.4 ± 1.9 nm, N = 86 particles.

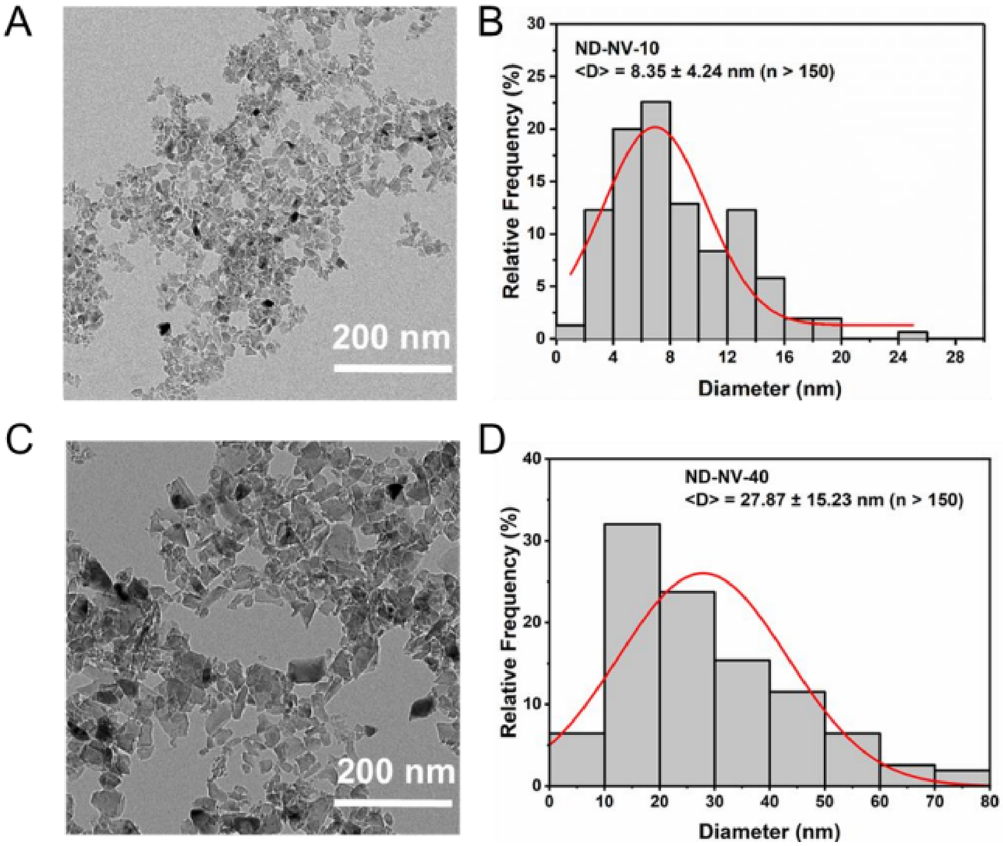

External Figure from Y. Wu, et al.[2]: B and D show the particle size distribution of commercial ND-NV-10 and ND-NV-40, respectively.

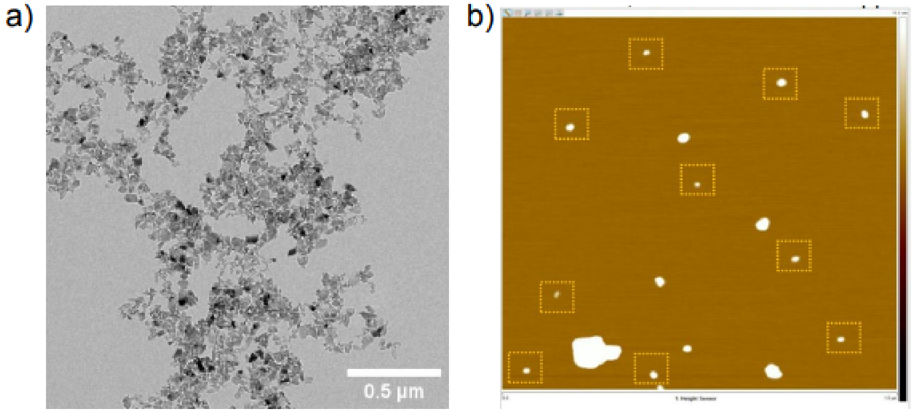

External Figure from Q. Liu and B. Vosberg et al.[3]: b) AFM analysis of commercial ND-NV-40 nanodiamonds.

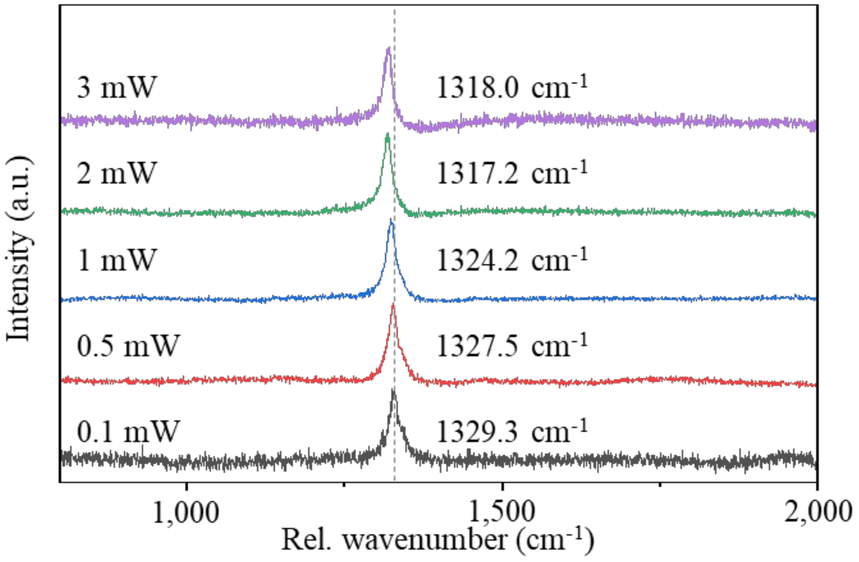

Supportive Figure: Raman analyses of m-ND excited at different laser power. The diamond Raman peak downshifts with increasing laser power. The excitation wavelength is 532 nm.

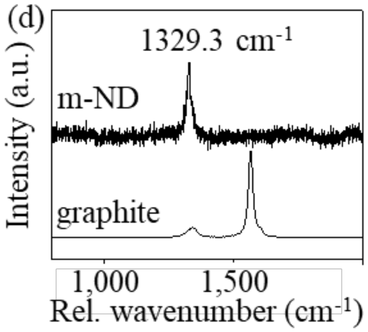

Extended Data Fig. 1: Comparison of HPHT treated hexabenzocoronene and m-NG. d, Raman spectra of untreated m-ND and graphite after HPHT (graphite).

Q2. Although size is discussed extensively, the morphology of the NDs is not discussed. This is a significant omission, as shape can strongly influence properties such as fluorescence brightness and optical homogeneity. Conventional top-down NDs often exhibit rod- or plate-like shapes (e.g., Eldemrdash et al., Carbon 206, 268 (2023); Oshimi et al., ACS Nano 18, 35202 (2024); Haotian et al., ACS Nano 17,16491 (2023)). If the present synthesis method truly enables morphological uniformity, this would be an important advantage, but the manuscript does not present data to demonstrate this. In addition, the TEM images provided have relatively low magnification (e.g., 20 nm scale bars), which is not sufficient for assessing particle shape or observing lattice patterns. Given that the manuscript refers to HR-TEM, higher-resolution images should be provided to show lattice structures extending to the surface of particles. Such data could also reveal the presence of sp^2-rich surface layers or graphitic contamination. Additionally, performing statistical

shape analysis on a representative number of particles is valuable to support the claim of morphological control.

We thank the reviewer for emphasising the importance of morphology and lattice-resolution imaging. In the revised manuscript we have substantially expanded our structural analysis, incorporating new Cs-double-corrected HRTEM and spatially resolved EELS measurements together with statistical evaluation of particle morphology. These data collectively demonstrate that the m-NDs produced via our bottom-up HPHT route are highly crystalline, morphologically uniform, and markedly more faceted and structurally pure than conventional top-down nanodiamonds.

<u>High-resolution HRTEM imaging of morphology and faceting (see HRTEM image in response in Q1).</u>

New HRTEM micrographs (Fig. 2c and Extended Data Fig. 1c) acquired using a Cs-double-corrected (S)TEM reveal well-defined lattice fringes extending to the particle surface for both crude and acid-treated m-NDs. The nanodiamonds exhibit an approximately equiaxed faceted morphology, consistent with known structural motifs of ultrasmall NDs. Lattice fringes corresponding to the (111) family of planes are evident across the entire particle, confirming high crystallinity. These images also show a single outer surface reconstruction with slightly increased spacing, producing the characteristic “wavy” edge profile typical of surface-relaxed nanodiamonds.

<u>Identification of an sp^2 surface reconstruction</u>

To assess surface hybridisation, we performed pixel-resolved EELS spectral imaging with 0.5 nm step size. The C K-edge spectra display the characteristic σ* resonances of diamond at 293, 298.5 and 306 eV, while a weak π* resonance at 285.5 eV increases at the particle periphery. PCA/k-means decomposition cleanly separates surface and bulk components, showing that the sp^2 contribution is confined to a single surface reconstruction (<0.5 nm). This behaviour is fully consistent for both m-ND and m-ND_{ox} samples. The presence of only one sp^2 reconstruction is noteworthy, as nanodiamonds of similar size generally exhibit thicker disordered or graphitic surface shells (e.g., Duan et al.[4]).

<u>Statistical morphology and size analysis.</u>

We now provide a quantitative morphology assessment from 49 independent particles (TEM) and 86 particles (AFM). m-NDs show a consistent equiaxed geometry, with diameter/height ≈ 1.2, contrasting sharply with commercial top-down NDs (e.g., ND-NV-40) which exhibit disk-like shapes (diameter/height ≈ 4.9). These statistics reinforce the morphological uniformity of our bottom-up nanodiamonds.

Changes to the manuscript: *Page 6, Line 128-135 (Results): “HRTEM images of both crude (Fig. 2c) and acid-treated (Extended Data Fig. 1 c) m-NDs reveal highly crystalline, faceted, approximately equiaxed nanoparticles. A single sp2 surface reconstruction is visible, appearing partially detached in some regions and giving rise to slightly wavy edges (Fig. 2c). EELS spectral imaging (Extended Data Fig. 4) confirms that this reconstruction is sp^2-rich, and the presence of such a thin reconstruction (<0.5 nm) is notable compared with the higher sp^2 fractions typically reported for nanodiamonds of similar size[34]. These combined results indicate that m-NDs possess minimal graphitic carbon and high structural purity.”*

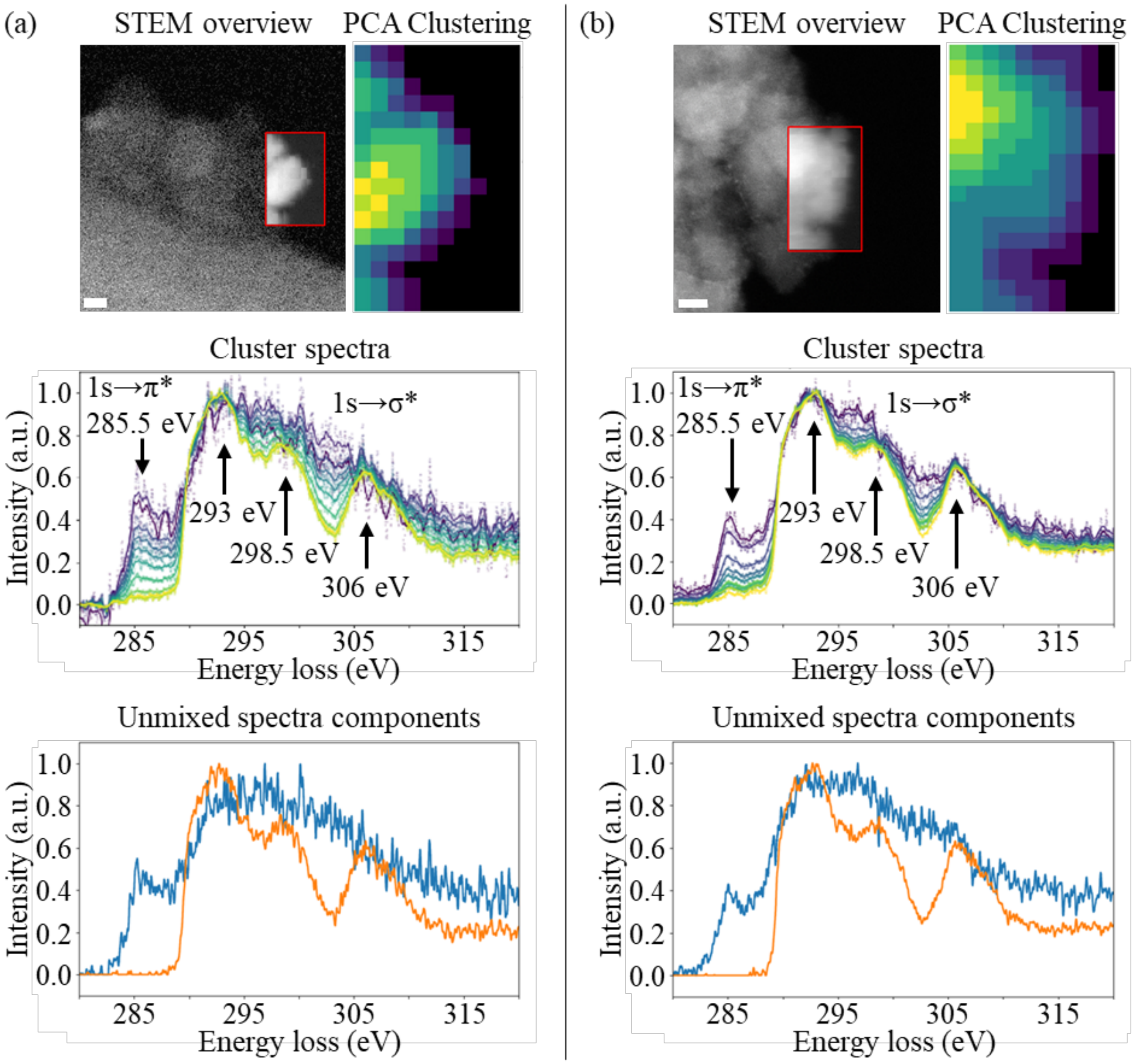

Extended Data Fig. 4: Site-specific EELS analysis of nanodiamond bonding and surface structure. a, EELS analysis of m-ND using a 0.5 nm steps Spectral Image (SI). The pixels in the SI were clustered in 10 regions (and an additional vacuum one) using PCA and k-means clustering on the EELS spectra. The average spectra of the clusters, shown normalised, were then used to reconstruct the EELS spectra of the surface (blue) and bulk (orange) components. The presence of the bulk component in the outermost region indicates that sp^2 surface reconstruction thickness does not exceed 0.5 nm. **b**, the same analysis was performed on m-NDox, showing qualitatively the same results. Scalebars are 2 nm.

Q3. The presence of GR1 centers shown in Fig 5c may indicate lattice defects or imperfections. A more discussion of GR1 centers, and ideally, further data should be included to assess the defect situations in these NDs more comprehensively. Further, EPR measurements is useful in this context, as they can quantify vacancy and impurity defects.

Since the manuscript emphasizes the scalability of the synthesis method, it is reasonable to expect that sufficient sample quantities can be produced for EPR analysis.

We thank the reviewer for this excellent suggestion. In response, we performed extensive X-band continuous-wave EPR spectroscopy on both SiV-fNDs and GeV-fNDs, enabling quantitative assessment of vacancy-related and impurity-related defects. These new data, together with a detailed analysis of GR1 centres, are now integrated into the revised manuscript.

GR1 centres (V^o)

We now clarify that the GR1 centre corresponds to the neutral monovacancy (V^o), which has a singlet ^1E ground state (S = 0) and is therefore EPR-inactive. Thus, the GR1 luminescence observed in Fig. 5c reflects isolated neutral vacancies, but does not imply extensive lattice damage. This is now explicitly stated in the manuscript.

EPR analysis of SiV-fNDs

The SiV-doped nanodiamonds exhibit a weak but characteristic signal near $g \approx 2$, which can be fit by two components:

- broad component: $g = 2.0031$, $\Delta B_{pp} = 0.80$ mT
- narrow component: $g = 2.0030$, $\Delta B_{pp} = 0.28$ mT

Such $g \approx 2$ multiplets are widely reported for ultrasmall nanodiamonds and arise from near-surface paramagnetic defects (e.g., dangling bonds, distorted carbon atoms) rather than bulk lattice damage (B. V. Yavkin et al.[7]).

No signals attributable to:

- SiV^- (S = 1/2; KUL8 defect),
- SiV^o (S = 1), or
- vacancy clusters (S = 1)

were detected. This is consistent with concentrations below ~500 ppb, where SiV-related EPR signals become buried in the strong $g \approx 2$ background (Nadolinny et al.[8]).

EPR analysis of GeV-fNDs

GeV-doped nanodiamonds also show two $g \approx 2$ components similar to those of SiV-fNDs. Importantly, the spectrum contains well-resolved 14N hyperfine triplet lines characteristic of substitutional nitrogen (P1 centres, S = 1/2), demonstrating the presence of trace nitrogen impurities. No signatures of the GeV^o centre (S = 1) were detected, again indicating concentrations below ~500 ppb (Nadolinny et al.[8]).

Together, the new EPR measurements confirm that

- no detectable paramagnetic vacancy clusters or V^-
- SiV and GeV concentrations are low but optically active;

- the dominant EPR signal originates from near-surface defects, typical for 3–30 nm nanodiamonds;

Thus, the defect landscape revealed by both PL (GR1) and EPR strongly supports the **high structural purity** of the bottom-up-synthesized nanodiamonds.

Changes to the manuscript: *Page 16, Lines 324-326 (Results): "A weak 741 nm GR1 feature, arising from neutral vacancies, indicates vacancy formation during HPHT synthesis even in the absence of irradiation."*

Results, Page 16, Lines 327-331 (Results): "EPR measurements on SiV-fND$_{ox}$ show a g ≈ 2 resonance (Extended Data Fig. 6a), attributable to near-surface defects and unpaired spins[47]. No resolved SiV- related EPR signatures are detected, likely due to spectral overlap and the absence of zero-field splitting or hyperfine structure. The KUL8 centre (S = 1/2), previously proposed to originate from SiV-[48], cannot be identified unambiguously; SiV^0 (S = 1) is not detected (<500 ppb)."

Page 16, Line 340-344 (Results): "GeV^0 (S = 1) is not detected (<500 ppb) by EPR; signals from g ≈ 2 centres and substitutional nitrogen (P1) are observed (Extended Data Fig. 6b). Charge-state identification is supported by the presence of donor species: EPR spectra of GeV-fNDs show P1 centres, while the absence of NV$^-$ emission reflects the deeper charge-transition levels of SiV$^-$ and GeV$^-$ in weakly compensated material."

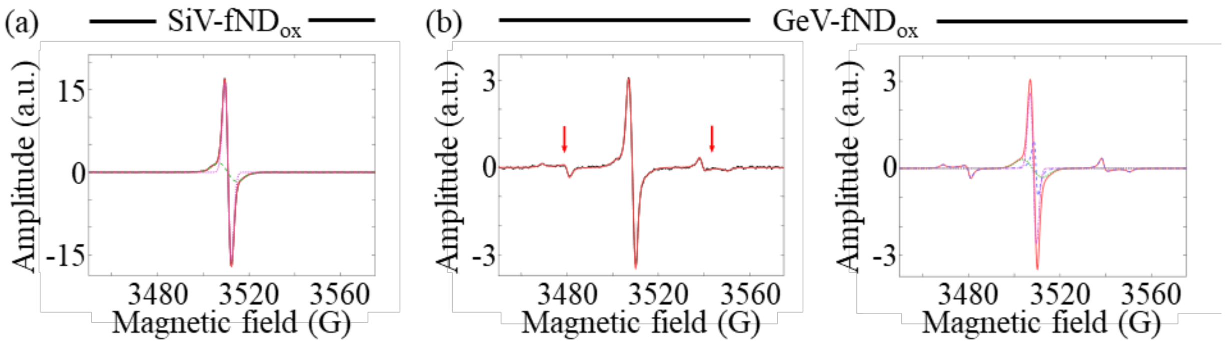

Extended Data Fig. 6 Spectral and optical properties of fluorescent nanodiamonds and control experiments. a and **b**, X-band continuous-wave EPR spectra and fits, for SiV- and GeV-doped sample. **a**, SiV-doped sample: experimental spectrum (black line) and fit (red line). The fit is performed with two components, labelled according to their relative linewidth as 'broad' (green, dashed) and 'narrow' (purple, dotted). The fit yields g=2.0031 and g=2.0030 for the broad and the narrow component, and corresponding peak-to-peak linewidths of 0.80 mT and 0.28 mT. **b**, Spectrum and fit for the GeV-doped sample. Left subfigure: Experimental spectrum (black line) and fit with three contributions (red line): g~2 components (broad and narrow), and substitutional nitrogen (P1 centers). Arrows indicate the groups of P1 hyperfine lines corresponding to the nuclear spin state of the 14N, mI = -1,+1. Right subfigure: Representation of the individual fit contributions. A microwave frequency of 9.84 GHz and a microwave power of 4.73 μW were used in the measurements.

Q4. For ultrasmall NDs, bulk-based techniques such as Raman and XRD are not sufficient to fully characterize the NDs. Electronic structure and bonding should be studied using more site-specific or surface-sensitive methods such as EELS or XAS. As many properties of ultrasmall NDs deviated from the bulk diamond crystals, the site-specific observations on the electronic structure will unambiguously address exact material situations of the present ND samples [Chang et al., ACS Nano 16, 8513 (2022). Ref. 23 in the present manuscript]. The authors need to perform EELS or XAS for their samples to fully confirm the acquisition of high quality NDs.

We thank the reviewer for underscoring the importance of site-specific electronic-structure characterisation for ultrasmall nanodiamonds. We fully agree that methods with nanometre-scale spatial resolution are essential, because ultrasmall NDs exhibit size-dependent deviations from bulk diamond properties, as highlighted by Chang et al.[9]. In response, we performed comprehensive electron energy-loss spectroscopy (EELS) with nanoscale spectral imaging, which directly probes the local bonding environment and electronic structure at the C K-edge.

Although carbon K-edge XAS would have been desirable, the beamline available at DESY does not provide the required sub–0.1 eV resolution for reliable deconvolution of π* and σ* features in ultrasmall NDs. EELS, by contrast, offers both the required energy resolution and the necessary real-space localisation, making it the appropriate tool for the present study.

<u>Site-specific EELS confirms high-quality diamond cores with a single sp^2 surface reconstruction (see response for Q2, Extended Data Fig. 4)</u>

EELS spectral imaging was performed with 0.5 nm spatial steps, allowing reconstruction of spatially resolved C K-edge signatures at both the surface and the diamond core of ~3–4 nm m-NDs and their oxidised derivatives. Across all particles analysed, the following characteristic features of crystalline diamond were observed:

- Diamond σ*: Three well-resolved σ* ionisation-loss peaks at: 293 eV, 298.5 eV, 306 eV corresponding to 1s → σ* transitions in sp^3-bonded carbon. The sharpness and relative intensities of these resonances match those of high-quality diamond and are inconsistent with amorphous or graphitic carbon.

Surface π*: A π* peak at 285.5 eV is present in all spectra, but its intensity increases exclusively at the particle periphery. This is characteristic of: a thin sp^2 surface reconstruction, and an sp^3-dominant diamond core.

This behaviour matches prior observations in ultrasmall diamonds (Chang et al.[9]; Raty et al.[10]).

<u>PCA/k-means spectral unmixing demonstrates a single sp^2 reconstruction</u>

To quantify the spatial distribution of bonding states, we conducted:

- principal component analysis (PCA)
- k-means clustering
- reconstruction of surface and bulk spectra
- semi-quantitative mapping of sp^2/sp^3 contributions

The reconstructed maps show:

- the bulk sp^3 component persists to within ~0.5 nm of the particle edge, and
- the sp^2 signal is strictly confined to a single surface reconstruction, with no evidence of multilayer graphitic shells.

This ultrathin sp^2 reconstruction is markedly less than the 2–5 nm amorphous carbon shells commonly reported for detonation or milled nanodiamonds of comparable size. Identical results were obtained for both m-ND and m-ND_{ox}, demonstrating that the sp^2 reconstruction is intrinsic to surface relaxation rather than oxidation artefacts.

These EELS results provide direct, site-resolved evidence that: the core of each nanoparticle is fully sp^3-bonded diamond, graphitic contamination is minimal, the surface consists of only one sp^2 surface reconstruction, consistent with surface reconstruction of (111) facets, and the overall bonding structure is consistent with high-quality ultrasmall diamond, not partially graphitic carbon. Thus, the bonding and electronic structure of the m-NDs are now comprehensively validated using surface-sensitive, atomically resolved spectroscopy, fully addressing the reviewer's concerns.

Changes to the manuscript: *Page 6, Lines 127–135 (Results): "…(HRTEM) consistently show 3–4 nm, monodisperse m-NDs (Fig. 2d, e; Extended Data Fig. 1e–i). HRTEM images of both crude (Fig. 2c) and acid-treated (Extended Data Fig. 1 c) m-NDs reveal highly crystalline, faceted, approximately equiaxed nanoparticles. A single sp2 surface reconstruction is visible, appearing partially detached in some regions and giving rise to slightly wavy edges (Fig. 2c). EELS spectral imaging (Extended Data Fig. 4) confirms that this reconstruction is sp^2-rich, and the presence of such a thin reconstruction (<0.5 nm) is notable compared with the higher sp^2 fractions typically reported for nanodiamonds of similar size[34]. These combined results indicate that m-NDs possess minimal graphitic carbon and high structural purity."*

Q5. The NDs containing SiV and GeV centers have significantly larger sizes (~30 nm) than the undoped m-NDs (~3 nm). This raises an important question about whether doping inherently leads to larger crystal growth, or whether it reflects an intentional or uncontrollable change in synthesis conditions. Since size and defect properties are central to the paper, a discussion on the cause of this difference is needed. Also, the above thorough study on the size dependence in combination with this defect-included NDs can strengthen the paper. Further, the applicability of the proposed [H]/[C] ratio model to doped NDs is not explained. Does this model still predict or control the size in the doped case? If not, why? The authors should clarify whether and how their theoretical framework can be extended to describe dopant-containing systems. If the size increase is unavoidable due to the requirements of incorporating SiV or GeV centers, this should be stated and justified clearly.

We thank the reviewer for raising this important mechanistic question. Understanding why SiV- and GeV-containing nanodiamonds (fNDs) grow to ~30 nm, significantly larger than undoped m-NDs, is indeed central to interpreting the generality of our bottom-up concept. In the revised manuscript, we now provide a fully expanded mechanistic discussion, supported by new TGA measurements, *in situ* ED-XRD experiments, and thermal stability analyses of both precursors and dopant molecules. These results clarify that the difference in size arises intrinsically from the two-component reaction kinetics and the thermodynamic role of dopants, rather than uncontrolled or inconsistent synthesis conditions.

Below we summarise the mechanistic basis for size amplification in doped samples and clarify the applicability of the [H]/[C] model.

Doped NDs originate from fundamentally different reaction pathways than undoped m-NDs

The one-component system (undoped m-NDs) affords predictable, hydrogen-limited growth because undoped nanodiamonds derive from a single, highly thermally stable nanographene precursor, m-NG ($C_{96}H_{30}$), which:

- remains structurally intact up to ~950 °C (10 % mass loss; TGA, Extended Data Fig. 7a),
- therefore, preserves a consistent [H]/[C] ratio throughout heating,
- ensuring reproducible nucleation and hydrogen-limited growth, yielding 3–4 nm NDs with narrow size dispersity.

Two-component doped system reveal early decomposition, amorphous carbon generation, and kinetic acceleration. In doped samples, the dopant precursors, tetrakis(trimethylsilyl)silane for SiV, melting at 319–321 °C, and tetraphenylgermane for GeV, melting at 230–235 °C, decompose before the HPHT reaction reaches the diamond nucleation regime.

This early decomposition produces:

- amorphous carbon lowering the [H]/[C] ratio largely
- gaseous fragments (H, CH_3, phenyl fragments), and
- dopant-containing radicals,
- which collectively dilute the effective hydrogen concentration and alter the carbon chemical potential.

Thus, doped samples necessarily evolve under non–hydrogen-limited conditions, shifting the growth dynamics toward larger nanodiamonds.

Dopants directly modify nucleation thermodynamics and promote particle coarsening

Silicon and germanium are known to lower the diamond–carbon interfacial energy. Computational and experimental analysis by Crane et al.[11] demonstrated that Si reduces the diamond–carbon interfacial energy sufficiently to:

- accelerate grain coalescence,
- increase ripening kinetics,
- stabilise larger crystallites, and

This provides a natural explanation for the almost tenfold increase in mean size: m-NDs: 3–4 nm, SiV-fNDs: 29 ± 8 nm, GeV-fNDs: 80–150 nm (similar behaviour observed). In other words, dopant incorporation is not merely additive, it rewires the thermodynamics of the carbon-to-diamond transition.

Thermal-stability assays confirm precursor-dependent hydrogen loss

TGA measurements (Extended Data Fig. 7a) show:

- m-NG ($C_{96}H_{30}$): 10 % mass loss at 950 °C
- HBC ($C_{42}H_{18}$): 10 % mass loss at 680 °C

Thus, the smaller nanographene HBC (used in our size-control study and in doped systems) undergoes decomposition ~275 °C earlier, releasing hydrogen prematurely. This earlier fragmentation lowers the [H]/[C] ratio, which in our model directly leads to larger nanodiamonds.

In situ ED-XRD confirms contrasting high-temperature pathways

In situ ED-XRD of coronene (an archetypal smaller graphene unit; Extended Data Fig. 7b) reveals:

- disappearance of its crystalline reflections above ~800 °C,
- graphite formation between 900–1000 °C, indicated by the (002) peak, and
- complete graphitisation upon extended heating at 1200 °C.

By contrast, m-NG does not graphitise under identical conditions; instead, it directly nucleates diamond below ~1200 °C. This essential distinction confirms that smaller, less stable precursors, and dopant precursors, reduce the [H]/[C] ratio earlier and thus yield larger diamonds.

Applicability and limits of the [H]/[C] model

Our revised manuscript now clarifies:

- The [H]/[C] model remains valid in doped systems, but only qualitatively.
- Reduced hydrogen availability leads to fewer surface terminations, which results in larger ND size
- In contrast, doped systems involve: multiple decomposition pathways, heterogeneous generation of amorphous carbon, dopant–carbon interactions, altered interfacial energies, and accelerated Ostwald ripening.

Thus, precise size prediction is not yet possible for two-component systems. However, importantly, even with these intrinsic complexities, the doped NDs produced here are significantly smaller and more monodisperse than previously reported SiV/GeV-doped nanodiamonds.

In summary, the significantly larger size of SiV- and GeV-fNDs is an intrinsic consequence of: precursor thermal stability differences, dopant-induced hydrogen depletion, altered nucleation thermodynamics, and dopant-driven coarsening kinetics. These effects arise inevitably in two-component doped systems and do not reflect uncontrolled synthesis. This mechanistic picture is now fully incorporated in the revised manuscript.

Changes to the manuscript:

Page 11, Lines 226-240 (Results):" The [H]/[C] model applies specifically to the intact $C_{96}H_{30}$ precursor under the HPHT conditions used here. m-NG is stable up to the onset of diamond formation, ensuring a well-defined hydrogen reservoir for surface passivation. Smaller or less stable precursors, such as coronene or HBC, decompose before diamond formation, altering hydrogen availability and generating amorphous carbon that promotes uncontrolled growth (Extended Data Fig. 7). Consequently, these precursors do not follow stoichiometric size predictions and yield larger, more heterogeneous nanodiamonds. Likewise, in two-component

dopant systems, decomposition of the dopant precursor and modified nucleation kinetics decouple the final nanodiamond size from the initial [H]/[C] ratio, clarifying the scope and limits of the H/C model."

Page 16-17, Lines 345-350 (Results): "Together, these results identify m-NG as a versatile molecular scaffold for bottom-up HPHT synthesis of fNDs containing SiV^- and GeV^- centers, with controlled defect incorporation, well-defined optical signatures, and narrower size distributions than in prior reports. The final fND size is decoupled from the [H]/[C] ratio, reflecting the decomposition of dopant precursors and modified nucleation kinetics in two-component systems, thereby clarifying the scope and limitations of precursor-encoded size control."

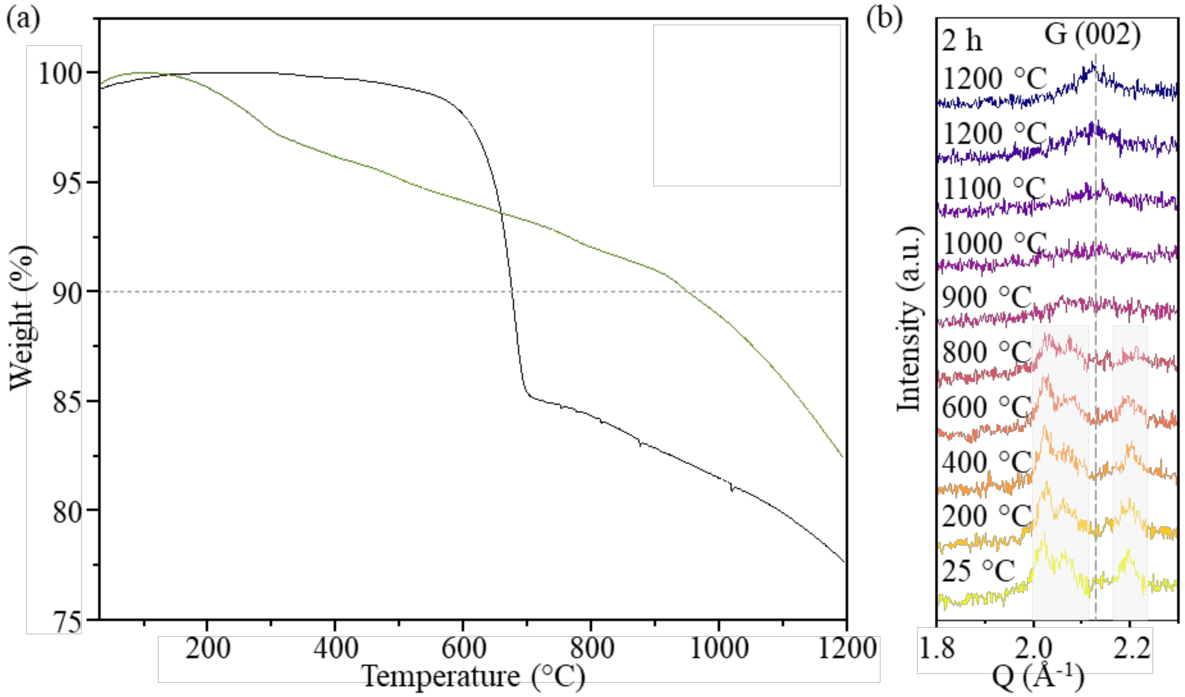

Extended Data Fig. 7: Stability analyses of ND precursors HBC, m-NG and coronene. a, TGA curve of HBC (black) and m-NG (green). A weight loss of 10% (dashed line) was measured at 680 °C (HBC) and 950 °C (m-NG), respectively. **b,** *In situ* ED-XRD of coronene at 13 GPa. Increasing the temperature from 25 °C up to 1200 °C results in the disappearance of the characteristic peaks of coronene (grey box) above 800 °C. Graphite formation starts between 800 and 900 °C depicted by the appearance of the (002) peak of graphite (grey dashed line). The peak becomes more evident after maintaining the temperature at 1200 °C for 2 h.

Q6. The strain in NDs can significantly affect the optical properties of color centers. For SiV centers, in particular, the width and distribution of ZPL can be used as an indicator of strain. The manuscript can be improved by analyzing the ZPL linewidths and comparing them with previous studies, such as Lindner et al. (New J. Phys. 20, 115002, 2018. Ref. 40 in the present manuscript), which relate optical line broadening to local strain fields

through ab initio modeling. Providing this type of analysis would not only strengthen the interpretation of defect quality but also highlight the quantum optical properties.

We thank the reviewer for this excellent suggestion. Strain-induced broadening of the SiV zero-phonon line (ZPL) is indeed a sensitive metric of local lattice distortions, and its quantitative evaluation provides a rigorous means to assess defect quality in nanodiamonds. In the revised manuscript, we now present an expanded analysis of the ZPL linewidths and centre wavelengths for 88 individual SiV-containing nanodiamonds, consistent with the strain–linewidth correlations established by Lindner et al.[12].

Lindner et al. demonstrated that variations in the local strain tensor lead to characteristic patterns in the ZPL parameter space. Their study predicts two distinct emitter classes, arising from differently oriented local strain fields, which manifest as distinguishable correlations between ZPL position and linewidth.

Our experimental dataset shows precisely this bimodal behaviour:

<u>Group V (Vertical branch):</u> strain-dominated linewidth broadening

- ZPL centred narrowly at 738 ± 2 nm
- but exhibiting FWHM values ranging from ~5 to ~25 nm
- consistent with inhomogeneous local strain within the host lattice.

This behaviour corresponds to the “vertical” group identified by Lindner et al., in which variations in the strain tensor perpendicular to the SiV symmetry axis broaden the ZPL without shifting its mean position.

<u>Group H (Horizontal branch): site variability with low strain</u>

- Very narrow ZPL linewidths (<5 nm)
- but a broad distribution of ZPL centre wavelengths (730–780 nm)

This matches the “horizontal” branch in Lindner et al., where emitters reside in distinct but low-strain local configurations, producing large shifts in centre frequency but only minimal linewidth broadening.

The excellent agreement with the strain–linewidth model of Lindner et al. confirms that the SiV centres in our bottom-up fNDs:

- occupy well-defined local environments,
- experience strain magnitudes consistent with high-quality nanoscale diamond, and
- exhibit optical homogeneity comparable to SiV centres in state-of-the-art nanodiamonds produced via conventional HPHT or CVD methods.

Furthermore, achieving such optical behaviour in ~30 nm SiV-fNDs synthesised directly from molecular precursors is significant, as it combines high optical quality with exceptionally small particle size, an advantage for quantum sensing and biological imaging where minimal dimensions and narrow linewidths are essential.

Changes to the manuscript: *"Page 16, Lines 331-336 (Results): "Analysis of 88 individual ZPLs at room temperature reveals two emitter groups (horizontal and vertical) (Extended Data Fig. 6c), matching the results of Lindner et al.[49] and indicating distinct but well-defined local environments. Linewidths lie within ranges reported for high-quality CVD-grown SiV-fNDs. notably, no nitrogen-vacancy centres are detected, even without fluorine-containing suppressors used previously[29,30]."*

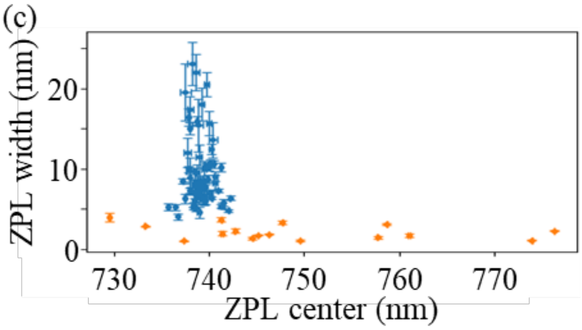

Extended Data Fig. 6: Spectral and optical properties of fluorescent nanodiamonds and control experiments. c, Measurement of ZPL centre position and ZPL width of 88 particles at room temperature. The data reveals two different groups of emitters. While Group V (blue) has a fixed central ZPL at around 738 nm but a large spread in ZPL width between about 5 and 25 nm, Group H (orange) has a constantly low ZPL width below 5 nm but a large spread of ZPL positions between 730 nm and 780 nm.

Q7: The authors describe a hydrogenated ND surface in their theoretical model, but it is unclear whether the synthesized particles are actually hydrogen-terminated after HPHT processing. The manuscript does not describe the collection process or mention whether any surface cleaning or termination steps were applied after the synthesis. Since the surface strongly influences both optical properties and chemical stability, details on this point are essential. IR spectroscopy and XPS are necessary to characterize surface terminations and functional groups. These measurements would also help clarify how well the actual material matches the model assumptions.

We thank the reviewer for highlighting this essential point. The revised manuscript now explicitly describes the post-synthesis collection procedure, clarifies the surface chemistry of as-prepared and acid-treated nanodiamonds, and provides a comprehensive experimental verification of hydrogen termination using multiple independent techniques (FTIR, XPS, zeta potential). These data confirm that our HPHT-grown nanodiamonds indeed possess hydrogen-terminated surfaces immediately after synthesis, which validates the assumptions used in our theoretical model.

<u>Collection and workup procedure (now described in Methods)</u>

After the HPHT reaction:

- The material is recovered directly from the reaction capsule.
- Crude samples retain hydrogen termination, consistent with the expected HPHT environment at high hydrogen chemical potential.

- When oxidative cleaning is required, we apply a standard tri-acid treatment (H_2SO_4/HNO_3/$HClO_4$, 1:1:1 v/v, 16 h reflux), which removes residual carbonaceous species and converts the surface to oxygen termination.

These steps are now clearly described in the revised Methods section.

Surface termination analysis of as-prepared (crude) nanodiamonds

FTIR spectroscopy (Extended Data Fig. 3a)

- Crude m-NDs, SiV-fNDs, and GeV-fNDs all show C–H stretching bands at ~2830 and 2920 cm^{-1}.
- A characteristic dip at ~1331 cm^{-1} (Fano-type interference) is detected, a hallmark of hydrogenated nanodiamond surfaces (Saoudi et al.[13]; Kudryavtsev et al.[14]).
- After acid cleaning, strong C=O stretching features appeared, confirming conversion to oxygen termination.

XPS (Extended Data Fig. 3b–e)

- Crude nanodiamonds show dominant C–C/C–H contributions at 284.8 eV.
- Acid-treated m-ND_{ox} exhibit a substantial C=O component in the C 1s region and an O 1s shift from 532.3 → 531.7 eV, characteristic of carbonyl-rich oxidised surfaces.

Zeta potential (Extended Data Fig. 3f)

- Crude m-NDs: +8.2 ± 0.6 mV
- Crude SiV-fNDs: +10.7 ± 0.7 mV
- Crude GeV-fNDs: +21.3 ± 1.0 mV
- Acid-treated samples shift to negative zeta potentials (−15 to −21 mV), consistent with carboxylated and hydroxylated surfaces.

Together, these complementary measurements confirm that as-prepared nanodiamonds are hydrogen-terminated, validating the assumptions in our theoretical hydrogenation model.

Alignment with theory

The hydrogenated surface of the as-synthesized NDs provides the correct boundary condition for our theoretical model, which assumes:

- Monohydride termination of surface carbon atoms,
- Facet-dependent hydrogen coverage, and
- A corresponding [H]/[C] ratio that governs nanodiamond size selection.

Because the experimental data unambiguously show hydrogen termination on the crude NDs, the model assumptions faithfully represent the actual material state.

Changes to the manuscript: *Page 6, Lines 121-125 (Results): "FTIR spectra (Extended Data Fig. 3a) reveal distinct C–H stretching modes centred around 2830 cm^{-1} and, together with the*

positive zeta potential of +8.2 ± 0.6 mV (Extended Data Fig. 3f), confirm hydrogen termination of m-NDs[35]. Acid treatment produces oxidised m-NDs (m-ND$_{ox}$), with characteristic C=O features in XPS (Extended Data Fig. 3b–e), FTIR signatures and a negative zeta potential of −15.4 ± 0.5 mV (Extended Data Fig. 3f)."

Page 21, Lines 444-447 (Methods): "The nanodiamond samples were oxidized using a mixture of H_2SO_4, HNO_3 and $HClO_4$ (volumetric ratio 1:1:1) and heated to 90 °C under sonication. Afterwards, the samples were washed with MilliQ-H_2O and centrifuged for 20 min at 4500 rpm. These steps were repeated for three times."

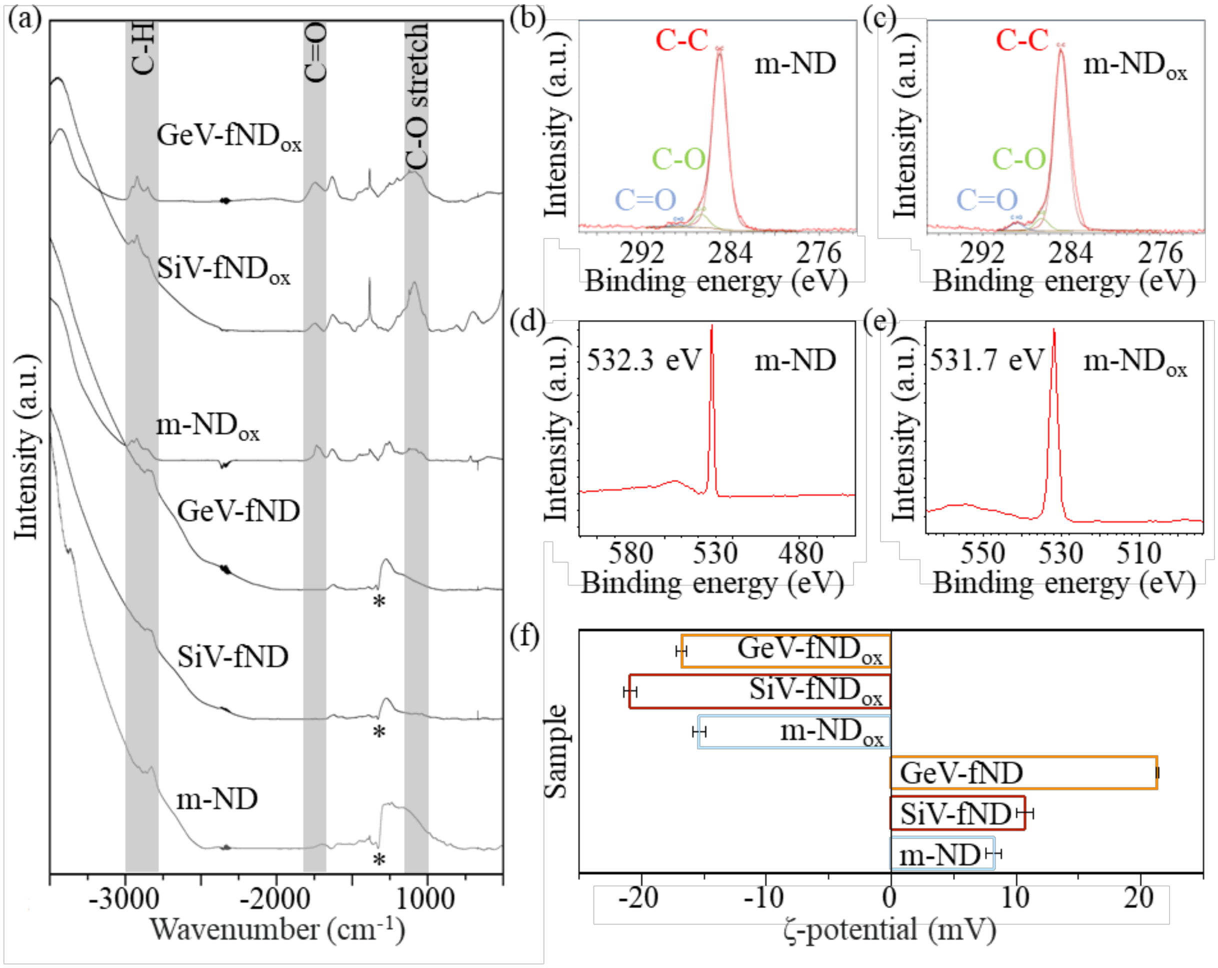

Extended Data Fig. 3: Surface characterisation of nanodiamonds. **a**, FTIR spectra of untreated nanodiamonds (m-ND, SiV-fND and GeV-fND) and treated nanodiamonds (m-ND$_{ox}$, SiV-fND$_{ox}$ and GeV-fND$_{ox}$). The dip at ~1331 cm-1 (*) is associated with Fano-type interference in hydrogenated nanodiamonds. **b**, XPS spectrum of untreated m-ND focusing on the C-region. **c**, XPS spectrum of m-ND$_{ox}$ focusing on the C-region showing increased C=O species. **d**, XPS spectra of untreated m-ND and **e** m-ND$_{ox}$ focusing on the O-region. **f**, Zeta potential of the untreated (m-ND, SiV-fND, and GeV-fND) and the acid treated samples (m-ND$_{ox}$, SiV-fND$_{ox}$ and GeV-fND$_{ox}$). The positive zeta potentials are indicative of hydrogenated ND surfaces, while the negative zeta potentials reflect surface oxidation.

Minor Comments

Q8: In the abstract, the term "superphenalene" is used, while the title refers to "nanographene." It is better to clarify this terminology, since coronene, HBC, and m-NG are actually used in the experiments.

• In Ext. Data Fig. and captions, notations such as "(a+b)" are confusing and should be revised to specify each panel explicitly.

• The use of labels like "Gottingen" and "MPIP" should be avoided or replaced with more meaningful ids with captions. And I can't understand what these four NDs mean.

Additional note. I couldn't find a video for extended data, unfortunately.

We thank the reviewer for these careful and constructive presentation-related observations. We have implemented all suggested revisions to improve clarity, consistency, and readability across the entire manuscript and Extended Data.

<u>Terminology harmonisation ("superphenalene" vs "nanographene")</u>

We agree that the terminology should be consistent and unambiguous. The term superphenalene, historically used to describe the extended polycyclic aromatic system $C_{96}H_{30}$, has been replaced throughout the abstract and main text with the more precise term molecular nanographene (m-NG). We use the term nanographene as a broad designation for the family of polycyclic aromatic hydrocarbons, including coronene, HBC, and m-NG.

<u>Panel notation in Extended Data figures</u>

Ambiguous references such as "(a+b)" have been removed. All figure captions have been rewritten to:

- Refer explicitly to each panel (e.g., "Extended Data Fig. 1e" rather than "(a+b)"),
- Describe each panel's contents clearly and independently,
- Maintain consistency with Nature figure-caption conventions.

<u>Replacement of non-descriptive labels ("Göttingen," "MPIP")</u>

We appreciate the reviewer pointing out these ambiguous identifiers. All instrument- and facility-specific labels have been replaced with Setup A and Setup B, which refer to the two small-angle X-ray scattering (SAXS) configurations used in the study.

Additionally, the nanodiamond labels have been clarified:

- ND1 and ND2 now designate two independent m-ND batches produced under identical conditions.
- CD denotes the 30-nm commercial nanodiamond reference sample.

These identifiers are used consistently across the text and Extended Data.

<u>Correction of figure cross-references</u>

The incorrect reference to "Fig. 4d" has been corrected. All cross-references have been systematically verified.

Availability of the Extended Data video

We confirm that the Extended Data videos showing the behaviour of m-NG under increasing pressure are included in the final submission package and are referenced appropriately in the manuscript.

References:

1. Mermoux, M., Chang, S., Girard, H. A. & Arnault, J. C. Raman spectroscopy study of detonation nanodiamond. Diam. Relat. Mater. 87, 248–260 (2018).
2. Wu, Y. et al. Detection of Few Hydrogen Peroxide Molecules Using Self-Reporting Fluorescent Nanodiamond Quantum Sensors. J. Am. Chem. Soc. 144, 12642–12651 (2022).
3. Lu, Q. et al. Unraveling Eumelanin Radical Formation by Nanodiamond Optical Relaxometry in a Living Cell. J. Am. Chem. Soc. 146, 7222–7232 (2024).
4. Duan, X. et al. Nanodiamonds in sp2/sp3 configuration for radical to nonradical oxidation: Core-shell layer dependence. Appl. Catal. B Environ. 222, 176–181 (2018).
5. Mermoux, M., Crisci, A., Petit, T., Girard, H. A. & Arnault, J.-C. Surface Modifications of Detonation Nanodiamonds Probed by Multiwavelength Raman Spectroscopy. J. Phys. Chem. C 118, 23415–23425 (2014).
6. Osswald, S., Mochalin, V. N., Havel, M., Yushin, G. & Gogotsi, Y. Phonon confinement effects in the Raman spectrum of nanodiamond. Phys. Rev. B 80, 075419 (2009).
7. Yavkin, B. V., Mamin, G. V., Gafurov, M. R. & Orlinskii, S. B. Size-dependent concentration of N0 paramagnetic centres in HPHT nanodiamonds. Magn. Reson. Solids 17, 1–7 (2015).
8. Nadolinny, V. et al. Silicon‐containing defects in HPHT diamond synthetized in Mg–Si–C system. Phys. status solidi 212, 2460–2462 (2015).
9. Chang, S. L. Y., Reineck, P., Krueger, A. & Mochalin, V. N. Ultrasmall Nanodiamonds: Perspectives and Questions. ACS Nano 16, 8513–8524 (2022).
10. Raty, J.-Y., Galli, G., Bostedt, C., van Buuren, T. & Terminello, L. Quantum Confinement and Fullerenelike Surface Reconstructions in Nanodiamonds. Phys. Rev. Lett. 90, 037401 (2003).
11. Crane, M. J. et al. High-pressure, high-temperature molecular doping of nanodiamond. Sci. Adv. 5, (2019).
12. Lindner, S. et al. Strongly inhomogeneous distribution of spectral properties of silicon-vacancy color centers in nanodiamonds. New J. Phys. 20, 115002 (2018).
13. Saoudi, L. et al. Colloidal stability over months of highly crystalline high-pressure high-temperature hydrogenated nanodiamonds in water. Carbon N. Y. 202, 438–449 (2023).
14. Kudryavtsev, O. S. et al. Fano-type Effect in Hydrogen-Terminated Pure Nanodiamond. Nano Lett. 22, 2589–2594 (2022).

Referee #3: Theory

The study "Bottom-up Synthesis of Molecular and Fluorescent Nanodiamonds from Nanographene" presents a novel synthesis method for hard-to-obtain nearly-monodisperse nanodiamonds, potential hosts for color centers. It is supported by impressive experimental data. The precursor is 'molecular nano-graphene', m-NG: C96H30 and C42H18. At conditions (1200 C, 13 GPa) close to those in making diamond phases, a complete conversion of m-NG into nanodiamonds m-ND is achieved, with very narrow size distribution, and negligible amount of sp^2-carbon (absence of G bands). Presented results are quite remarkable and deserve publication. However, the mechanistic explanation, theoretical models presently employed, are far inferior to the experimental findings claimed, significantly diminishing the work's rigor, insufficient for publication in Nature in present form. The specific critique to be addressed in a major revision is below.

We sincerely thank Reviewer 3 for the insightful and constructive comments, which have helped us to further strengthen our manuscript. In the revised manuscript, we have now substantially expanded and refined the theoretical analysis of the transition of nanographene into diamond. These new computational studies complement our experimental findings. Specifically, the simulations have been extended into a state-of-the-art computational study of the molecular-to-diamond transition. We now provide a detailed analysis of the transformation pathway, including coordination number, bond-angle distribution, and orientational order parameter evolution, that supports the emergence of diamond-like structural motifs, consistent with the experimental observations from ED-XRD, Raman, HRTEM, and EELS. Furthermore, we now analyse the electronic density of states (DOS) along the reaction trajectory, demonstrating a clear disappearance of π-states and the formation of a widening σ-band gap.

Q1: The phase diagram in Fig. 1, borrowed from bulk graphite studies, is not fully appropriate for hydrogenated molecular precursors. A discussion appearing perhaps more relevant to this work, with chemically induced phase transitions, e.g. [10.1021/jz5007912] see Fig 7, also [10.1002/smll.202004782] Figs. 2-3, is needed to contextualize the mechanism. Even if all available H originates only from those C_xH_y edges, it may be enough to promote the 'basal plane' chemical activation sp2-->sp3, and also explain why pressure needed in experiments appears tenfold lower than suggested here models predict.

We thank the reviewer for this relevant comment and we fully agree that the bulk graphite–diamond phase diagram, although the standard reference within the diamond-synthesis community, does not quantitatively capture chemically assisted rehybridisation pathways in hydrogenated nanographene precursors such as m-NG ($C_{96}H_{30}$) and HBC ($C_{42}H_{18}$). In the revised manuscript, we now explicitly distinguish between (i) the thermodynamic boundary for bulk carbon and (ii) the chemically activated molecular-to-diamond transformation relevant to our system. To address this properly, we have substantially expanded the mechanistic discussion and incorporated the literature cited by the reviewer, particularly the chemically induced phase transition pathways identified in Sun et al., J. Phys. Chem. Lett. 2014 (Fig. 7)[1] and the hydrogen-assisted diamond-like reconstruction pathways reported in Erohin et al., Small 2021 (Figs. 2–3)[2]. These studies demonstrate that hydrogen can drastically reduce the activation barrier for $sp^2 \rightarrow sp^3$ conversion by stabilising partially rehybridised intermediates and promoting bond rearrangement along the basal planes. The key clarifications that have been added to the manuscript are listed below:

Hydrogen at nanographene edges provide a chemically activated transformation pathway

- m-NG precursors contain 30% ($C_{96}H_{30}$) hydrogen-terminated edge sites.
- Our *ab initio* MD simulations at 0 K (Fig. 4a, Extended Dat Fig. 5c, d, Extended Data Video ab_initio_0K) show that these hydrogens induce: local out-of-plane distortions, weakening of π-conjugation at the edges, initial formation of four-coordinated carbon motifs before full nucleation. These distortions create a chemical pressure that lowers the effective transition barrier relative to bulk graphite.

Hydrogen stabilises sp^3-like intermediates under HPHT conditions

Newly added analysis shows:

- Concomitant sharpening in the bond-angle distribution toward the tetrahedral range.
- Reduction of π-density in the projected DOS and emergence of a σ-dominated gap along the transition pathway.

These observations are consistent with hydrogen-assisted mechanisms proposed in the papers cited by the reviewer.

Why pressure needed in experiments appears tenfold lower than suggested here models predict

Our expanded theoretical framework explains the experimentally observed transformation of m-NG at 13 GPa, despite bulk graphite requiring 80–100 GPa for purely thermodynamic diamond stability:

- The reaction also proceeds through chemically activated, not purely pressure-driven, pathways.
- Hydrogen lowers the barrier for basal-plane bond rotation and $sp^2 \rightarrow sp^3$ rearrangement.
- Finite molecular precursors bypass the need for long-range order required in graphite.
- *Ab initio* simulations incorporate the actual C:H stoichiometry, ensuring that chemically assisted pathways are automatically included within the computational model.

Why we retain the classical phase diagram

To maintain continuity with the diamond-synthesis literature, the bulk phase diagram remains in the paper, but it is now presented explicitly as a thermodynamic reference, not a mechanistic predictor. The revised text emphasizes that the molecular precursors do not follow the graphite path, and their transformation is governed instead by chemically assisted local rehybridisation.

Changes to the manuscript: *Page 9, Lines 186-192 (Results):" The classical graphite–diamond phase diagram does not account for hydrogen assisted $sp^2 \rightarrow sp^3$ transitions relevant to hydrogen terminated nanographenes. Prior work shows that hydrogen at molecular edges stabilises sp^3 intermediates and lowers the activation barrier for diamond nucleation under HPHT conditions[39,40]. The high density of edges, curvature and domain boundaries in m-NG provides numerous energetically favourable sites for interlayer bonding and tetrahedral rehybridization. These pathways could rationalize why molecular nanographenes transform rapidly at pressures below those required for bulk graphite."*

Page 12, Lines 247-257 (Results): "We analysed the simulations by tracking coordination numbers, bond-angle distributions and tetrahedral order parameters. Across the 20–100 GPa window, the m-NG supercell evolves from a graphite-like to a diamond-like configuration. The average C–C coordination number (cutoff 1.8 Å) increases from ~2.7 at 20 GPa toward ~3.4 at 100 GPa, while the mean C–C–C bond angle shifts from ~120° (sp²-like) to ~111° (close to the 109.5° of sp³ carbon) (Fig. 4c, solid lines). The sp²→sp³ transition occurs at ~78 GPa, which is also confirmed by the orientational tetrahedral order parameter (Extended Data Fig. 5g) that indicates the formation of a diamond-like structure at this pressure. These trends mirror experimental XRD, Raman and HRTEM observations. The electronic density of states (Fig. 4d) shows a gradual disappearance of π-states and the emergence of a σ-bandgap of ~4 eV. Simulated lattice spacings match experimental values, reinforcing the mechanism."

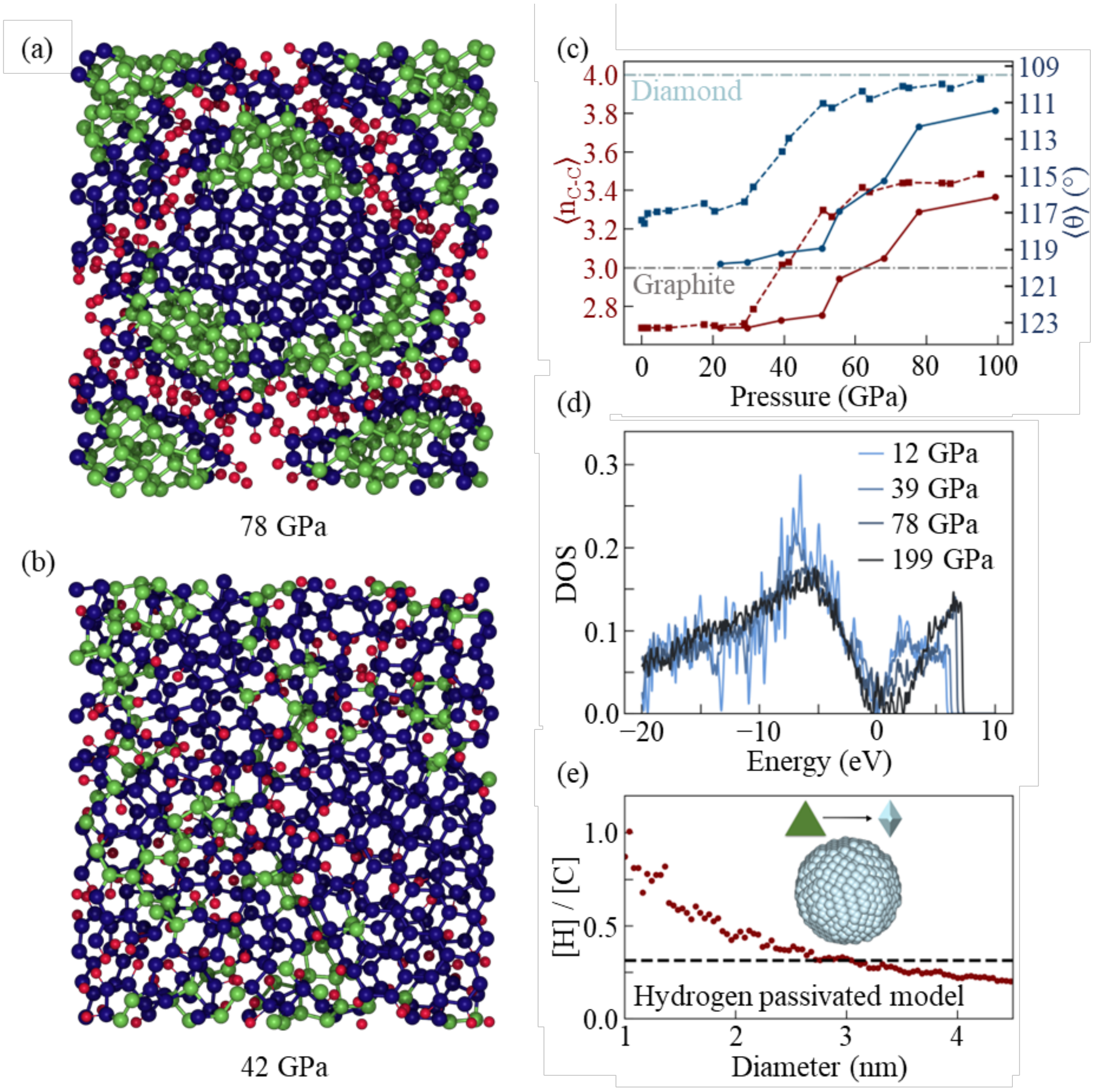

Fig. 4 | Pressure-induced transformation of nanographene into diamond-like structures.

a, *Ab initio* simulations at 0 K showing the structural evolution of the m-NG supercell as the simulation volume is reduced, corresponding to a pressure of 78 GPa. Increasing pressure induces layer subduction, molecular bending and out-of-plane distortions, signaling the onset of $sp^2 \rightarrow sp^3$ rehybridization. Blue denotes sp^2-hybridized carbon, green denotes sp^3-hybridized carbon, and red denotes hydrogen. **b**, MLIP simulations of the m-NG supercell at 1,500 K and 42 GPa, showing that diamond nucleation occurs at lower pressures with increasing temperature. Blue denotes sp^2-hybridized carbon, green denotes sp^3-hybridized carbon, and red denotes hydrogen. **c,** Average carbon–carbon coordination number $\langle n_{C\text{-}C} \rangle$ (red) and average C-C-C bond angle $\langle \theta \rangle$ (blue) of the m-NG supercell at 0 K (solid line, *ab initio*) and 1500 K (dashed line, MLIP) as a function of pressure from 0 to 100 GPa. Dashed horizontal lines mark the limiting values for ideal graphite ($\langle n \rangle = 3$, $\theta = 120°$) and diamond ($\langle n \rangle = 4$, $\theta = 109.5°$). The monotonic evolution toward diamond-like coordination reflects the progressive formation of tetrahedral carbon which occurs at lower pressures as temperature increases. **d**, Electronic density of states (DOS) for different values of pressure in our *ab initio* simulations. A gap of approximately 4 eV fully develops at 199 GPa, consistent with the gap reported for diamond within the GGA approximation (5.45 eV). **e,** Calculated ratio r = [H]/[C] required to passivate spherical hydrogen-terminated nanodiamond clusters of diameter d. The horizontal line marks r = 30/96 = 0.3125 for m-NG, intersecting the model at d ≈ 3 nm, consistent with the experimentally observed m-ND diameter.

Indeed, hard to be convinced that the shown atomistic simulations adequately represent the formation process, being entirely focused on increased pressure 155 GPa ramped up 10 times above the experimental 12-13 GPa; in fact the lowest simulated value 13.8 GPa is near the topmost in experiments.

We thank the reviewer for this important observation. We agree that direct quantitative comparison between experimental and simulated transformation pressures is not feasible due to the well-known limitations of *ab initio* molecular dynamics: finite system size (hundreds of atoms), picosecond time scales, and the need to accelerate rare nucleation events that would otherwise occur over macroscopic timescales. As in most studies of first-order phase transitions, the transformation is therefore induced by driving the system beyond the metastable regime, effectively crossing the spinodal boundary, which necessarily requires higher pressures and lower activation barriers than those relevant experimentally.

In the revised manuscript, we now clarify this point and, more importantly, substantially extend the theoretical analysis to demonstrate that, despite the elevated pressures required for computational tractability, the simulations capture a mechanistically faithful and experimentally validated molecular-to-diamond transformation pathway. Furthermore, we have extended the computational scope and employed a machine-learning interatomic potential (MLIP) to simulate the transformation across a range of temperatures using molecular dynamics which is discussed below.

<u>Structural evolution</u>

We now provide a detailed structural analysis along the full reaction trajectory:

- Coordination number increase from predominantly 3-coordinated carbon to 3.4–3.5 (Approaching sp^3, Fig. 4c).
- C–C–C bond-angle sharpening toward the tetrahedral region (≈109.5°, Fig. 4c).

- Orientational tetrahedral order parameter q (Extended Data Fig. 5g) showing a continuous shift from a broad, disordered distribution to a peak near $q \approx 0.8$, characteristic of diamond-like local environments.

These signatures mirror those resolved experimentally by *in situ* ED-XRD, Raman, and HRTEM, demonstrating that the simulated transformation pathway reproduces the correct local structural physics, even if absolute pressures differ.

<u>Electronic-structure evolution confirming the correct bonding transformation</u>

We have now included projected DOS calculations (Fig. 4d), showing that:

- π-states disappear progressively along the pathway;
- σ-band gap widens, reaching ≈4 eV at 199 GPa, consistent with the GGA-calculated bandgap of diamond (≈5.45 eV, Correa et al.[3]).

This electronic reorganisation is fully consistent with the emergence of tetrahedral bonding and is directly correlated with the absence of Raman G-bands and sharpening of the 1329.8 cm^{-1} diamond mode in experiment.

<u>Simulated lattice spacings match experiment</u>

The (D111) spacing of the simulated diamond domains (≈2.06 Å) closely matches:

- HRTEM lattice fringes of m-ND (Fig. 2c, Extended Data Fig. 1c),
- SAED diffraction pattern (Fig. 2b)
- XRD peak (Fig. 2a).

This agreement supports the structural realism of the simulated product cluster.

<u>The goal of the simulations is mechanistic, not quantitative</u>

We now explicitly state that:

- The *ab initio* simulations are not intended to reproduce the precise thermodynamic conditions of diamond formation but to reveal the atomistic sequence of structural and electronic reorganisation during the $sp^2 \rightarrow sp^3$ transition under the correct C:H stoichiometry.

- We used a machine learning interatomic potential (MLIP) to simulate the transformation at a range of temperatures using molecular dynamics. Our MLIP is the state-of-the-art MACE foundation model (MACE-MH-1), fine-tuned using our own *ab initio* data. At 200 K under compression, the $sp^2 \rightarrow sp^3$ transition is clearly visible at 60 GPa, and at 1500K at 42 GPa which is closer to the experimental conditions. Decompression simulations are used to verify that newly formed neighbouring nanodiamonds remain essentially separate. While the time scale of MLIP simulations are many orders of magnitude larger than those achievable using explicit electronic structure methods, direct molecular dynamics still falls short of experimental time scales. The simulations show evidence of nucleation, and as these are rare events, it is expected that transition pressures in the relatively fast

compression simulations (100 ps) would be higher than in reality. The mechanism of nanodiamond formation is clearly visible in our simulation, and the role of capping hydrogens is limiting the growth of the nanodiamonds.

Taken together, the expanded structural, electronic, and geometric analyses and the MLIP results significantly strengthen the mechanistic connection between simulation and experiment.

Changes to the manuscript*: Page 12, Lines 247-257(Results) "We analysed the simulations by tracking coordination numbers, bond-angle distributions and tetrahedral order parameters. Across the 20–100 GPa window, the m-NG supercell evolves from a graphite-like to a diamond-like configuration. The average C–C coordination number (cutoff 1.8 Å) increases from ~2.7 at 20 GPa toward ~3.4 at 100 GPa, while the mean C–C–C bond angle shifts from ~120° (sp^2-like) to ~111° (close to the 109.5° of sp^3 carbon) (Fig. 4c, solid lines). The $sp^2 \rightarrow sp^3$ transition occurs at ~78 GPa, which is also confirmed by the orientational tetrahedral order parameter (Extended Data Fig. 5g) that indicates the formation of a diamond-like structure at this pressure. These trends mirror experimental XRD, Raman and HRTEM observations. The electronic density of states (Fig. 4d) shows a gradual disappearance of π-states and the emergence of a σ-bandgap of ~4 eV. Simulated lattice spacings match experimental values, reinforcing the mechanism."*

Page 13, Lines 267-283 (Results): "To assess the role of temperature, we employed a machine-learning interatomic potential (MLIP). At 0 K, the simulations reproduce the $sp^2 \rightarrow sp^3$ transition at around 93 GPa, consistent with the ab initio results (Extended Data Fig. 5e; Extended Data Video MLIP_0K). Increasing the temperature markedly lowers the transition pressure to 60 GPa at 200 K (Extended Data Fig. 5f; Extended Data Video MLIP_200K) and to 42 GPa at 1,500 K (Fig. 4b; Extended Data Video MLIP_1500K). This trend is reflected in the systematic shift to lower pressures observed in the C–C coordination numbers and C-C-C bond-angle distributions at 200 K (Extended Data Fig. 5h, dashed line), and 1,500 K (Fig. 4c, dashed line) compared with the ab initio results at 0 K (Extended Data Fig. 5h; Fig. 4 c, solid line).

At elevated temperatures, the MLIP simulations reveal enhanced atomic motion at the edges of the m-NG and pronounced molecular bending (Extended Data Video MLIP_1500K). Analysis of bending and $sp^2 \rightarrow sp^3$ transition at 1,500 K shows that strong bending develops within the m-NG and precedes the onset of the $sp^2 \rightarrow sp^3$ transition (Fig. 4b; Extended Data Video MLIP_1500K). Compared with the 0 K ab initio simulations, finite temperature substantially alters the transformation pathway by introducing reactive regions within the highly bent m-NG. We note that the transition pressures obtained from the rapid compression simulations (100 ps) likely overestimate those under experimental conditions."

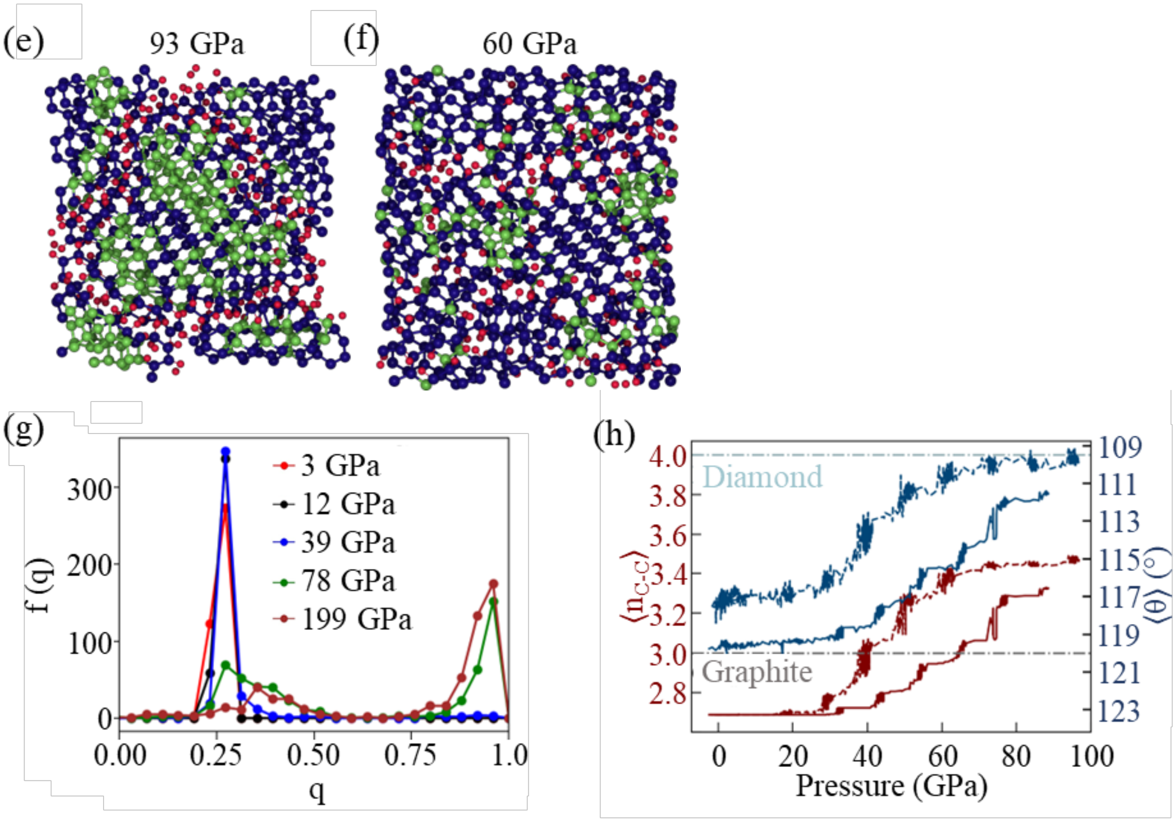

Extended Data Fig. 5: Computational analyses of diamond formation. e, f MLIP simulations of the m-NG supercell at 0 K and 93 GPa and 200 K and 60 GPa. Blue denotes sp^2-hybridized carbon, green denotes sp^3-hybridized carbon, and red denotes hydrogen. **g,** Distribution f(q) of the orientational order parameter q. Following the literature on liquid water[4], we computed $q = 1 - \frac{3}{8}\sum_{j=1}^{3}\sum_{k=j+1}^{4}(cos\,\psi_{jk} + \frac{1}{3})^2$, the orientational tetrahedral order parameter where for a given C atom, i, the angles ψ_{jk} with vertex i and segments ij and ik are calculated for the four nearest neighbours of i. The limits q->0 and q->1 (high pressure) correspond to a fully disordered and a perfect tetrahedral arrangement, respectively. **h**, MLIP-derived average carbon–carbon coordination number ⟨nC-C⟩ (red) and average C-C-C bond angle ⟨θ⟩ (blue) of the m-NG supercell at 200 K (solid line) and 1,500 K (dashed line) as a function of pressure from 0 to 100 GPa. Dashed horizontal lines mark the limiting values for ideal graphite (⟨n⟩ = 3, θ = 120°) and diamond (⟨n⟩ = 4, θ = 109.5°). Compared with 200 K (60 GPa), increasing the temperature to 1,500 K shifts the transition to lower pressure (42 GPa).

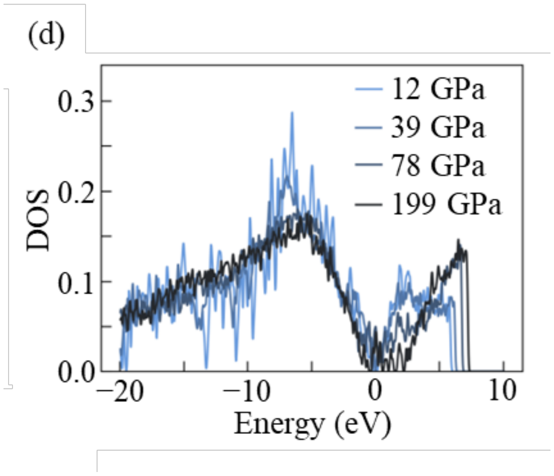

Fig. 4: Pressure-induced transformation of nanographene into diamond-like structures. d, Electronic density of states (DOS) for different values of pressure in our *ab initio* simulations. A gap of approximately 4 eV fully develops at 199 GPa, consistent with the gap reported for diamond within the GGA approximation (5.45 eV).

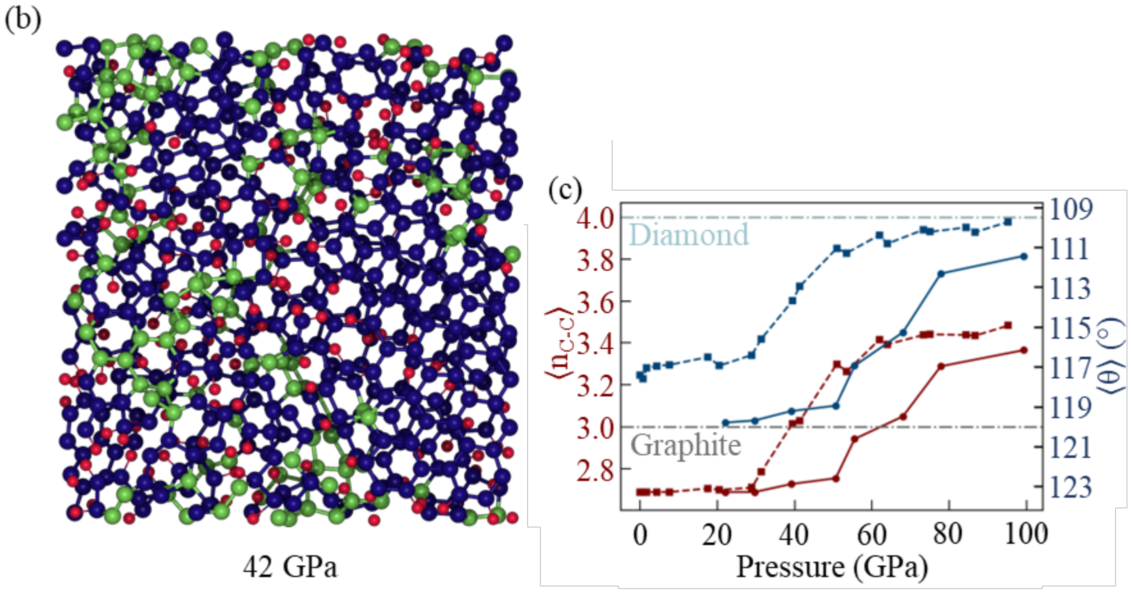

Fig. 4: Pressure-induced transformation of nanographene into diamond-like structures.

b, MLIP simulations of the m-NG supercell at 1,500 K and 42 GPa, showing that diamond nucleation occurs at lower pressures with increasing temperature. Blue denotes sp^2-hybridized carbon, green denotes sp^3-hybridized carbon, and red denotes hydrogen. **c,** Average carbon–carbon coordination number $\langle n_{C\text{-}C} \rangle$ (red) and average C-C-C bond angle $\langle \theta \rangle$ (blue) of the m-NG supercell at 0 K (solid line, *ab initio*) and 1,500 K (dashed line, MLIP) as a function of pressure from 0 to 100 GPa. Dashed horizontal lines mark the limiting values for ideal graphite ($\langle n \rangle = 3$, $\theta = 120°$) and diamond ($\langle n \rangle = 4$, $\theta = 109.5°$). The monotonic evolution toward diamond-like coordination reflects the progressive formation of tetrahedral carbon which occurs at lower pressures as temperature increases.

Q2: A technicality, but consequential: The authors state that molecular structures under pressure did not converge, terminating calculations at forces < 0.2 Ry/bohr, or 5 eV/A, obviously a very large force! This is either a typo or a serious flaw in describing/explaining the process. Forces of this magnitude (enough to break strong covalent bond) indicate unoptimized structures far from the energy minimum, rendering any parameters derived from such calculations (e.g., coordination numbers, bond angles) physically meaningless. Then the claim of a "direct transition" from m-NG to m-ND lacks theoretical, mechanistic support. Consider possibilities (1) above and (4) below?

We thank the reviewer for raising this important technical point. The apparent inconsistency arose from an ambiguity in how the force convergence criterion was reported in the original manuscript. Specifically, we inadvertently reported the norm of the full 3N-dimensional force vector (N = 756 atoms), rather than the maximum force component acting on any individual atom, which is the standard convention in electronic-structure calculations.

The originally reported threshold therefore corresponded to the vector sum of all forces and does not reflect the atomic force criterion typically used to assess structural convergence. The correct value, obtained by dividing the full-vector norm by N, lies nearly three orders of magnitude lower than the number quoted in the previous version.

To avoid any ambiguity, we have fully reoptimised all structures in our *ab initio* simulations and now explicitly report the conventional convergence metric. In the revised calculations, the maximum atomic force component is < 0.001 Ry/Bohr (≈ 0.026 eV/Å) for all optimised configurations. This level of convergence is standard for high-pressure ab initio studies and ensures that:

- All intermediate and final structures lie very close to their local energy minima,
- Derived structural descriptors, coordination numbers, bond-angle distributions, tetrahedral order parameters, are physically meaningful, and
- the mechanistic interpretation of a direct, chemically facilitated $sp^2 \rightarrow sp^3$ transition remains fully supported.

These revised and strictly converged calculations strengthen the robustness of our theoretical analysis and confirm that the simulated transformation pathway is internally consistent and compatible with the experimental results.

The new MLIP simulations are fully consistent with the existing *ab initio* simulations: using quasi-static compression, we obtain similar transition pressure (93 GPa, Extended Data Fig. 4e). Using molecular dynamics at elevated temperatures (200 K and 1500 K) the transition pressure drops substantially to 60 GPa and 42 GPa, respectively (Fig. 4b, c, Extended Data Fig. 5f, h).

Changes to the manuscript: *(Methods, Page 27, Lines 617-623) "In the case of graphite, all steps converge with a total force smaller than 0.01 Ry/Bohr, all components of all forces are smaller than 0.001 Ry/Bohr, and energy smaller than 10-6 Ry. The m-NG structures were converged such that the maximum atomic force component was below 0.001 Ry/Bohr. This ensures that the intermediate and final structures are close to their local minima and that all structural descriptors (coordination numbers, bond-angle distributions, tetrahedral order) are physically meaningful."*

Q3. Furthermore, the temperature clearly plays a significant role in the formation process but seems completely ignored in simulations, performed calculations being only at T = 0.

We thank the reviewer for emphasising the importance of temperature. Temperature plays a crucial role in the experimental nanographene-to-diamond transition. However, the challenge lies in the fundamental limitations of *ab initio* molecular dynamics (AIMD) for describing first-order phase transformations at elevated temperatures.

AIMD trajectories are typically restricted to 10^2–10^3 ps, whereas diamond nucleation under HPHT conditions is governed by rearrangements occurring over µs–ms timescales, i.e., 6–9 orders of magnitude longer. At these longer times, the key bond-breaking, hydrogen-migration, and $sp^2 \rightarrow sp^3$ rehybridisation events relevant to diamond formation cannot be captured. Even advanced enhanced-sampling methods or reactive potentials struggle to provide a quantitatively reliable description of bond rearrangements, defect evolution, and hydrogen mobility in hydrogen-rich C_xH_Y systems.

For these reasons, our *ab initio* simulations were designed to extract zero-temperature structural pathways, providing access to:

- the intrinsic local geometric evolution (coordination numbers, C–C–C bond-angle distributions, tetrahedral order parameters),
- the electronic reconstruction (progressive disappearance of π-states, opening of the σ-band gap), and
- the lattice-scale emergence of diamond-like spacings (D(111) ≈ 2.06 Å),

all of which are directly corroborated by the ED-XRD, Raman, HRTEM, and EELS datasets.

Thus, despite the absence of explicit thermal sampling, the T = 0 K *ab initio* simulations reveal the energetic pathways and structural motifs consistent with the experimentally observed transformation.

Following the reviewer's comment, we additionally performed exploratory finite-temperature simulations using reactive empirical and machine-learning potentials (AIREBO-M, MACE-OFF, MACE-MatPES-PBE, MACE-MatPES-R2SCAN). These methods allow longer trajectories: tens to hundreds of nanoseconds with AIREBO and nanoseconds with MLIPs such as MACE. The reactive empirical potentials and the foundational MLIPs did not produce a complete $sp^2 \rightarrow sp^3$ conversion under accessible time scales and P–T conditions, reflecting both limited sampling and deficiencies in existing potentials for hydrogenated carbon systems. As described above in response to Q2, using a MACE model fine-tuned on our *ab initio data*, we obtained nearly complete $sp^2 \rightarrow sp^3$ conversion at substantially lower transition pressures compared with the zero temperature case.

Changes to the manuscript: *Page 13, Lines 267-283 (Results): "To assess the role of temperature, we employed a machine-learning interatomic potential (MLIP). At 0 K, the simulations reproduce the $sp^3 \rightarrow sp^3$ transition at around 93 GPa, consistent with the ab initio results (Extended Data Fig. 5e; Extended Data Video MLIP_0K). Increasing the temperature markedly lowers the transition pressure to 60 GPa at 200 K (Extended Data Fig. 5f; Extended Data Video MLIP_200K) and to 42 GPa at 1,500 K (Fig. 4b; Extended Data Video MLIP_1500K). This trend is reflected in the systematic shift to lower pressures observed in the C–C coordination numbers and C-C-C bond-angle distributions at 200 K (Extended Data Fig. 5h, dashed line), and*

1,500 K (Fig. 4c, dashed line) compared with the ab initio results at 0 K (Extended Data Fig. 5h; Fig. 4 c, solid line).

At elevated temperatures, the MLIP simulations reveal enhanced atomic motion at the edges of the m-NG and pronounced molecular bending (Extended Data Video MLIP_1500K). Analysis of bending and $sp^2 \rightarrow sp^3$ transition at 1,500 K shows that strong bending develops within the m-NG and precedes the onset of the $sp^2 \rightarrow sp^3$ transition (Fig. 4b; Extended Data Video MLIP_1500K). Compared with the 0 K ab initio simulations, finite temperature substantially alters the transformation pathway by introducing reactive regions within the highly bent m-NG. We note that the transition pressures obtained from the rapid compression simulations (100 ps) likely overestimate those under experimental conditions."

Q4. The ab initio model in Fig. 4a-c assumes infinite molecular stacks under not very realistic periodic conditions, ignoring T-effects and H-engagement. This poorly approximates the experimental system. If ab initio methods cannot even converge (not to mention limiting simulation size and time), semi-empirical potentials readily available for C-H systems (AIREBO, ReaxFF, or recent machine-learning like DeepMD) could be employed to model polycrystalline nucleation from molecular CxHy precursors. The current simulations mostly fail to bridge theory and experiment.

We thank the reviewer for this thoughtful and constructive critique. We have substantially expanded our theoretical section to clarify our methodological choices, strengthen the connection to experiment, and incorporate new simulations.

Justification of the initial stacked nanographene geometry

The stacked configuration is not an arbitrary modelling choice: ED-XRD measurements (Extended Data Fig. 2) unambiguously show that m-NG precursors self-assemble into layered stacks with an interlayer spacing of ~3.35 Å, essentially identical to graphite. The *ab initio* simulations were therefore designed to probe the structural response of experimentally observed precursor assemblies, rather than isolated molecules.

Ab initio simulations: now fully converged and expanded

As noted in Q3, all structures are now converged to < 0.001 Ry/Bohr maximum atomic force. The revised analysis includes:

- Coordination number evolution,
- C–C–C bond-angle distributions,
- Tetrahedral orientational order parameter q (Extended Data Fig. 5b), and
- projected electronic density of states (DOS) (Fig. 4e),

revealing a clear loss of π-states and the emergence of a widening σ-band gap.

These electronic signatures provide explicit evidence that the simulated structures undergo a genuine graphite-to-diamond transition.

Additional P–T simulations using AIREBO-M and state-of-the-art ML potentials

In response to the reviewer, we conducted extensive large-scale simulations using out-of-the-box models:

- AIREBO-M (high-pressure bond-order potential),
- MACE-MatPES-PBE, MACE-MatPES-R2SCAN,
- MACE-OFF
- MLIP (MACE-MH-1),

at T = 2500 K, up to 35 GPa, and for tens to hundreds of nanoseconds.

The first three potentials did not produce diamond nucleation. The angle distributions (see Supportive Figures below) show partial trends towards tetrahedrality under pressure (especially using the MACE models), but average C−C coordination remains ≈ 3, indicating incomplete conversion.

These outcomes reflect known limitations:

- hydrogenated carbon systems remain challenging for ML potentials,
- diamond nucleation was not included in the training sets,
- accessible time scales remain insufficient to capture rare nucleation events.

We have fine-tuned a MACE foundation model using our available *ab initio* data and the resulting model indeed shows complete conversion to 4-fold coordination. See responses to Q1 above. The combination of *ab initio* simulations and ML simulations (after fine-tuning) provide reliable mechanistic insight into the molecule-to-diamond conversion process.

Changes to the manuscript: *Page 12, Lines 247-257(Results) "We analysed the simulations by tracking coordination numbers, bond-angle distributions and tetrahedral order parameters. Across the 20–100 GPa window, the m-NG supercell evolves from a graphite-like to a diamond-like configuration. The average C–C coordination number (cutoff 1.8 Å) increases from ~2.7 at 20 GPa toward ~3.4 at 100 GPa, while the mean C–C–C bond angle shifts from ~120° (sp^2-like) to ~111° (close to the 109.5° of sp^3 carbon) (Fig. 4c, solid lines). The $sp^2 \rightarrow sp^3$ transition occurs at ~78 GPa, which is also confirmed by the orientational tetrahedral order parameter (Extended Data Fig. 5g) that indicates the formation of a diamond-like structure at this pressure. These trends mirror experimental XRD, Raman and HRTEM observations. The electronic density of states (Fig. 4d) shows a gradual disappearance of π-states and the emergence of a σ-bandgap of ~4 eV. Simulated lattice spacings match experimental values, reinforcing the mechanism."*

Page 13, Lines 267-283 (Results): "To assess the role of temperature, we employed a machine-learning interatomic potential (MLIP). At 0 K, the simulations reproduce the $sp^2 \rightarrow sp^3$ transition at around 93 GPa, consistent with the ab initio results (Extended Data Fig. 5e; Extended Data Video MLIP_0K). Increasing the temperature markedly lowers the transition pressure to 60 GPa at 200 K (Extended Data Fig. 5f; Extended Data Video MLIP_200K) and to 42 GPa at 1,500 K (Fig. 4b; Extended Data Video MLIP_1500K). This trend is reflected in the systematic shift to lower pressures observed in the C–C coordination numbers and C-C-C bond-angle distributions at 200 K (Extended Data Fig. 5h, dashed line), and 1,500 K (Fig. 4c, dashed line) compared with the ab initio results at 0 K (Extended Data Fig. 5h; Fig. 4 c, solid line).

At elevated temperatures, the MLIP simulations reveal enhanced atomic motion at the edges of the m-NG and pronounced molecular bending (Extended Data Video MLIP_1500K). Analysis of bending and $sp^2 \rightarrow sp^3$ transition at 1,500 K shows that strong bending develops within the m-NG and precedes the onset of the $sp^2 \rightarrow sp^3$ transition (Fig. 4b; Extended Data Video MLIP_1500K). Compared with the 0 K ab initio simulations, finite temperature substantially alters the

transformation pathway by introducing reactive regions within the highly bent m-NG. We note that the transition pressures obtained from the rapid compression simulations (100 ps) likely overestimate those under experimental conditions."

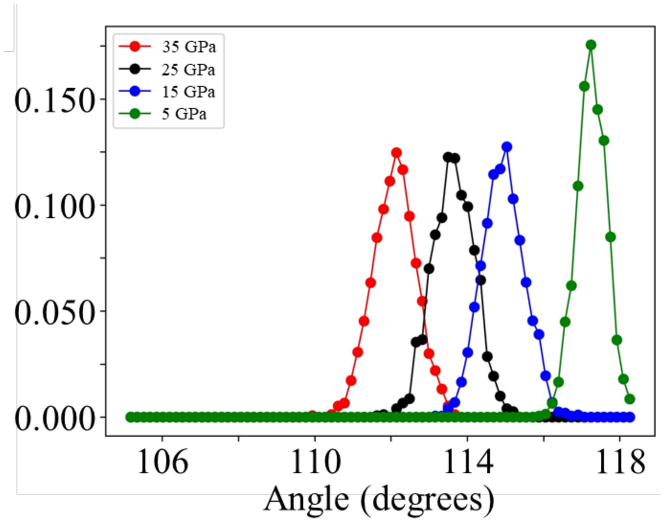

Supportive Figure: C-C-C angle distribution as a function of pressure for the stack of nanographenes at T=2500 K. Simulations were carried out using the MACE-MatPES-PBE potential[5] at 2500 K.

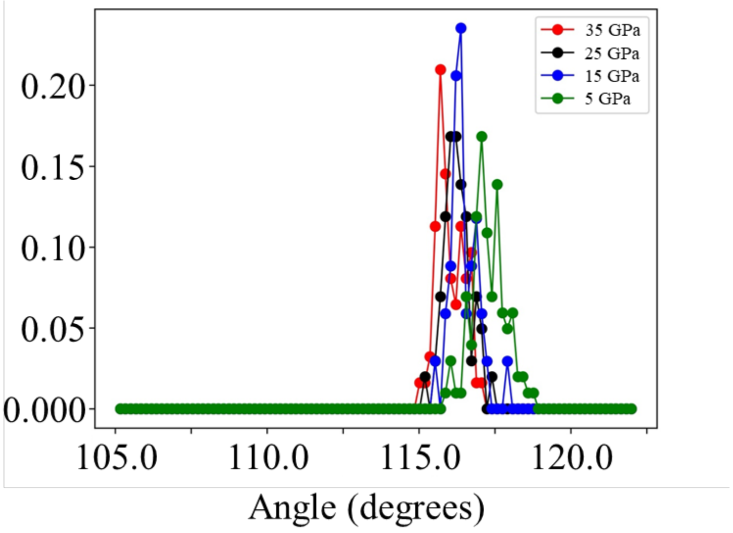

Supportive Figure: C-C-C angle distribution as a function of pressure. Simulations were carried out using the AIREBO-M potential[6] at 2500 K.

Q5. The proposed spherical morphology is at odds with the triangular geometry of m-NG. No mechanism is offered to explain how a m-NG layered precursor transforms into isotropic nanoparticles.

We thank the reviewer for raising this important point. The 2D triangular geometry of the m-NG is not directly relevant, because each nanodiamond is formed from a number of stacked m-NGs,

resulting in a roughly spherical shape (aspect ratio of diameter/height = 1.2 with comparable lateral dimensions and heights derived from TEM and AFM). Based on the new HR-TEM and EELS analysis (Extended Data Fig. 4), both crude and acid-treated m-NDs clearly appear as isotropic, faceted nanoparticles in HRTEM, with well-defined diamond lattice fringes extending to the particle surface (Fig. 2c, Extended Data Fig. 1c).

- All particles are isotropic and faceted, with no residual signature of the triangular precursor geometry.
- EELS spectral imaging confirms that only a single sp^2-rich monolayer remains at the surface, with the diamond core fully relaxed into an sp^3 lattice.

Obviously, the triangular shape of m-NG is not preserved under HPHT transformation for three mechanistic reasons:

Early loss of precursor geometry during $sp^2 \rightarrow sp^3$ collapse

- Under ~1200 °C and ~13 GPa, the molecular precursor does not convert through a rigid structural transformation. Instead, out-of-plane distortions occur rapidly.
- π-conjugation is broken locally.
- C atoms rehybridise from trigonal (sp^2) to tetrahedral (sp^3) coordination.

ab initio simulations show that once this collapse begins, the triangular π-framework is no longer structurally meaningful.

Thermodynamic relaxation toward minimal surface free energy

For nanodiamonds containing a fixed number of carbon atoms and a fixed amount of hydrogen:

- The equilibrium morphology that minimises total surface free energy is a compact, faceted, quasi-spherical diamond particle, not a remnant of the original triangular precursor.

The 2D triangular geometry of the m-NG is not directly relevant, because each nanodiamond is formed from a number of stacked m-NGs, resulting in a roughly spherical shape under pressure. This is visible on the top and side views from the MLIP molecular dynamics trajectories at 1,500 K, which are part of the Extended Data in the revised version.

Hydrogen plays a decisive role: its limited mobility in diamond (diffusion coefficient extremely small, Cherniak et al.[7]) constrains surface reconstruction early during growth. Thus, once hydrogen passivates the emerging sp^3 surfaces, the particle relaxes into the lowest-energy shape permitted by the available hydrogen.

HPHT conditions favour isotropic diamond nuclei

At the onset of diamond nucleation, growth does not proceed by templating the precursor geometry. Instead:

- Diamond nuclei adopt isotropic shapes determined by surface energy minimisation.
- Grain coalescence and facet formation occur during annealing, further erasing precursor geometry.

- The experimentally observed lattice fringes confirm the formation of fully relaxed, crystalline diamond domains rather than molecularly imprinted morphologies.

In summary, the triangular geometry of m-NG is dynamically irrelevant to the morphology of the final nanodiamond. Both experiment (HRTEM, EELS) and theory (coordination/bond-angle evolution, tetrahedral order formation) support a transformation mechanism in which (i) the precursor rapidly loses its planar geometry during rehybridisation, (ii) nanodiamond nuclei form with isotropic shapes dictated by surface energy, (iii) Hydrogen passivation locks in the final, faceted morphology.

Changes to the manuscript: *Page 6, Lines 128-139 (Results): "HRTEM images of both crude (Fig. 2c) and acid-treated (Extended Data Fig. 1 c) m-NDs reveal highly crystalline, faceted, approximately equiaxed nanoparticles. A single sp2 surface reconstruction is visible, appearing partially detached in some regions and giving rise to slightly wavy edges (Fig. 2c). EELS spectral imaging (Extended Data Fig. 4) confirms that this reconstruction is sp^2-rich, and the presence of such a thin reconstruction (<0.5 nm) is notable compared with the higher sp^2 fractions typically reported for nanodiamonds of similar size[34]. These combined results indicate that m-NDs possess minimal graphitic carbon and high structural purity.*

As the sp^2 network collapses under HPHT conditions, the structure relaxes toward a morphology with minimal surface free energy. Given the fixed hydrogen content and the low diffusivity of hydrogen in diamond, the available hydrogen reservoir dictates surface-passivation and therefore the final ND size, yielding uniform nanoparticles."

(c)
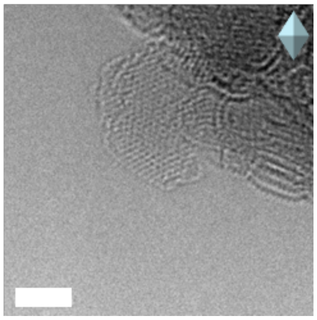

Fig. 2 c: HRTEM micrograph of m-ND, evidencing high crystallinity and a single sp^2 surface reconstruction. Scale bar, 2 nm.

(c)
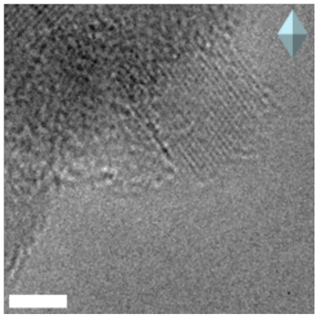

Extended Data Fig. 1 c: HRTEM micrograph of m-ND_{ox}, evidencing high crystallinity and a single sp^2 surface reconstruction. Scale bar, 2 nm.

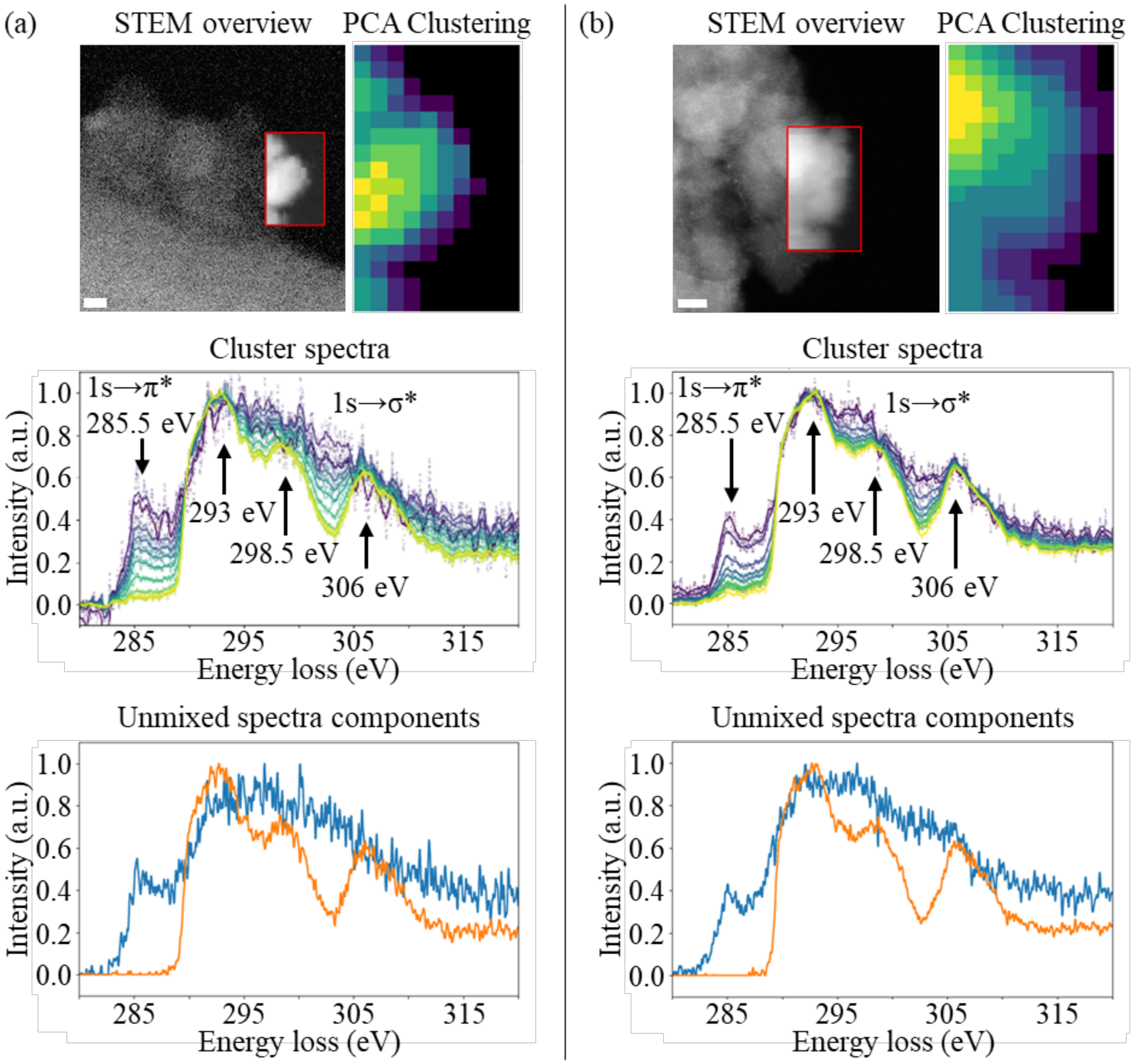

Extended Data Fig. 4: Site-specific EELS analysis of nanodiamond bonding and surface structure. a, EELS analysis of m-ND using a 0.5 nm steps Spectral Image (SI). The pixels in the SI were clustered in 10 regions (and an additional vacuum one) using PCA and k-means clustering on the EELS spectra. The average spectra of the clusters, shown normalised, were then used to reconstruct the EELS spectra of the surface (blue) and bulk (orange) components. The presence of the bulk component in the outermost region indicates that sp^2-layer thickness does not exceed 0.5 nm. ***b***, the same analysis was performed on m-ND_{ox}, showing qualitatively the same results. Scalebars are 2 nm.

Q6. The correlation of the size distribution with the H/C ratio is logical and can be experimentally tested/proven by the use of C42H18 (already used in some parts of the manuscript) as its H/C ~ 0.42 should correspond to nanodiamond size of ~2 nm, distinct from 3 nm for C96H30, H/C ~ 0.31.

We thank the reviewer for this insightful suggestion. While the logic of testing the [H]/[C] model using $C_{42}H_{18}$ is compelling, our experiments demonstrate that $C_{42}H_{18}$ does not follow the behaviour predicted by the m-NG–based model. Instead, HPHT conversion of $C_{42}H_{18}$ produces substantially larger and highly polydisperse particles (20–350 nm) under identical HPHT conditions (13 GPa, 1200 °C). This behaviour indicates that the original [H]/[C] ratio alone cannot be treated as a universal predictive parameter, and that thermal stability of precursor is a critical prerequisite for the model to apply.

<u>The [H]/[C] model applies only when the precursor remains structurally intact until the onset of HPHT-induced transformation</u>

$C_{96}H_{30}$ (m-NG):

- Retains structural integrity up to ~950 °C (TGA; Extended Data Fig. 7a).
- Does not convert into graphite before diamond nucleation (ED-XRD, Fig. 3a, d).
- Provides a well-defined hydrogen reservoir that passivates emerging sp^3 surfaces and limits ND growth.

Under these conditions, hydrogen availability is the dominant size-regulating factor, and the model reliably predicts ultrasmall ND formation (~3–4 nm with std/mean = 0.18).

<u>$C_{42}H_{18}$ decomposes prematurely, invalidating the mechanistic assumptions of the model</u>

In contrast, $C_{42}H_{18}$ behaves analogously to coronene and other small polycyclic aromatics:

- Thermal decomposition at ~680 °C (TGA; Extended Data Fig. 7a).
- Loss of structural integrity well before HPHT diamond nucleation.

In situ ED-XRD of coronene ($C_{24}H_{12}$) clearly shows:

- Loss of molecular peaks at ~800 °C (Extended Data Fig. 7b).
- Emergence of the graphite (002) reflection between 900–1000 °C (Extended Data Fig. 7b).
- Full consistency with prior high-P/T studies (Jennings et al.[8]).

Thus, we assume $C_{42}H_{18}$ cannot be meaningfully compared to intact m-NG, nor can its size outcome be predicted solely from stoichiometric hydrogen content.

<u>Why the model must not be interpreted as universal</u>

We now clarify in the manuscript that the [H]/[C] model:

- Applies only to the intact one-component m-NG precursor under the specific HPHT conditions in this work.
- Does not hold for precursors that decompose before $sp^2 \rightarrow sp^3$ transformation.
- Does only qualitatively apply to doped (two-component) systems where dopant-derived carbon decomposes and lowers the effective hydrogen availability. Therefore, doped systems involve multiple decomposition pathways, heterogeneous generation of amorphous carbon, dopant-carbon interactions, altered interfacial energies, and

accelerated Ostwald ripening. The reduced hydrogen availability could also lead to fewer surface terminations, which could result in larger ND sizes, as observed experimentally

To summarize, the [H]/[C] model intentionally describes the relationship between hydrogen availability and growth limitation in a regime where the precursor remains chemically intact, and is not intended as a general predictor across molecular carbon systems.

Changes to the manuscript: (Discussion, Page 11, Lines 226-234) *"The [H]/[C] model applies specifically to the intact $C_{96}H_{30}$ precursor under the HPHT conditions used here. m-NG is stable up to the onset of diamond formation, ensuring a well-defined hydrogen reservoir for surface passivation. Smaller or less stable precursors, such as coronene or HBC, decompose before diamond formation, altering hydrogen availability and generating amorphous carbon that promotes uncontrolled growth (Extended Data Fig. 7). Consequently, these precursors do not follow stoichiometric size predictions and yield larger, more heterogeneous nanodiamonds. Likewise, in two-component dopant systems, decomposition of the dopant precursor and modified nucleation kinetics decouple the final nanodiamond size from the initial [H]/[C] ratio, clarifying the scope and limits of the H/C model."*

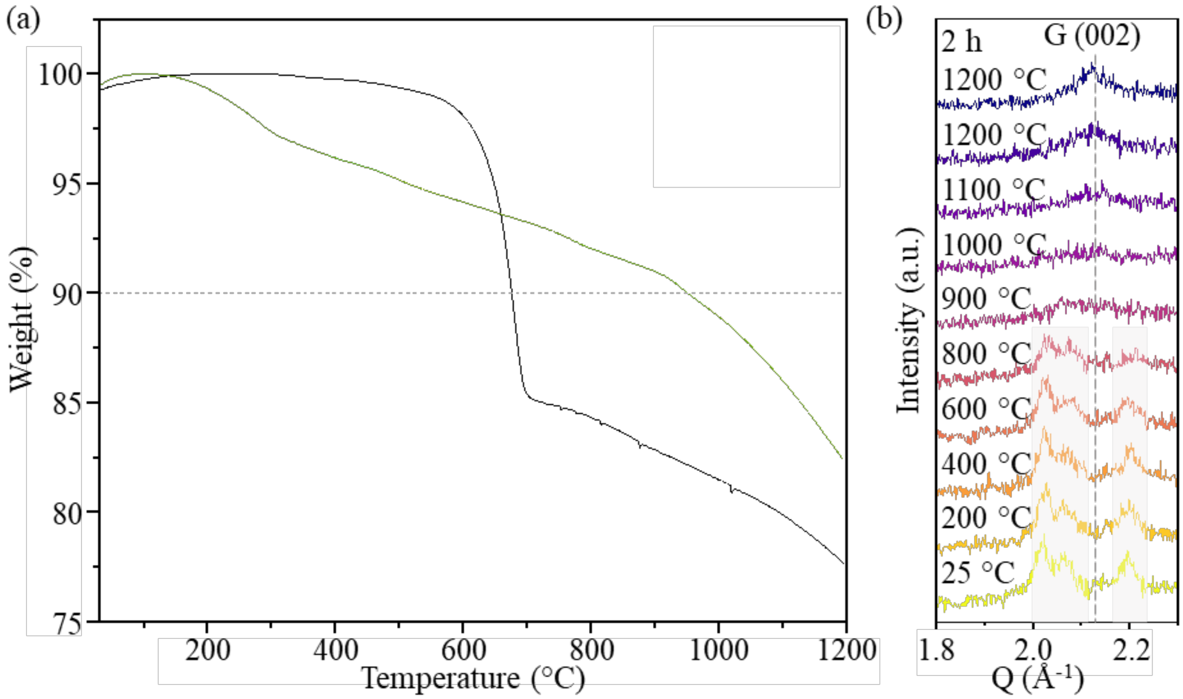

Extended Data Fig. 7: Stability analyses of ND precursors HBC, m-NG and coronene. a, TGA curve of HBC (black) and m-NG (green). A weight loss of 10% (dashed line) was measured at 680 °C (HBC) and 950 °C (m-NG), respectively. **b,** *In situ* ED-XRD of coronene at 13 GPa. Increasing the temperature from 25 °C up to 1200 °C results in the disappearance of the characteristic peaks of coronene (grey box) above 800 °C. Graphite formation starts between 800 and 900 °C depicted by the appearance of the (002) peak of graphite (grey dashed line). The peak becomes more evident after maintaining the temperature at 1200 °C for 2 h.

Q7. No explanation is provided on increased diamond size formed from smaller molecules. Furthermore, no comment was made on a significant increase in nanodiamond size with the addition of Si and Ge dopants. SiV-NDs (29±8 nm) are tenfold (~103 in volume?) larger than m-NDs (3.1±0.6 nm), yet the text offers no explanation. Does doping alter nucleation kinetics? This oversight weakens the mechanistic narrative.

We thank the reviewer for this important comment. We agree that the substantial increase in nanodiamond size upon doping requires explicit mechanistic clarification. In the revised manuscript, we now provide a unified explanation showing that precursor stability, hydrogen availability, and dopant-induced kinetic effects together account for the observed size differences.

<u>The one-component m-NG system produces ultrasmall NDs because the precursor remains intact up to the HPHT transition</u>

$C_{96}H_{30}$ (m-NG):

- Remains structurally intact up to ~950 °C (TGA; Extended Data Fig. 7a).
- Does not undergo graphitisation prior to diamond nucleation (ED-XRD, Fig. 3a, d).
- Preserves a well-defined stoichiometric hydrogen content.

Under these conditions, hydrogen acts as a growth-limiting reagent, passivating emerging sp^3 surfaces and restricting crystal size to ~3–4 nm with extremely narrow dispersity (std/mean = 0.18). This tightly controlled regime is the basis of the [H]/[C] model.

<u>In the doped two-component systems, dopant precursors decompose before diamond nucleation</u>

The SiV- and GeV-fNDs are synthesised from nanographene + dopant precursor, fundamentally altering the reaction environment.

The dopant precursors:

- Tetrakis(trimethylsilyl)silane (Si precursor): melts at 319–321 °C;
- Tetraphenylgermane (Ge precursor): melts at 230–235 °C; Thus, long before diamond nucleation, these molecules decompose to yield:

- Amorphous/sp^2-rich carbon,
- Additional reactive carbon, and
- A dramatically reduced effective hydrogen availability.

This destroys the stoichiometric hydrogen regime that limits growth in the one-component system. As a result, doped nanodiamonds grow much more extensively, yielding:

- SiV-fNDs: 29 ± 8 nm
- GeV-fNDs: 80–150 nm

This trend is precisely consistent with the predictions of the [H]/[C] model when hydrogen availability decreases.

Dopant atoms actively modify nucleation kinetics and favour the growth of larger crystallites

Beyond altering the hydrogen balance, dopants also change the nucleation landscape. Silicon and germanium alter interfacial energy and promote coalescence as demonstrated by Crane et al.[9]:

- Si lowers the diamond–carbon interfacial energy,
- Accelerates grain coalescence, and
- Promotes Ostwald ripening, where larger crystallites grow at the expense of smaller ones.

This mechanism naturally explains the size increase of SiV-fNDs relative to m-NDs.

Why the model does not predict doped ND size quantitatively

Because dopant precursors decompose prematurely and introduce additional amorphous carbon, the doped system no longer preserves a fixed hydrogen reservoir. Thus:

- The [H]/[C] model remains valid only for intact $C_{96}H_{30}$,
- And provides qualitative (but not quantitative) insight into doped systems.

Therefore, the much larger size of doped fluorescent nanodiamonds is a direct consequence of precursor decomposition prior to nucleation, reduced hydrogen availability, dopant-induced lowering of interfacial energy, and accelerated coalescence and ripening. Together these effects explain the observed size disparity between m-NDs and SiV/GeV-fNDs.

Changes to the manuscript: *Page 18, Lines 379-384 (Discussion): "By fixing both the carbon framework and the hydrogen reservoir at the molecular level, this approach intrinsically constrains crystal growth, enabling predictive size selection in the low-nanometre regime, a capability not accessible to existing HPHT, CVD or detonation methods. Extending this design to two-component precursor mixtures yields SiV^- and GeV^- fNDs in a single step, without implantation, annealing or mechanical processing, and with markedly narrower size and defect distributions than prior methods."*

Q8: While the main achievement here is m-NDs synthesis, the main value is in m-ND functionality as the hosts-particles for SiV^- and GeV^- defects, with demonstrated photoluminescence (PL) of zero-phonon line (ZPL) emission near 738 nm and 603 nm. These wavelengths are consistent with previously reported values for the same defects in bulk diamond. However, while these ZPL positions may be familiar to researchers working specifically on diamond defects, they are not widely known outside this field. The authors should therefore cite relevant literature from bulk diamond studies to support this identification.

We thank the reviewer for this helpful comment. We agree that, while the zero-phonon line (ZPL) positions of the SiV^- and GeV^- centers are well established within the diamond–quantum-defect community, these values may not be immediately familiar to a broader readership. To ensure clarity and accessibility, we have now added citations to the foundational bulk-diamond studies that report and spectroscopically confirm the characteristic ZPL wavelengths for these color centers.

SiV⁻ center (737–738 nm)

We now reference seminal studies demonstrating the ZPL of the negatively charged silicon-vacancy center at ~737–738 nm in high-purity bulk diamond:

- Rogers et al.[10] – room-temperature and cryogenic ZPL characterization
- Hepp et al.[11] – detailed electronic-structure analysis and charge-state identification

GeV⁻ center (602–603 nm)

For the negatively charged germanium-vacancy center, we include references documenting its characteristic ZPL at ~602–603 nm:

- Iwasaki et al.[12] – first experimental identification in bulk diamond
- Nadolinny et al.[13] – theoretical and spectroscopic insight into the electronic states

These additions make the assignment of the observed emission features explicit even for readers not specialised in diamond-defect spectroscopy. They also provide the appropriate experimental context to support our identification of SiV⁻ and GeV⁻ centers in our bottom-up synthesized fluorescent nanodiamonds.

Changes to the manuscript: *Page 16, Lines 322-323 (Results): "The room-temperature PL spectrum (Fig. 5b) shows the SiV⁻ zero-phonon line (ZPL) at 738 nm[45,46], confirming incorporation of the defect."*

Results, Page 16, Line 338: "Room-temperature PL shows a strong GeV⁻ ZPL at 603 nm (Fig. 5f)[50,51],…"

Q9. Additionally, the authors assert that the synthesized defects are in a negative charge state, which is not obvious or granted. They could comment on the origin of this charge, identify possible donor species responsible for providing the extra electron. Evaluating the corresponding CTL (charge transition level) and its position w.r.t. "Fermi level" may also be expected. This is particularly relevant given that their synthesis procedure does not appear to introduce obvious contaminants or impurities.

We thank the reviewer for raising this important point regarding charge-state stabilisation. While the ZPL wavelengths observed in our PL spectra (SiV: 738 nm; GeV: 603 nm) unambiguously correspond to the negatively charged SiV⁻ and GeV⁻ centers, as the neutral charge states exhibit distinctly different ZPLs (e.g., $SiV^0 \approx 946$ nm), we agree that the mechanism by which these negative charge states are stabilised should be explicitly discussed.

Origin of donor electrons: unintentional nitrogen incorporation under HPHT conditions

Although no intentional dopant is introduced, the HPHT synthesis occurs in an environment dominated by atmospheric nitrogen. As in natural and synthetic diamonds, substitutional nitrogen (P1 centers) is therefore the most probable unintentional donor species.

This interpretation is directly supported by our EPR measurements (Extended Data Fig. 6 a, b):

- GeV-fNDs exhibit clear hyperfine-split P1 signatures, consistent with neutral substitutional nitrogen (S = ½).

- SiV-fNDs do not show the P1 signal, but this is fully consistent with nitrogen being present in the ionized EPR-silent state (N^+) after donating its electron to the SiV center.

These observations establish the presence of electron donors capable of stabilizing negatively charged group-IV color centers.

<u>Charge-transition levels (CTLs) confirm stabilisation of SiV^- and GeV^-</u>

Theoretical work (e.g., Gali et al.[14]) provides essential context:

- The $SiV^- \rightarrow SiV^0$ CTL lies deep in the band gap, making the negative charge state robust even in weakly doped or nanoscale materials.
- The GeV^- CTL also lies deep, slightly shallower than SiV^- but significantly deeper than that of NV^-.
- By contrast, the NV^- CTL is located much closer to the valence band, making NV^- highly sensitive to surface acceptor states and prone to ionization in ultrasmall nanodiamonds.

This hierarchy of CTLs ($SiV^- > GeV^- >> NV^-$) explains our key observations:

- SiV^- and GeV^- remain stable and produce strong ZPL emission.
- NV^- is absent despite the confirmed presence of nitrogen donors, fully consistent with its known instability in small nanodiamonds.

<u>Mechanistic consistency</u>

Combining PL, EPR, and CTL arguments:

- Donor electrons originate from unintentional nitrogen incorporated during HPHT processing.
- The deep CTLs of SiV and GeV centers ensure that these defects remain preferentially in the negative charge state.
- The absence of NV^- emission, despite detectable P1 donors, is entirely consistent with its less favourable CTL position.

Thus, the experimental evidence and theoretical CTL considerations together provide a coherent and mechanistically robust explanation for why the SiV and GeV centers produced in our nanodiamonds appear in their negative charge states.

Changes to the manuscript: *Page 16, Lines 342-344 (Results): "Charge-state identification is supported by the presence of donor species: EPR spectra of GeV-fNDs show P1 centres, while the absence of NV^- emission reflects the deeper charge-transition levels of SiV^- and GeV^- in weakly compensated material."*

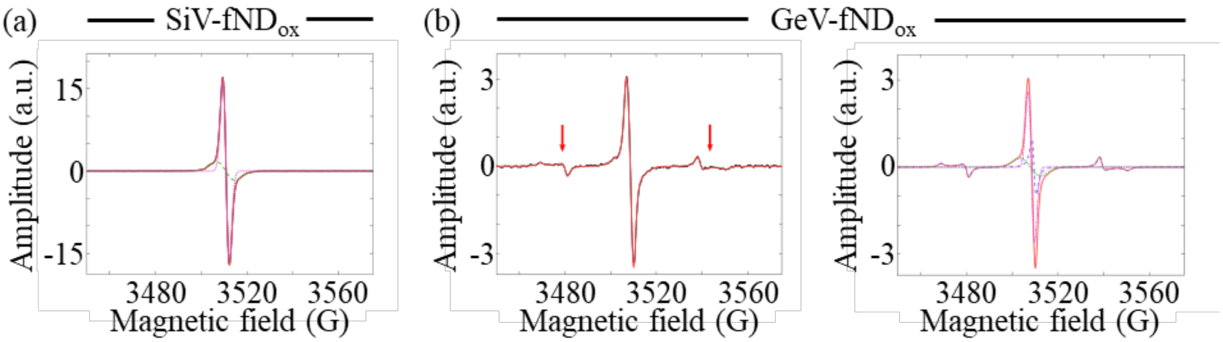

Extended Data Fig. 6: Spectral and optical properties of fluorescent nanodiamonds and control experiments. a and **b,** X-band continuous-wave EPR spectra and fits, for SiV- and GeV-doped sample. **a**, SiV-doped sample: experimental spectrum (black line) and fit (red line). The fit is performed with two components, labelled according to their relative linewidth as 'broad' (green, dashed) and 'narrow' (purple, dotted). The fit yields g=2.0031 and g=2.0030 for the broad and the narrow component, and corresponding peak-to-peak linewidths of 0.80 mT and 0.28 mT. **b**, Spectrum and fit for the GeV-doped sample. Left subfigure: Experimental spectrum (black line) and fit with three contributions (red line): g~2 components (broad and narrow), and substitutional nitrogen (P1 centers). Arrows indicate the groups of P1 hyperfine lines corresponding to the nuclear spin state of the ^{14}N, m_I = -1,+1. Right subfigure: Representation of the individual fit contributions. A microwave frequency of 9.84 GHz and a microwave power of 4.73 μW were used in the measurements.

References:

1. Sun, Y., Kvashnin, A. G., Sorokin, P. B., Yakobson, B. I. & Billups, W. E. Radiation-Induced Nucleation of Diamond from Amorphous Carbon: Effect of Hydrogen. J. Phys. Chem. Lett. 5, 1924–1928 (2014).
2. Erohin, S. V., Ruan, Q., Sorokin, P. B. & Yakobson, B. I. Nano‐Thermodynamics of Chemically Induced Graphene–Diamond Transformation. Small 16, (2020).
3. Correa, A. A., Bonev, S. A. & Galli, G. Carbon under extreme conditions: Phase boundaries and electronic properties from first-principles theory. Proc. Natl. Acad. Sci. 103, 1204–1208 (2006).
4. Errington, J. R. & Debenedetti, P. G. Relationship between structural order and the anomalies of liquid water. Nature 409, 318–321 (2001).
5. Kaplan, A. D., Liu, R., Qi, J., Ko, T. W. & Deng, B. A Foundational Potential Energy Surface Dataset for Materials. 1–3.
6. O'Connor, T. C., Andzelm, J. & Robbins, M. O. AIREBO-M: A reactive model for hydrocarbons at extreme pressures. J. Chem. Phys. 142, (2015).
7. Cherniak, D. J., Watson, E. B., Meunier, V. & Kharche, N. Diffusion of helium, hydrogen and deuterium in diamond: Experiment, theory and geochemical applications. Geochim. Cosmochim. Acta 232, 206–224 (2018).

8. Jennings, E., Montgomery, W. & Lerch, P. Stability of Coronene at High Temperature and Pressure. J. Phys. Chem. B 114, 15753–15758 (2010).

9. Crane, M. J. et al. High-pressure, high-temperature molecular doping of nanodiamond. Sci. Adv. 5, eaau6073 (2019).

10. Rogers, L. J. et al. Multiple intrinsically identical single-photon emitters in the solid state. Nat. Commun. 5, 4739 (2014).

11. Hepp, C. et al. Electronic Structure of the Silicon Vacancy Color Center in Diamond. Phys. Rev. Lett. 112, 036405 (2014).

12. Iwasaki, T. et al. Germanium-Vacancy Single Color Centers in Diamond. Sci. Rep. 5, 12882 (2015).

13. Nadolinny, V. A. et al. EPR study of germanium-vacancy defects in diamonds. J. Struct. Chem. 57, 1041–1043 (2016).

14. Gali, A. & Maze, J. R. Ab initio study of the split silicon-vacancy defect in diamond: Electronic structure and related properties. Phys. Rev. B 88, 235205 (2013).

Referee #1 (Remarks to the Author):

Authors have taken into account most of the previous comments and the overall quality of the manuscript was greatly improved. Nevertheless, several comments need to be considered before the publication of the manuscript.

We thank the reviewer for the positive feedback on the overall improvement of our revised manuscript. We agree that the analysis of the nanodiamond surface required clarification and have revised the manuscript accordingly (see below).

Q1: Abstract, Introduction and Discussion« Here we show that well-defined, hydrogen-terminated molecular nanographenes serve as chemically confined precursors for high-pressure, high-temperature synthesis of ultrasmall (3–4 nm), monodisperse and highly crystalline molecular nanodiamonds (m-NDs) containing a hydrogen-terminated surface with only a single sp^2 surface reconstruction""FTIR spectra (Extended Data Fig. 3a) reveal distinct C–H stretching modes centred around 2830 cm^{-1} and, together with the positive zeta potential of +8.2 ± 0.6 mV (Extended Data Fig. 3f), confirm hydrogen termination of m-ND"

According to the FTIR spectra, very weak C-H signature can be seen compared to spectra of hydrogenated nanodiamonds (Miliaieva et al. Nanoscale Advances 2023; Saoudi et al. Carbon 2023). Moreover, the related zeta potential exhibits a low positive value (less than 10 mV). Other surface termination of nanodiamonds can lead to positive zeta potential (sp2 terminated, OH- terminated,…). These FTIR / zeta results underline a partial coverage by C-H bonds, such bonds are not the major part of the functional groups. Authors cannot talk about a hydrogen-terminated surface. This statement should be modified in the abstract, the introduction and the discussion.

We agree that the FTIR spectra show only a weak C–H signature compared with strongly hydrogenated nanodiamonds reported in the literature (e.g., Miliaieva et al.[1], Saoudi et al.[2]). In addition, the measured zeta potential exhibits only a small positive value (<10 mV), which further suggests that the surface is not exclusively C-H terminated. To avoid overinterpretation, we have revised the manuscript accordingly. In the abstract, introduction, and discussion, the phrases about "a hydrogen-terminated surface" has been removed and/or modified to "a partial coverage by C-H bonds on the surface" to interpret the FTIR and zeta results more accurately.

The corresponding revisions have been implemented throughout the manuscript.

Changes to the Manuscript:

Page 2, Lines 35–38 (Abstract): "Here we show that well-defined, hydrogen-terminated molecular nanographenes serve as chemically confined precursors for high-pressure, high-temperature synthesis of ultrasmall (3–4 nm), monodisperse and highly crystalline molecular nanodiamonds (m-NDs) with only a single sp^2 surface reconstruction and produced on a milligram scale."

Page 4, Lines 82–84 (Introduction): "Using a catalyst-free transformation, we obtain ultrasmall (3–4 nm), monodisperse m-NDs with high structural purity and a single sp^2 surface reconstruction."

Page 6, Lines 120–122 (Results): "FTIR spectra (Extended Data Fig. 3a) reveal C–H stretching modes centred around 2830 cm^{-1} and, together with the positive zeta potential of +8 ± 1 mV (Extended Data Fig. 3f), suggest a partial coverage by C-H bonds on the surface of m-NDs[35]."

Page 12, Lines 227–229 (Results): "Predicted clusters expose mixed (111), (100) and (311) facets (Fig. 4f, inset), consistent with XRD and SAED; FTIR confirms partial coverage by C-H bonds on the m-ND surface."

Page 18, Lines 369–372 (Discussion): "In contrast to graphite, which remains kinetically trapped and yields multiphase mixtures at 13 GPa and 1,200 ℃, hydrogen-terminated nanographenes undergo rapid and complete $sp^2 \rightarrow sp^3$ rehybridisation, producing 3–4 nm monodisperse nanodiamond crystals with a single sp^2 surface reconstruction."

Q2: "A single sp2 surface reconstruction is visible, appearing partially detached in some regions and giving rise to slightly wavy edges (Fig. 2c)"This sp2 reconstruction should lead to a component located at - 1eV from the sp3 one in the C1s XPS (see references on hydrogenated nanodiamonds in comment 1). It has not been considered by authors in their provided fits (Extended Data Fig. 3). Authors should consider this and provide the quantitative analysis of the C1s fit (% of area for the carbon binding states) for m-ND and m-NDOx.

In order to contribute to the concerns of the reviewer, we have now carried out a more quantitative analysis of the C1s XPS spectra for m-ND and m-ND_{ox}. The C1s components were analysed according to the methods described by Miliaieva et al.[1], and Saoudi et al.[2] and deconvoluted into contributions from C–C (sp^3), C=C (sp^2), C–O and C=O species. The relative proportions of these C1s components are now also shown in the XPS panels in Extended Data Fig. 3b,c as well as in the table (Extended Data Fig. 3 g).

As shown in Extended Data Fig. 3b,c, the fitted spectra indicate a comparable C=C contribution of 18% for m-ND and 16% for m-ND_{ox}, with the corresponding component shifted by approximately −1 eV (−0.7 eV for m-ND and −0.9 eV for m-NDox), consistent with partial sp^2 surface reconstruction. The proportion of the C=C bonds arise because XPS from such small particles (<10 nm) is strongly surface-weighted: the surface carbon (including sp^2) disproportionally contributes to the total C1s signal compared with the bulk-like sp^3 signal.[3] **Hence, the presence of C=C contributions in both nanodiamond samples is consistent with the single sp^2 surface reconstruction observed by EELS** (Fig. 2f, Extended Data Fig. 4). Oxidation of the nanodiamond surface further increases the C=O contribution from 2% to 5%, which is also consistent with the negative zeta potential of m-ND_{ox} (Extended Data Fig. 3f) and suggests an increased abundance of carboxyl-containing surface groups. Additionally, the increase of C=O after oxidization is depicted in the FTIR spectra (Extended Data Fig. 3a). In contrast, the C–O contribution decreases slightly from 10% to 7%. The presence of C=C, C–O and C=O species in both m-ND and m-ND_{ox}, together with the FTIR analysis, indicates that the nanodiamond surface is only partially hydrogenated and also contains additional functional groups. We have therefore revised the statement regarding hydrogen- terminated surface and rephrased it in accordance with the reviewer's comment.

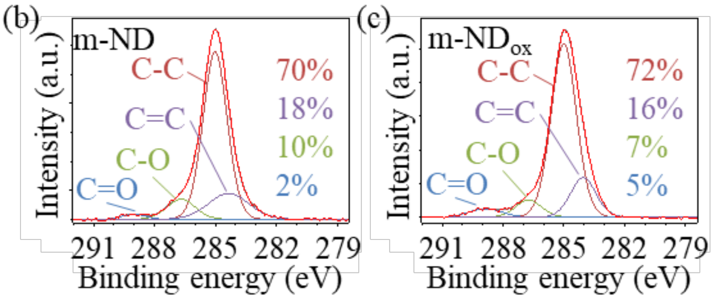

(g)

		C-C	C=C	C-O	C=O
m-ND	Binding energy (eV)	285.0	284.3	286.7	289.0
	Area (%)	70	18	10	2
m-ND$_{ox}$	Binding energy (eV)	285.0	284.1	286.8	288.9
	Area (%)	72	16	7	5

Changes to the Manuscript: *The revisions regarding C–H termination are described in our response to Reviewer 1, Q1.*

Page 6, Lines 133–134 (Results): "Deconvolution of the C1s XPS spectra confirms the presence of C=C species in both m-ND and m-ND$_{ox}$ (Extended Data Fig. 3b–e, g)."

Q3: Some values should be according to the resolution or the measurement accuracy: "characteristic diamond peak at 1329.3 cm^{-1}" 1329 cm-1, according to the spectral resolution
"+8.2 ± 0.6 mV » 8 ± 1 mV according to the measurement accuracy, apply to other values: "−15.4 ± 0.5 mV »« +10.7 ± 0.7 mV to −20.9 ± 0.5 mV »

We thank the reviewer for this helpful remark. We have carefully checked the resolution or the measurement accuracy of the instruments and rounded the values accordingly through the whole manuscript.

Q4: All references should be checked (all co-authors listed)

All author names have been included in the reference list in accordance with Nature journal style, which requires listing all authors when there are five or fewer. For references with more than five authors, only the first author is listed, followed by "et al.", as specified in the journal's instructions.

Nature Formatting Guide (https://www.nature.com/nature/for-authors/formatting-guide): "*All authors should be included in reference lists unless there are more than five, in which case only the first author should be given, followed by 'et al.'.*"

Referee #2 (Remarks to the Author):[see also attached pdf]

I think the authors have beautifully answered all the questions raised, and now the data are very convincing that ultrasmall, highly size- and shape-uniform nanodiamonds were synthesized in a bottom-up approach. I therefore recommend publication of the manuscript. Other than the scientific issues, I have some further points that would improve the quality of the presentation.

Q1: The HR-TEM image can be magnified to this level as follows (attached) to show the surface sp² layer more clearly. Also, a pointing arrow or something similar could help readers understand it.

We thank the reviewer for this helpful suggestion and have replaced Fig. 2c with a zoomed-in view of the m-ND particle, including an arrow indicating the sp² surface reconstruction. Additionally, we have also included an arrow in the HRTEM image of m-ND_{ox} (Extended Data Fig. 1c).

(c)

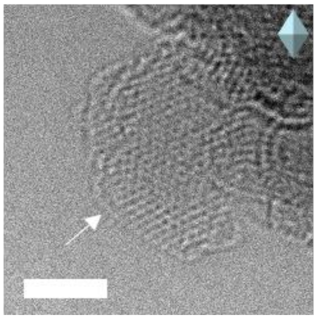

Fig 2: Nanographene-to-nanodiamond transformation under HPHT conditions. c, HRTEM micrograph of m-ND, evidencing high crystallinity and a single sp² surface reconstruction, indicated by the arrow. Scale bar, 2 nm.

(c)

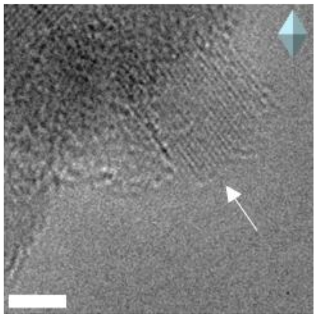

Extended Data Fig. 1: Comparison of HPHT treated hexabenzocoronene and m-NG. c, HRTEM micrograph of m-ND_{ox}, evidencing high crystallinity and a single sp² surface reconstruction, indicated by the arrow. Scale bar, 2 nm.

Q2: This diamond symbol in Fig. 2b-e perhaps indicates diamond data, but it is hard to catch the information. I think these marks are not necessary, or the meaning should be written in the captions.

We thank the reviewer for this suggestion. The diamond symbols in the figures were intended to denote data measured on m-ND samples. we agree that the meaning of these markers is not immediately clear from the figure and could be confusing. We now added a clear description in the figure caption, "**Blue diamond symbols indicate nanodiamond measurements**."

Q3: The newly measured EELS data (Extended Data Fig. 4) are striking and very convincing for the formation of diamond and the sp² surface structure. I think these data could be included in the main figures as part of Fig. 2. However, I leave this to the authors' decision.

We thank the reviewer for the positive feedback regarding on the EELS data. We agree that these data convincingly demonstrate the sp² surface reconstruction and have followed the reviewer's suggestion by including the EELS data for m-ND in the main text Fig. 2f. Accordingly, the Extended Data Figure 4 has been revised to include only the EELS analysis of m-ND_{ox}.

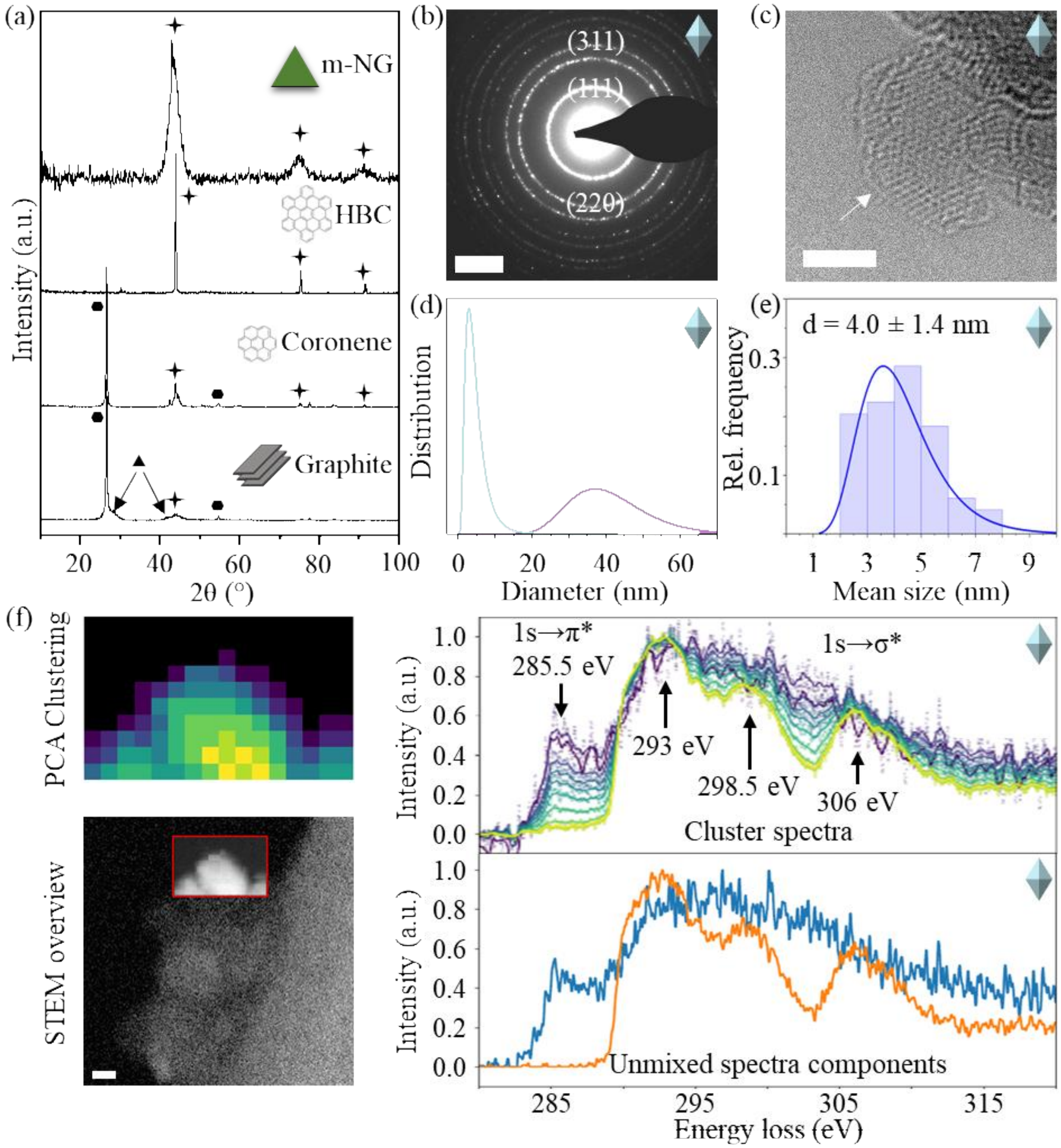

Fig. 2: Fig. 2| Nanographene-to-nanodiamond transformation under HPHT conditions. a, XRD patterns of HPHT-treated precursors: (1) graphite yields metastable carbon (triangle) and unreacted graphite (hexagon); (2) coronene converts partially into diamond (star); (3) HBC and (4) m-NG convert fully into diamond. **b,** Selected-area electron diffraction (SAED) pattern of m-NDs (scale bar, 5 nm^{-1}). **c,** HRTEM micrograph of m-ND, evidencing high crystallinity and a single sp^2 surface reconstruction, indicated by the arrow. Scale bar, 2 nm. **d**, SAXS analysis of m-NDs (light blue) compared with a commercial 30 nm milled HPHT ND sample (purple). **e**, size distribution according to HRTEM micrographs (d = 4.0 ± 1.4 nm, N = 49). **f**, EELS analysis of m-ND using a spectral image acquired with a 0.5 nm step size. Pixels were clustered into 10 regions (plus vacuum) by PCA and k-means analysis of the EELS spectra. The normalized average cluster spectra were used to reconstruct the surface (blue) and bulk (orange) components. The presence of the bulk component in the outermost region indicates that the sp^2 surface reconstruction does not exceed 0.5 nm. Scale bar, 2 nm. Blue diamond symbols indicate nanodiamond measurements.

Referee #3 (Remarks to the Author):

In this second view i do nnaturally ot follow all the A-H items recommended. I think the authors have addressed most of concerns thoroughly, to my satisfaction at least.

1) Ref 41 is a good one, but I bet must also include the book title, as "…in 'Nucleation in Condensed Matter', pp 19-54…' Without it just a chapter title alone "The Classical Theory" reads hollow out of the context (theory of what?). Format this ref properly. Alos in ref 40 pages ## seem missing?

We thank the reviewer for pointing this out. The former Reference 41 (Book section "The Classical Theory"[4]) has been updated to include the book title, editors, page range, publisher, and year, following Nature journal style. The former Reference 40 (Erohin et al.[5]) has been corrected to include the complete page range.

Q2: Understandably one of the central surprises here remains why the needed pressure appears so much lower than would be expected in "common" graphite-diamond transformation, the authors may benefit from seeing the phase diagram Fig. 1 of Nano Lett. dx.doi.org/10.1021/nl403938g If space permits it could be valuable ref to add for the readers too.

We thank the reviewer for this insightful comment. We have consulted the suggested reference[6] and agree that its phase diagram (Fig. 1) provides useful context for understanding the transformation conditions of hydrogenated graphene-like precursors. Accordingly, we have added this reference in our manuscript as reference 41.

Overall, the work main result is quite interesting and it can be published, with minor additions/improvements (1-2) above if paper goes for another round of quick revision.

We sincerely thank all reviewers for their careful reading of our manuscript and for their constructive comments, which have helped us to further improve the clarity and quality of the work.

References:

1. Miliaieva, D. *et al.* Absolute energy levels in nanodiamonds of different origins and surface chemistries. *Nanoscale Adv.* **5**, 4402–4414 (2023).

2. Saoudi, L. *et al.* Colloidal stability over months of highly crystalline high-pressure high-temperature hydrogenated nanodiamonds in water. *Carbon N. Y.* **202**, 438–449 (2023).

3. Ducrozet, F. *et al.* New Insights into the Reactivity of Detonation Nanodiamonds during the First Stages of Graphitization. *Nanomaterials* **11**, 2671 (2021).

4. Kelton, K. F. & Greer, A. L. The Classical Theory. in *Nucleation in Condensed Matter* 19–54 (Elsevier Ltd, Amsterdam, 2010). doi:10.1016/S1470-1804(09)01502-8.

5. Erohin, S. V., Ruan, Q., Sorokin, P. B. & Yakobson, B. I. Nano-Thermodynamics of Chemically Induced Graphene–Diamond Transformation. *Small* **16**, 2070256 (2020).

6. Kvashnin, A. G., Chernozatonskii, L. A., Yakobson, B. I. & Sorokin, P. B. Phase Diagram of Quasi-Two-Dimensional Carbon, From Graphene to Diamond. *Nano Lett.* **14**, 676–681 (2014).

Referee 2 Reviewer Attachment 1 Version 0:

The manuscript presents a bottom-up synthesis method for producing molecular nanodiamonds (m-NDs) by applying a HPHT process to mNG ($C_{96}H_{30}$). The authors demonstrate the ND formation with control over particle size and morphology, and report the successful incorporation of color defect centers such as SiV and GeV by simply adding dopant precursors to the reaction. The process is supported by a combination of experimental techniques including SAXS, AFM, TEM, in situ ED-XRD, and complemented by ab initio calculations that provide insights into the growth mechanism and structure of the resulting NDs. The manuscript is well written. I believe the proposed synthesis method is interesting and has the potential to impact the development of high-quality ultrasmall NDs for quantum applications. However, I believe several aspects of the manuscript require further clarification and more rigorous experimental validation. The main claims related to size control, material purity, and surface chemistry are not sufficiently supported by the current data. Therefore, I cannot recommend the manuscript for publication in its current form.

1. The authors claim that the bottom-up HPHT process allows for improved control over ND size compared to conventional top-down techniques such as milling or detonation. However, the experimental data does not convincingly demonstrate this improvement. Fig 1c and Ext. Data Fig. 1f,i show particle size distributions measured by different techniques such as AFM, SAXS, and TEM. However the values of mean and standard deviation differ across these methods, and the manuscript does not discuss the reasons for this variation. A quantitative comparison of size dispersion, for instance by calculating and comparing the standard deviation-to-mean (std/mean) ratio for each method and sample, can help support the claim. It would also be helpful to compare these parameters to those of conventional NDs. In addition, the use of Raman spectroscopy to correlate particle size with peak position and linewidth, particularly the diamond band, could strengthen the discussion. Previous studies [Mermoux et al. Diamond Rel. Mater. 87, 248–260, (2018)] have reported such dependencies, and including an alaysis like that reinforces the manuscript's arguments about size control and crystallinity.

2. Although size is discussed extensively, the morphology of the NDs is not discussed. This is a significant omission, as shape can strongly influence properties such as fluorescence brightness and optical homogeneity. Conventional top-down NDs often exhibit rod- or plate-like shapes (e.g., Eldemrdash et al., Carbon 206, 268 (2023); Oshimi et al., ACS Nano 18, 35202 (2024); Haotian et al., ACS Nano 17, 16491 (2023)). If the present synthesis method truly enables morphological uniformity, this would be an important advantage, but the manuscript does not present data to demonstrate this. In addition, the TEM images provided have relatively low magnification (e.g., 20 nm scale bars), which is not sufficient for assessing particle shape or observing lattice patterns. Given that the manuscript refers to HR-TEM, higher-resolution images should be provided to show lattice structures extending to the surface of particles. Such data could also reveal the presence of sp^2-rich surface layers or graphitic contamination. Additionally, performing statistical shape analysis on a representative number of particles is valuable to support the claim of morphological control.

3. The presence of GR1 centers shown in Fig 5c may indicate lattice defects or imperfections. A more discussion of GR1 centers, and ideally, further data should be included to assess the defect situations in these NDs more comprehensively. Further, EPR measurements is useful in this context, as they can quantify vacancy and impurity defects. Since the manuscript emphasizes the scalability of the synthesis method, it is reasonable to expect that sufficient sample quantities can be produced for EPR analysis.

4. For ultrasmall NDs, bulk-based techniques such as Raman and XRD are not sufficient to fully characterize the NDs. Electronic structure and bonding should be studied using more site-specific or surface-sensitive methods such as EELS or XAS. As many properties of ultrasmall NDs deviated from the bulk diamond crystals, the site-specific observations on the electronic structure will unambiguously address exact

material situations of the present ND samples [Chang et al., ACS Nano 16, 8513 (2022). Ref. 23 in the present manuscript]. The authors need to perform EELS or XAS for their samples to fully confirm the acquisition of high quality NDs.

5. The NDs containing SiV and GeV centers have significantly larger sizes (~30 nm) than the undoped m-NDs (~3 nm). This raises an important question about whether doping inherently leads to larger crystal growth, or whether it reflects an intentional or uncontrollable change in synthesis conditions. Since size and defect properties are central to the paper, a discussion on the cause of this difference is needed. Also, the above thorough study on the size dependence in combination with this defect-included NDs can strengthen the paper. Further, the applicability of the proposed [H]/[C] ratio model to doped NDs is not explained. Does this model still predict or control the size in the doped case? If not, why? The authors should clarify whether and how their theoretical framework can be extended to describe dopant-containing systems. If the size increase is unavoidable due to the requirements of incorporating SiV or GeV centers, this should be stated and justified clearly.

6. The strain in NDs can significantly affect the optical properties of color centers. For SiV centers, in particular, the width and distribution of ZPL can be used as an indicator of strain. The manuscript can be improved by analyzing the ZPL linewidths and comparing them with previous studies, such as Lindner et al. (New J. Phys. 20, 115002, 2018. Ref. 40 in the present manuscript), which relate optical line broadening to local strain fields through ab initio modeling. Providing this type of analysis would not only strengthen the interpretation of defect quality but also highlight the quantum optical properties.

7. The authors describe a hydrogenated ND surface in their theoretical model, but it is unclear whether the synthesized particles are actually hydrogen-terminated after HPHT processing. The manuscript does not describe the collection process or mention whether any surface cleaning or termination steps were applied after the synthesis. Since the surface strongly influences both optical properties and chemical stability, details on this point are essential. IR spectroscopy and XPS are necessary to characterize surface terminations and functional groups. These measurements would also help clarify how well the actual material matches the model assumptions.

Minor Comments

- In the abstract, the term “superphenalene” is used, while the title refers to “nanographene.” It is better to clarify this terminology, since coronene, HBC, and m-NG are actually used in the experiments.
- Line 221: “Fig. 4d” → “Fig. 4e.”
- In Ext. Data Fig. and captions, notations such as “(a+b)” are confusing and should be revised to specify each panel explicitly.
- The use of labels like “Gottingen” and “MPIP” should be avoided or replaced with more meaningful ids with captions. And I can’t understand what these four NDs mean.

Referee 2 Reviewer Attachment 1 Version 1:

I think the authors have beautifully answered all the questions raised, and now the data are very convincing that ultrasmall, highly size- and shape-uniform nanodiamonds were synthesized in a bottom-up approach. I therefore recommend publication of the manuscript. Other than the scientific issues, I have some further points that would improve the quality of the presentation.

1. The HR-TEM image can be magnified to this level as follows to show the surface sp^2 layer more clearly. Also, a pointing arrow or something similar could help readers understand it.

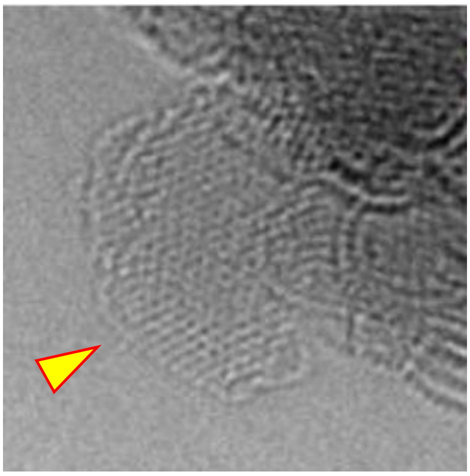

2. This diamond symbol in Fig. 2b-e perhaps indicates diamond data, but it is hard to catch the information. I think these marks are not necessary, or the meaning should be written in the captions.

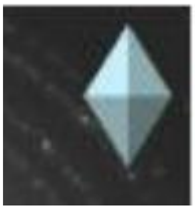

3. The newly measured EELS data (Extended Data Fig. 4) are striking and very convincing for the formation of diamond and the sp^2 surface structure. I think these data could be included in the main figures as part of Fig. 2. However, I leave this to the authors' decision.
